# Sequence Compression Benchmark (SCB) database—A comprehensive evaluation of reference-free compressors for FASTA-formatted sequences

**DOI:** 10.1093/gigascience/giaa072

**Published:** 2020-07-06

**Authors:** Kirill Kryukov, Mahoko Takahashi Ueda, So Nakagawa, Tadashi Imanishi

**Affiliations:** Department of Molecular Life Science, Tokai University School of Medicine, Isehara, Kanagawa 259–1193, Japan; Current address: Department of Genomics and Evolutionary Biology, National Institute of Genetics, Mishima, Shizuoka 411-8540, Japan; Department of Molecular Life Science, Tokai University School of Medicine, Isehara, Kanagawa 259–1193, Japan; Current address: Department of Genomic Function and Diversity, Medical Research Institute, Tokyo Medical and Dental University, Bunkyo, Tokyo 113-8510, Japan; Department of Molecular Life Science, Tokai University School of Medicine, Isehara, Kanagawa 259–1193, Japan; Department of Molecular Life Science, Tokai University School of Medicine, Isehara, Kanagawa 259–1193, Japan

**Keywords:** compression, benchmark, DNA, RNA, protein, genome, sequence, database

## Abstract

**Background:**

Nearly all molecular sequence databases currently use gzip for data compression. Ongoing rapid accumulation of stored data calls for a more efficient compression tool. Although numerous compressors exist, both specialized and general-purpose, choosing one of them was difficult because no comprehensive analysis of their comparative advantages for sequence compression was available.

**Findings:**

We systematically benchmarked 430 settings of 48 compressors (including 29 specialized sequence compressors and 19 general-purpose compressors) on representative FASTA-formatted datasets of DNA, RNA, and protein sequences. Each compressor was evaluated on 17 performance measures, including compression strength, as well as time and memory required for compression and decompression. We used 27 test datasets including individual genomes of various sizes, DNA and RNA datasets, and standard protein datasets. We summarized the results as the Sequence Compression Benchmark database (SCB database, http://kirr.dyndns.org/sequence-compression-benchmark/), which allows custom visualizations to be built for selected subsets of benchmark results.

**Conclusion:**

We found that modern compressors offer a large improvement in compactness and speed compared to gzip. Our benchmark allows compressors and their settings to be compared using a variety of performance measures, offering the opportunity to select the optimal compressor on the basis of the data type and usage scenario specific to a particular application.

## Background

Molecular sequence databases store and distribute DNA, RNA, and protein sequences as compressed FASTA-formatted files. Biological sequence compression was first proposed in 1986 [1], and the first practical compressor was made in 1993 [2]. A lively field emerged that produced a stream of methods, algorithms, and software tools for sequence compression [3, 4]. However, despite this activity, currently nearly all databases universally depend on gzip for compressing FASTA-formatted sequence data. This incredible longevity of the 27-year-old compressor probably owes to multiple factors, including conservatism of database operators, wide availability of gzip, and its generally acceptable performance. Through all these years the amount of stored sequence data kept growing steadily [5], increasing the load on database operators, users, storage systems, and network infrastructure. However, someone thinking to replace gzip invariably faces the questions, which of the numerous available compressors to choose? And will the resulting gains even be worth the trouble of switching?

Previous attempts at answering these questions are limited by testing too few compressors and by using restricted test data [6–11]. In addition, all of these studies provide results in the form of tables, with no graphical outputs, which makes the interpretation difficult. Existing benchmarks with useful visualization such as Squash [12] are limited to general-purpose compressors.

The variety of available specialized and general-purpose compressors is overwhelming. At the same time the field was lacking a thorough investigation of the comparative merits of these compressors for sequence data. Therefore we set out to benchmark all available practically useful compressors on a variety of relevant sequence data. Specifically, we focused on the common task of compressing DNA, RNA, and protein sequences, stored in FASTA format, without using reference sequence. The benchmark results are available in the Sequence Compression Benchmark database (SCB database [13]).

## Findings

### Scope, compressors, and test data

We considered the common scenario of archiving, transferring, and working with large datasets of biological sequences. In the present study we did not investigate compression of raw sequencing data in FASTQ format, which was previously thoroughly reviewed [11]. Instead we focused on typical FASTA-formatted datasets, which includes individual genomes and single gene sets. Consequently we also did not consider referential compression, but only reference-free compression, which is typically used for such data. We evaluated stand-alone compression tools (rather than libraries), working under Linux OS on a modern workstation PC. In this study we only consider lossless compression.

We tested all DNA sequence compressors that are available and functional in 2020: dnaX [14], XM [15], DELIMINATE [16], Pufferfish [17], DNA-COMPACT [18], MFCompress [19], UHT [20], GeCo [21], GeCo2 [22], JARVIS [23], NAF [24], and NUHT [25]. We also included the relatively compact among homology search database formats: BLAST [26] and 2bit—a database format of BLAT [27].

Because compressors designed for FASTQ data can be trivially adopted for FASTA-formatted inputs, we also included a comprehensive array of compressors designed primarily or specifically for FASTQ data: BEETL [28], Quip [29], fastqz [10], fqzcomp [10], DSRC 2 [30], Leon [31], LFQC [32], KIC [33], ALAPY [34], GTX.Zip [35], HARC [36], LFastqC [37], SPRING [38], Minicom [39], and FQSqueezer [40]. We also included AC—a compressor designed exclusively for protein sequences [41]. We also tested a comprehensive array of general-purpose compressors: bcm [42], brieflz [43], brotli [44], bsc [45], bzip2 [46], cmix [47], gzip [48], lizard [49], lz4 [50], lzop [51], lzturbo [52], nakamichi [53], pbzip2 [54], pigz [55], snzip [56], xz [57], zpaq [58], zpipe [58], and zstd [59]. See Table 1 for the list of compressors we used.

**Table 1:** Compressor versions

A) Specialized sequence compressors	
Compressor	Version
2bit	"faToTwoBit" and "twoBitToFa" binaries dated 7 November 2018
ac	AC 1.1, 29 January 2020
alapy	ALAPY 1.3.0, 25 July 2017
beetl	BEETL, commit 327cc65, 14 November 2019
blast	"convert2blastmask", "makeblastdb", and "blastdbcmd" binaries from BLAST 2.8.1+, 26 November 2018
dcom	DNA-COMPACT, latest public source 29 August 2013
dlim	DELIMINATE, version 1.3c, 2012
dnaX	dnaX 0.1.0, 3 August 2014
dsrc	DSRC 2.02, commit 5eda82c, 4 June 2015
fastqz	fastqz 1.5, commit 39b2bbc, 15 March 2012
fqs	FQSqueezer 0.1, commit 5741fc5, 17 May 2019
fqzcomp	fqzcomp 4.6, commit 96f2f61, 2 December 2019
geco	GeCo: v.2.1, 24 December 2016
	GeCo2: v.1.1, 2 February 2019
gtz	GTX.Zip PROFESSIONAL-2.1.3-V-2020-03-18 07:11:20, binary
harc	HARC, commit cf35caf, 4 October 2019
jarvis	JARVIS v.1.1, commit d7daef5, 30 April 2019
kic	KIC binary, 0.2, 25 November 2015
leon	Leon, 1.0.0, 27 February 2016, Linux binary
lfastqc	LFastqC, commit 60e5fda, 28 February 2019, with fixes
lfqc	LFQC, commit 59f56e0, 6 January 2016
mfc	MFCompress,s1.01, 3 September 2013, 64-bit Linux binary
minicom	Minicom, commit 2360dd9, 9 September 2019
naf	NAF, 1.1.0, 1 October 2019
nuht	NUHT, commit 08a42a8, 26 September 2018, Linux binary
pfish	Pufferfish, v.1.0 alpha, 11 April 2012
quip	Quip, commit 9165bb5, 1.1.8-8-g9165bb5, 17 December 2017
spring	SPRING, commit 6536b1b, 28 November 2019
uht	UHT, binary from 27 December 2016
xm	XM (eXpert-Model), 3.0, commit 9b9ea57, 7 January 2019
**B) General-purpose compressors**	
bcm	1.30, 21 January 2018
brieflz	1.3.0, 15 February 2020
brotli	1.0.7, 23 October 2018
bsc	3.1.0, 1 January 2016
bzip2	1.0.6, 6 September 2010
cmix	17, 24 March 2019
gzip	1.6, 9 June 2013
lizard	1.0.0, 8 March 2019
lz4	1.9.1, 24 April 2019
lzop	1.04, 10 August 2017
lzturbo	1.2, 11 August 2014
nakamichi	9 May 2020
pbzip2	1.1.13, 18 December 2015
pigz	2.4, 26 December 2017
snzip	1.0.4, 2 October 2016
xz	5.2.2, 29 September 2015
zpaq	7.15, 17 August 2016
zpipe	2.01, 23 December 2010
zstd	1.4.5, 22 May 2020

For the test data, we selected a variety of commonly used sequence datasets in FASTA format: (i) individual genomes of various sizes, as examples of non-repetitive data [60, 61]; (ii) DNA and RNA datasets, such as collections of mitochondrial genomes, influenza virus sequences [60–63], 16S ribosomal RNA gene sequences [64], and genomic multiple DNA sequence alignments [65]; and (iii) standard protein datasets [62, 66–68]. Individual genomes are less repetitive, while other datasets are more repetitive. In total we used 27 test datasets. See Table 2 for the list of test data. All test data are available at the GigaDB repository [69].

**Table 2:** Test datasets

A) Genome sequence datasets				
Category	Organism	Accession	Size	
Virus	*Gordonia*phage GAL1 [61]	GCF_001884535.1	50.7 kB	
Bacteria	WS1 bacterium JGI 0000059-K21 [60]	GCA_000398605.1	522 kB	
Protist	*Astrammina rara* [60]	GCA_000211355.2	1.71 MB	
Fungus	*Nosema ceranae* [60]	GCA_000988165.1	5.81 MB	
Protist	*Cryptosporidium parvum*Iowa II [60]	GCA_000165345.1	9.22 MB	
Protist	*Spironucleus salmonicida* [60]	GCA_000497125.1	13.1 MB	
Protist	*Tieghemostelium lacteum* [60]	GCA_001606155.1	23.7 MB	
Fungus	*Fusarium graminearum*PH-1 [61]	GCF_000240135.3	36.9 MB	
Protist	*Salpingoeca rosetta* [60]	GCA_000188695.1	56.2 MB	
Algae	*Chondrus crispus* [60]	GCA_000350225.2	106 MB	
Algae	*Kappaphycus alvarezii* [60]	GCA_002205965.2	341 MB	
Animal	*Strongylocentrotus purpuratus* [61]	GCF_000002235.4	1.01 GB	
Plant	*Picea abies* [60]	GCA_900067695.1	13.4 GB	
**B) Other DNA datasets**				
**Dataset**	**No. of sequences**	**Size**	**Source**	**Date**
Mitochondrion [61]	9,402	245 MB	RefSeq ftp: ftp://ftp.ncbi.nlm.nih.gov/refseq/release/mitochondrion/mitochondrion.1.1.genomic.fna.gz	15 March 2019
			ftp://ftp.ncbi.nlm.nih.gov/refseq/release/mitochondrion/mitochondrion.2.1.genomic.fna.gz	
NCBI Virus Complete Nucleotide Human [62]	36,745	482 MB	NCBI Virus: https://www.ncbi.nlm.nih.gov/labs/virus/vssi/	11 May 2020
Influenza [63]	700,001	1.22 GB	Influenza Virus Database: ftp://ftp.ncbi.nih.gov/genomes/INFLUENZA/influenza.fna.gz	27 April 2019
Helicobacter [60]	108,292	2.76 GB	NCBI Assembly: https://www.ncbi.nlm.nih.gov/assembly	24 April 2019
**C) RNA datasets**				
SILVA 132 LSURef [64]	198,843	610 MB	Silva database: https://ftp.arb-silva.de/release_132/Exports/SILVA_132_LSURef_tax_silva.fasta.gz	11 December 2017
SILVA 132 SSURef Nr99 [64]	695,171	1.11 GB	Silva database: https://ftp.arb-silva.de/release_132/Exports/SILVA_132_SSURef_Nr99_tax_silva.fasta.gz	11 Devember 2017
SILVA 132 SSURef [64]	2,090,668	3.28 GB	Silva database: https://ftp.arb-silva.de/release_132/Exports/SILVA_132_SSURef_tax_silva.fasta.gz	11 December 2017
**D) Multiple DNA sequence alignments**				
UCSC hg38 7way knownCanonical-exonNuc [65]	1,470,154	340 MB	UCSC: https://hgdownload.soe.ucsc.edu/goldenPath/hg38/multiz7way/alignments/knownCanonical.exonNuc.fa.gz	6 June 2014
UCSC hg38 20way knownCanonical-exonNuc [65]	4,211,940	969 MB	UCSC: https://hgdownload.soe.ucsc.edu/goldenPath/hg38/multiz20way/alignments/knownCanonical.exonNuc.fa.gz	30 June 2015
**E) Protein datasets**				
PDB [66]	109,914	67.6 MB	PDB database FTP: ftp://ftp.ncbi.nih.gov/blast/db/FASTA/pdbaa.gz	9 April 2019
Homo sapiens GRCh38 [67]	105,961	73.2 MB	NCBI ftp: ftp://ftp.ensembl.org/pub/release-96/fasta/homo_sapiens/pep/Homo_sapiens.GRCh38.pep.all.fa.gz	12 March 2019
NCBI Virus RefSeq Protein [62]	373,332	122 MB	NCBI Virus: https://www.ncbi.nlm.nih.gov/labs/virus/vssi/	10 May 2020
UniProtKB Reviewed (Swiss-Prot) [68]	560,118	277 MB	UniProt ftp: ftp://ftp.uniprot.org/pub/databases/uniprot/current_release/knowledgebase/complete/uniprot_sprot.fasta.gz	2 April 2019

### Benchmark

We benchmarked each compressor on every test dataset, except in cases of incompatibility (e.g., DNA compressors cannot compress protein data) or excessive time requirement (some compressors are so slow that they would take weeks on larger datasets). For compressors with adjustable compression level, we tested the relevant range of levels. We tested both 1- and 4-thread variants of compressors that support multi-threading. In total, we used 430 settings of 48 compressors. We also included the non-compressing "cat" command as control. For compressors using non-trivial wrappers, we also benchmarked the wrappers.

Currently many sequence analysis tools accept gzip-compressed files as input. Switching to another compressor may require either adding support of new format to those tools, or passing the data in uncompressed form. The latter solution can be achieved with the help of Unix pipes, if both the compressor and the analysis tool support streaming mode. Therefore, we benchmarked all compressors in streaming mode (streaming uncompressed data in both compression and decompression).

For each combination of compressor setting and test dataset we recorded compressed size, compression time, decompression time, peak compression memory, and peak decompression memory. The details of the method and raw benchmark data are available in the Methods section and Supplementary Data, respectively. We share benchmark results at the online SCB database [13]. All benchmark code is available [70].

The choice of measure for evaluating compressor performance depends on a prospective application. For long-term data storage, compactness may be the single most important criterion. For a public sequence database, the key measure is how much time it takes from initiating the download of compressed files until the decompressed data are accessed. This time consists of transfer time plus decompression time (TD-Time). Corresponding transfer-decompression speed (TD-Speed) is computed as Original Size/TD-Time. In this use case, compression time is relatively unimportant because compression happens only once, while transfer and decompression times affect every user of the database. For a 1-time data transfer, all 3 steps of compression, transfer, and decompression are timed (CTD-Time) and used for computing the resulting overall speed (CTD-Speed).

A total of 17 measures, including the aforementioned ones, are available in our results data (see Methods for the list of measures). Any of these measures can be used for selecting the best setting of each compressor and for sorting the list of compressors. These measures can then be shown in a table and visualized in the form of column charts and scatterplots. This allows the output to be tailored to answer specific questions, such as what compressor is better at compressing a particular kind of data or which setting of each compressor performs best at a particular task. The link speed that is used for estimating transfer times is configurable. The default speed of 100 Mbit/sec corresponds to the common speed of a fixed broadband internet connection.

**Figure 1:**
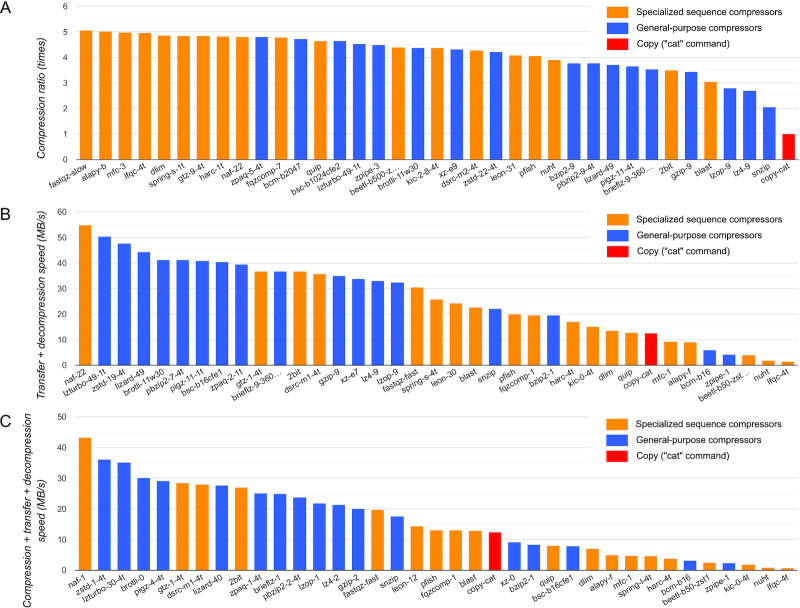
Comparison of 36 compressors on human genome. The best settings of each compressor are selected on the basis of different aspects of performance: (A) compression ratio, (B) transfer + decompression speed, and (C) compression + transfer + decompression speed. The copy-compressor ("cat" command), shown in red, is included as a control. The selected settings of each compressor are shown in their names, after hyphen. Multi-threaded compressors have "-1t" or "-4t" at the end of their names to indicate the number of threads used. Test data are the 3.31 GB reference human genome (accession number GCA_000001405.28). Benchmark CPU: Intel Xeon E5-2643v3 (3.4 GHz). Link speed of 100 Mbit/sec was used for estimating the transfer time.

Fig. 1 compares the performance of the best settings of 36 compressors on the human genome. It shows that specialized sequence compressors achieve excellent compression ratio on this genome. However, when total TD-Speed or CTD-Speed is considered (measures that are important in practical applications), most sequence compressors fall behind the general-purpose ones. The best compressors for this dataset in terms of compression ratio, TD-Speed, and CTD-Speed are "fastqz-slow," "naf-22," and "naf-1," respectively (numbers in each compressor name indicate compression level and other settings). Interestingly, the non-compressing "cat" command used as a control, while naturally showing at the last place on compression ratio (Fig. 1A), is not the slowest in terms of TD-Speed and CTD-Speed (Fig. 1B and C, respectively). In the case of CTD-Speed, for example, it means that some compressors are so slow that their compression + transfer + decompression time turns out to be longer than the time required for transferring raw uncompressed data (using a particular link speed, in this case 100 Mbit/sec).

**Figure 2:**
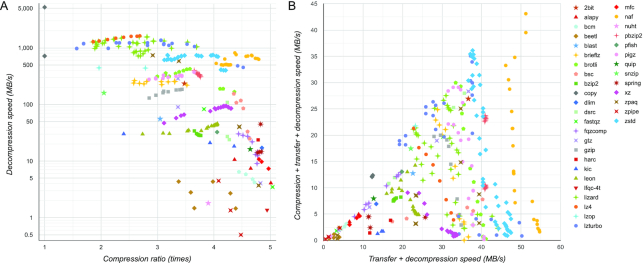
Comparison of 334 settings of 36 compressors on human genome. Each point represents a particular setting of some compressor. A, The relationship between compression ratio and decompression speed. B, The transfer + decompression speed plotted against compression + transfer + decompression speed. Test data are the 3.31 GB reference human genome (accession number GCA_000001405.28). Benchmark CPU: Intel Xeon E5-2643v3 (3.4 GHz). Link speed of 100 Mbit/sec was used for estimating the transfer time.

Fig. 2 compares all compressor settings on the same data (human genome). Fig. 2A shows that the strongest compressors often provide a very low decompression speed (shown using logarithmic scale owing to the enormous range of values), which means that quick data transfer (resulting from strong compression) offered by those compressors is offset by significant waiting time required for decompressing the data. Fig. 2B shows TD-Speed plotted against CTD-Speed. Similar figures can be constructed for other data and performance measures on the SCB database website.

Visualizing results from multiple test datasets simultaneously is possible, with or without aggregation of data. With aggregation, the numbers will be summed or averaged, and a single measurement will be shown for each setting of each compressor. Without aggregation, the results of each compressor setting will be shown separately on each dataset. Because the resulting number of data points can be huge, in such case it is useful to request only the best setting of each compressor to be shown. The criteria for choosing the best setting are selectable among the 17 measurements. In case of a column chart, any of the 17 measures can be used for ordering the compressors shown, independently of the measure used for selecting the best version and independently of the measure actually shown in the chart.

**Figure 3:**
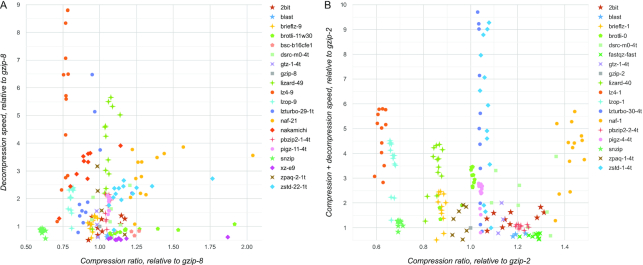
Comparison of compressor settings to gzip. Genome datasets were used as test data. Each point shows the performance of a compressor setting on a specific genome test dataset. All values are shown relative to representative setting of gzip. Only performances that are at least half as good as gzip on both axes are shown. A, Settings that performed best in Transfer + Decompression speed. B, Settings that performed best in Compression + Transfer + Decompression speed. Link speed of 100 Mbit/sec was used for estimating the transfer time.

One useful capability of the SCB database is showing measurements relative to the specified compressor (and setting). This allows a reference compressor to be selected and the other compressors to be compared with this reference. For example, we can compare compressors to gzip as shown in Fig. 3. In this example, we compare only the best settings of each compressor, selected using specific measures (transfer + decompression speed and compression + transfer + decompression speed in Fig. 3A and B, respectively). We also used a fixed scale to show only the range >0.5 on both axes, which means that only performances that are at least half as good as gzip on both axes are shown. In this example, we can see that some compressors improve compactness and some improve speed compared to gzip, but few compressors improve both at the same time, such as lizard, naf, pigz, pbzip, and zstd.

**Figure 4:**
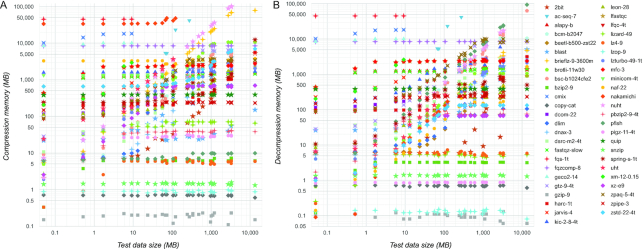
Compressor memory consumption. The strongest setting of each compressor is shown. On the x-axis is the test data size. On the y-axis is the peak memory used by the compressor, for compression (A) and decompression (B).

It is important to be aware of the memory requirements when choosing a compressor (Fig. 4). In these charts we plotted data size on the x-axis, and disabled aggregation. This lets us see how much memory a particular compressor used on each test dataset. As this example shows, memory requirement reaches a saturation point for most compressors. On the other hand, some compressors have unbounded growth of consumed memory, which makes them unusable for large data. Interestingly, gzip apparently has the smallest memory footprint, which may be one of the reasons for its popularity. Most compressors can function on typical desktop hardware, but some require larger memory, which is important to consider when choosing a compressor that will be run by the consumers of distributed data.

A wide variety of charts can be produced on the benchmark website by selecting specific combinations of test data, compressors, and performance measures. At any point the currently visualized data can be obtained in textual form using Table output option. Also, all charts can be downloaded in SVG format.

## Conclusions

Our benchmark reveals a complex relationship between compressors and between their settings, based on various measures. We found that continued use of gzip is usually far from an optimal choice. Transitioning from gzip to a better compressor brings significant gains for genome and protein data and is especially beneficial with repetitive DNA/RNA datasets. The optimal choice of compressor depends on many factors, including properties of the data to be compressed (such as sequence type, data size, and amount of redundancy), relative importance of compression strength, compression speed and decompression speed for particular use scenario, as well as amount of memory available on data machines used for compression and decompression. Our benchmark allows compressors to be compared on individual performance metrics, as well as on their combinations.

The Sequence Compression Benchmark (SCB) database will help in navigating the complex landscape of sequence data compression. With dozens of compressors available, making an informed choice is not an easy task and requires careful analysis of the project requirements, data type, and compressor capabilities. Our benchmark is the first resource providing a detailed practical evaluation of various compressors on a wide range of molecular sequence datasets. Using the SCB database, users can analyze compressor performances on a variety of metrics and construct custom reports for answering project-specific questions.

In contrast to previous studies that showed their results in static tables, our project is dynamic in 2 important senses: (i) the result tables and charts can be dynamically constructed for a custom selection of test data, compressors, and measured performance numbers; and (ii) our study is not a one-off benchmark but marks the start of a project where we will continue to add compressors and test data.

Making an informed choice of a compressor with the help of our benchmark will lead to increased compactness of sequence databases, with shorter time required for downloading and decompressing. This will reduce the load on network and storage infrastructure and increase the speed and efficiency of biological and medical research.

## Methods

### Benchmarked task

The task is to compress and decompress a FASTA-formatted file containing DNA, RNA, or protein sequences. The process has to be lossless, i.e., decompressed data must be byte-to-byte identical to the original data. Compression and decompression are done without using any reference genome. Each compression and decompression task is executed under the Linux OS, via a command line interface. Input data for compression and output data during decompression are streamed using Unix pipes.

Only well-formed FASTA files are used in the benchmark: They must contain no empty lines, and all long sequence lines have to be wrapped at the same position. Both upper- and lower-case (soft-masked) letters can be present, as well as common ambiguity codes. In multiple sequence alignments, additionally, dashes ("-") are used for indicating gaps. Each test dataset is compressed separately from other datasets.

### Compressor selection

We used all specialized sequence compressors that we could find and make to work for the above-specified task. For general-purpose compressors we used only the major ones, in terms of performance, historical importance, or popularity. For each compressor with configurable compression level (or other parameters related to compression strength of speed), we used the relevant range of settings, including the default.

### Benchmark machine

- CPU: dual Xeon E5-2643v3 (3.4 GHz, 6 cores), hyperthreading: off
- RAM: 128 GB DDR4-2133 ECC Registered
- Storage: 4 × 2 TB SSD, in RAID 0, XFS filesystem, block size: 4,096 bytes (blockdev –getbsz)
- OS: Ubuntu 18.04.1 LTS, kernel: 4.15.0
- GCC: 7.4.0

### Compressor/dataset combinations that were tested

Each setting of each compressor was tested on every test dataset, except when it was difficult or impossible owing to compressor limitations:

- AC is a protein-specific compressor and was tested only on protein datasets.
- Owing to their extreme slowness, these compressors were not tested on any data >10 MB: cmix, DNA-COMPACT, GeCo, JARVIS, Leon, and XM.
- UHT failed on the 245 MB dataset and on larger data.
- Nakamichi was only used on data <200 MB owing to its slowness and memory requirements.
- Among sequence compressors, only DELIMINATE, MFCompress, and NAF support multiple sequence alignments.
- Among sequence compressors, only AC, BLAST, and NAF support protein sequences.
- Some settings of XM crashed and/or produced wrong decompressed output on some data—such results are not included.
- NUHT's memory requirement made it impossible to use on the 13.4 GB *Picea abies* genome.
- LFastqC failed on 2.7 GB dataset and larger data.

### Benchmark process

The entire benchmark is orchestrated by a perl script. This script loads the lists of compressor settings and test data, and proceeds to test each combination that still has its measurements missing in the output directory. For each such combination (of compressor setting and test dataset), the following steps are performed:

1. Compression is performed by piping the test data into the compressor. Compressed size and compression time are recorded. For compressed formats consisting of multiple files, sizes of all files are summed together.
2. If compression time did not exceed 10 seconds, 9 more compression runs are performed, recording compression times. Compressed data from previous run are deleted before each subsequent compression run.
3. The next set of compression runs is performed to measure peak memory consumption. This set consists of the same number of runs as in steps 1 and 2 (either 1 or 10 runs). That is, for fast compressors and for small data the measurement is repeated 10 times.
4. Decompression test run is performed. In this run decompressed data are piped to the "md5sum -b -" command. The resulting md5 signature is compared with that of the original file. In case of any mismatch this combination of compressor setting and dataset is disqualified and its measurements are discarded.
5. Decompression time is measured. These time-decompressed data are piped to/dev/null.
6. If decompression completed within 10 seconds, 9 more decompression runs are performed and timed.
7. Peak decompression memory is measured. The number of runs is the same as in steps 5 and 6.
8. The measurements are stored to a file. All compressed and temporary files are removed.

### Measurement methods

Measuring time: wall clock time was measured using Perl's Time::HiRes module (gettimeofday and tv_interval subroutines). The resulting time was recorded with millisecond precision.

Measuring peak memory consumption: first, each compression command was stored in a temporary shell script file. Then it was executed via GNU Time, as/usr/bin/time -v cmd.sh >output.txt. "Maximum resident set size" value was extracted from the output. Then 1,638 was subtracted from this value and the result was stored as peak memory measurement (1,638 is the average "Maximum resident set size" measured by GNU Time in the same way for an empty shell script).

Memory consumption and time were measured separately because measuring memory makes the task slower, especially for very fast tasks.

### Collected measurements

For each combination of compressor and dataset that was tested, the following measurements were collected:

- Compressed size (in bytes)
- Compression time (in milliseconds)
- Decompression time (in milliseconds)
- Peak compression memory (in GNU Time's "Kbytes")
- Peak decompression memory (in GNU Time's "Kbytes")

In cases where 10 values are collected, the average value is used by the benchmark website.

### Computed values

The following values were calculated on the basis of the measured values:

- Compressed size relative to original (%) = Compressed size/uncompressed size * 100
- Compression ratio (times) = Uncompressed size/compressed size
- Compression speed (MB/s) = Uncompressed size in MB/compression time
- Decompression speed (MB/s) = Uncompressed size in MB/decompression time
- Compression + decompression time (s) = compression time + decompression time
- Compression + decompression speed (MB/s) = Uncompressed size in MB/(compression time + decompression time)
- Transfer time (s) = Uncompressed size/Link speed in B/s
- Transfer speed (MB/s) = Uncompressed size in MB/transfer time
- Transfer + decompression time (s) = Transfer time + decompression time
- Transfer + decompression speed (MB/s) = Uncompressed size in MB/(transfer time + decompression time)
- Compression + transfer + decompression time (s) = Compression time + transfer time + decompression time
- Compression + transfer + decompression speed (MB/s) = Uncompressed size in MB/(compression time + transfer time + decompression time)

### Rationale for non-constant number of runs

Variable number of runs is the only way to have both accurate measurements and large test data (under the constraints of using 1 test machine and running benchmark within reasonable time).

On one hand, benchmark takes a lot of time—so much that some compressors cannot be even tested at all on datasets >10 MB in reasonable time. Therefore repeating every measurement 10 times is impractical—or it would imply restricting the test data to only small datasets.

On the other hand, measurements are slightly noisy. The shorter the measured time, the more noisy its measurement. Thus for very quick runs, multiple runs allow for substantial noise suppression. For longer runs it does not make much difference because the relative error is already small with longer times. Using a threshold of 10 seconds seems to be a reasonable compromise between suppressing noise and including larger test data (and slow compressors).

### Streaming mode

For compression, each compressor was reading the input data streamed via unix pipe ("|" in the command line). For decompression, each compressor was set up to stream decompressed data via pipe. This was done to better approximate a common pattern of using compressors in a practical data analysis scenario. In an actual sequence analysis workflow, often decompressed data are piped directly into a downstream analysis command. Also, when compressing the sequences, often the data are first pre-processed with another command, which then pipes processed sequences to a compressor.

Some compressors do not implement the streaming mode, and only work with actual files. Because we have to benchmark all compressors on the same task, we added streaming mode to such compressors via wrapper scripts. For compression, a wrapper reads input data from "stdin" and writes it into a temporary file, then executes a compressor on that file, and finally deletes the file. For decompression the reverse process occurs: The wrapper script executes a decompressor, which writes the decompressed data into a temporary file; then the wrapper reads this file and streams it to "stdout" before deleting the file.

The entire process is timed for the benchmark. Normally such wrapping has minimal impact on the overall compression/decompression speed because we use fast SSD storage and because the actual compression and decompression take comparatively much longer time than simply streaming the data to/from a file.

### FASTA format compatibility

Many specialized compressors do not support the full-featured modern FASTA format, such as the one used in genome databases. Specifically, modern FASTA files often store masked sequence (use a mix of upper- and lower-case letters) and include ambiguity codes. The degree of completeness of FASTA support varies wildly among compressors. At one end of the spectrum, there are compressors with comprehensive support for all FASTA format features. At another end, there are compressors that only work with a string of capital ACGT and nothing else, not even sequence names or newlines. Most sequence compressors fall somewhere between these 2 extremes.

Essentially this means that each sequence compressor performs its own task, different from that of the others. If a compressor does not need to care about upper vs lower-case letters, or about storing sequence names, it can possibly work faster. Thus comparing compressors each doing their own thing would not be fair or very useful to the user. Because full-featured FASTA is in fact commonly used in today's databases, we decided to require complete lossless support of full-featured FASTA files from all benchmarked compressors. In practice this means that we had to create a custom wrapper for each incomplete compressor, implementing the missing compatibility features.

A typical wrapper takes the original FASTA-formatted input and transforms it into a format acceptable by the compressor being wrapped. For instance, if a compressor only expects upper-case nucleotide codes, then the positions of upper- and lower-case characters are extracted and saved in a separate file. The original file is converted to all upper case, which is then fed to the compressor. The separate "mask" file (storing positions of lower-case letters) is compressed with a general-purpose compressor. The entire set of files produced in such a way counts for the compressed data size measured for this particular compressor and dataset, so that the overall compression strength is comparable to that achieved by other compressors (with or without their respective wrappers). Also the total time is measured, including the time taken by all transformations and by storing/compressing the additional files.

We developed several tools for quickly processing FASTA files to extract or add various channels of information for the purpose of wrapping the incomplete compressors. We used C and optimized for speed, so that these steps have maximum speed and minimap impact on the overall compression. The wrapper scripts themselves are written in Perl. We used the fast mode of zstd ("-1") to compress the additional files, chosen because of its high speed so that it has minimal impact on measuring the speed of the wrapped compressor. As for compactness, the impact is minimal as well because the additional files are typically very small and compress well.

For all such wrapped compressors, we benchmarked not only the complete wrapped compressor but also the "wrapper-only" mode, in which only the wrapper script is executed but not the compressor itself. Such results are included in the benchmark under the "wrap-NAME" names. This means that it is possible to compare the speed of the entire wrapped compressor with its corresponding "wrapper-only" run, for each dataset. This allows us to see how much time is used by the wrapper and therefore how much effect the wrapper has on the overall results.

Some of the features implemented via wrappers:

- Supporting RNA sequences for DNA-only compressors
- Supporting "N" in DNA/RNA sequences
- Supporting IUPAC's ambiguous nucleotide codes
- Saving and restoring line lengths
- Saving and restoring sequence names
- Saving and restoring sequence mask (upper/lower case)
- Supporting FASTA-formatted input
- Supporting input with >1 sequence

### FASTQ compressors

Several FASTQ compressors are included in the benchmark. All of them are tested using wrappers that convert FASTA sequences into their respective accepted formats. Some need only the addition of the artificial quality (constant "A" in most cases). Others expect only short reads or reads of identical lengths. These transformations are done in custom wrappers that we made for each FASTQ compressor. Because compression and decompression time recorded for benchmark is the total time of all steps, including wrapper processing, it means that in many cases the wrapped tool may work faster when used directly on FASTQ data. Also many FASTQ compressors are designed under additional assumptions typical for FASTQ data, e.g., that all reads are sampled from an underlying genome with substantial coverage (which allows meaningful assembly). These assumptions often do not hold on our FASTA-formatted benchmark datasets. Therefore the results of FASTQ compressors shown in our benchmark should not be taken as indicative of the actual performance of those compressors on FASTQ data for which they were designed.

### Benchmark code availability

All scripts used for conducting the benchmark are available at the GitHub repository [70]. The main benchmark scripts and configuration files are in the "benchmark" directory. All wrappers are in the "wrappers" directory. Additional tools used by the wrappers are in "seq-tools-c" and "seq-tools-perl" directories. Compression and decompression commands are listed in files "benchmark/compressors-*.txt" and "benchmark/decompressors.txt." Benchmark data are merged using the "benchmark/2-collect-results.pl" script. The resulting merged data are visualized using a server-side script in the "website" directory. The scripts are provided for reference only.

### Update plan

We plan to continue maintaining Sequence Compression Benchmark. This mainly involves benchmarking new or updated compressors when such compressors become available. Because it is impractical to benchmark every existing compressor, we will continue to only benchmark compressors selected on the basis of their performance, quality, and usefulness for sequence compression.

## Availability of Supporting Data and Materials

All benchmark data are available at the online SCB database: http://kirr.dyndns.org/sequence-compression-benchmark/.

An archival copy of benchmark data is also available via the GigaScience database GigaDB [69].

## Availability of Supporting Source Code and Requirements

All code used for conducting the benchmark is available at the SCB GitHub repository [70].

Project name: Sequence Compression Benchmark

Project home page: https://github.com/KirillKryukov/scb

Operating system(s): Linux

Programming language: Perl

Other requirements: None

License: Public Domain

## Additional Files

Supplementary Data contains the raw results of benchmark measurements.

giaa072_GIGA-D-19-00442_Original_Submission

giaa072_GIGA-D-19-00442_Revision_1

giaa072_GIGA-D-19-00442_Revision_2

giaa072_GIGA-D-19-00442_Revision_3

giaa072_Response_to_Reviewer_Comments_Original_Submission

giaa072_Response_to_Reviewer_Comments_Revision_1

giaa072_Response_to_Reviewer_Comments_Revision_2

giaa072_Reviewer_1_Report_Original_Submission Diogo Pratas -- 3/3/2020 Reviewed

giaa072_Reviewer_1_Report_Revision_1 Diogo Pratas -- 6/3/2020 Reviewed

giaa072_Reviewer_2_Report_Original_Submission James Bonfield -- 3/3/2020 Reviewed

giaa072_Supplementary_Data

## Abbreviations

BLAST: Basic Local Alignment Search Tool; CPU: central processing unit; CTD-Speed: compression-transfer-decompression speed; CTD-Time: compression-transfer-decompression time; GB: gigabyte; GCC: GNU Compiler Collection; IUPAC: International Union of Pure and Applied Chemistry; MB: megabyte; NAF: Nucleotide Archival Format; NCBI: National Center for Biotechnology Information; PC: personal computer; OS: operating system; RAM: random access memory; SCB: Sequence Compression Benchmark; SSD: solid-state drive; SVG: Scalable Vector Graphics; TB: terabyte; TD-Speed: transfer-decompression speed; TD-Time: transfer-decompression time; UCSC: University of California Santa Cruz; UHT: unbalanced Huffman tree.

## Competing Interests

The authors declare that they have no competing interests.

## Funding

This work was supported by the 2019 Tokai University School of Medicine Research Aid (to K.K.), JSPS KAKENHI Grants-in-Aid for Scientific Research (C) (20K06612 to K.K.) and Scientific Research on Innovative Areas (16H06429, 16K21723, 19H04843 to S.N.), and Takeda Science Foundation (to T.I.).

## Authors' Contributions

K.K. conceived the study idea and implemented the benchmark. S.N. provided benchmark hardware. K.K., M.T.U., S.N., and T.I. interpreted the data and wrote the manuscript. K.K. and M.T.U. prepared figures and tables. All authors read and approved the final manuscript.
