# Supplementary material for: Sequence Compression Benchmark (SCB) database—A comprehensive evaluation of reference-free compressors for FASTA-formatted sequences
Source: Gigascience. 2020 Jul 6;9(7):giaa072. doi: 10.1093/gigascience/giaa072 (PMC7336184; doi:10.1093/gigascience/giaa072)
Supplement: giaa072_GIGA-D-19-00442_Revision_1 [file giaa072_giga-d-19-00442_revision_1.pdf]

GigaScience

Sequence Compression Benchmark (SCB) database — a comprehensive evaluation of reference-free compressors for FASTA-formatted sequences

--Manuscript Draft--

**Manuscript Number:**	GIGA-D-19-00442R1
**Full Title:**	Sequence Compression Benchmark (SCB) database — a comprehensive evaluation of reference-free compressors for FASTA-formatted sequences
**Article Type:**	Technical Note
**Funding Information:**	School of Medicine, Tokai University — Dr. Kirill Kryukov<br>KAKENHI Grants-in-Aid for Scientific Research on Innovative Areas (16H06429) — Dr. So Nakagawa<br>KAKENHI Grants-in-Aid for Scientific Research on Innovative Areas (16K21723) — Dr. So Nakagawa<br>KAKENHI Grants-in-Aid for Scientific Research on Innovative Areas (19H04843) — Dr. So Nakagawa<br>Takeda Science Foundation — Dr. Tadashi Imanishi<br>KAKENHI Grants-in-Aid for Scientific Research (C) (20K06612) — Dr. Kirill Kryukov
**Abstract:**	Background. Nearly all molecular sequence databases currently use gzip for data compression. Ongoing rapid accumulation of stored data calls for more efficient compression tool. Although numerous compressors exist, both specialized and general-purpose, choosing one of them was difficult because no comprehensive analysis of their comparative advantages for sequence compression was available.<br>Findings. We systematically benchmarked 430 settings of 48 compressors (including 29 specialized sequence compressors and 19 general-purpose compressors) on representative FASTA-formatted datasets of DNA, RNA and protein sequences. Each compressor was evaluated on 17 performance measures, including compression strength, as well as time and memory required for compression and decompression. We used 27 test datasets including individual genomes of various sizes, DNA and RNA datasets, and standard protein datasets. We summarized the results as the Sequence Compression Benchmark database (SCB database, http://kirr.dyndns.org/sequence-compression-benchmark/) that allows building custom visualizations for selected subsets of benchmark results.<br>Conclusion. We found that modern compressors offer large improvement in compactness and speed compared to gzip. Our benchmark allows comparing compressors and their settings using a variety of performance measures, offering the opportunity to select the optimal compressor based on the data type and usage scenario specific to particular application.
**Corresponding Author:**	Kirill Kryukov, Ph.D.<br><br>JAPAN
**Corresponding Author Secondary Information:**	
**Corresponding Author's Institution:**	
**Corresponding Author's Secondary Institution:**	
**First Author:**	Kirill Kryukov, Ph.D.
**First Author Secondary Information:**	
**Order of Authors:**	Kirill Kryukov, Ph.D.<br>Mahoko Takahashi Ueda, Ph.D.<br>So Nakagawa, Ph.D.

Powered by Editorial Manager® and ProduXion Manager® from Aries Systems Corporation

	Tadashi Imanishi, Ph.D.
**Order of Authors Secondary Information:**	
**Response to Reviewers:**	Reply to reviewers<br><br>We sincerely appreciate a thorough review by the two reviewers. Our replies are below.<br><br>> Reviewer #1:<br><br>> This article describes a benchmark for FASTA data that includes online material with a very high potential to be used by the genomic/proteomic data compression community. The benchmark is wide, balanced, and fair. The online tool for visualization of the benchmark is efficiently implemented and easy to follow. The benchmark includes a good set of tools. In general, the work reflects a high knowledge of the tools and bioinformatics background. However, some concerns first need to be addressed before entering in a much more detailed review mode.<br><br>Thank you for taking time to review our work in detail and for the kind comment.<br><br>> Major concerns:<br><br>> There are many compressors for many purposes. Choosing a compressor depends on the purpose. These purposes are not limited to fast decompression of good representations, namely to fast data transfer or integration with other tools. For example, long-term storage removes the importance of fast decompression and increases the importance over the compression ratio. The same can be seen for compressors that aim to approximate the Kolmogorov complexity, namely for genomic or proteomic analysis (phylogenomics, authentication, motif localization, rearrangements, among many others). Here, the importance in only at the efficiency of the compressor side using affordable (usually high) computational time and RAM.<br><br>> Developing efficient genomic/proteomic compressors is also a methodology to improve unsupervised algorithms for data mining or machine learning. An example of this can be seen in the Hutter prize (http://prize.hutter1.net/), a half-million-dollar prize where compressors can spend up to 10GB of RAM and 100 hours to compress 1 GB of data. A version of the PAQ9 algorithm, which is comparatively a very "slow" program, is currently the state-of-the-art. Therefore, centering somewhat the results in NAF (which is perhaps the best industry-oriented FASTA compressor) and limiting the conclusions to the fastest decompression algorithms according to somewhat good compression capabilities does not entirely represent the field of genomic/proteomic data compression. This because FASTA data is already in post-processed state [semi-assembled (contig, scaffold), or assembled], unlike FASTQ. This exclusivity would make sense in FASTQ. Therefore, these notions and wider conclusions would make the manuscript stronger.<br><br>Thank you for your detailed comment. We agree that there are many purposes for compression. This is why our benchmark includes 17 performance measures, including compression ratio. The users of our benchmark are free to consider measures that are most relevant to their application. In the manuscript, we tried to repeatedly emphasize the diversity of applications of our benchmark, because we believe that this benchmark should be useful for a broad variety of compressor uses.<br><br>In our study, we consider an application of compressors for actual data compression, with the main goals of conserving storage, network and computation resources required for managing large amounts of data. We believe that many compressor users (ourselves included) working with large biological datasets may benefit from a detailed investigation of compressor performances, such as what we offer in the current benchmark.<br><br>We do not explicitly address related topics such as "approximating the Kolmogorov complexity", "phylogenomics, authentication, motif localization, rearrangements", "unsupervised algorithms for data mining or machine learning". Involving such topics is currently outside of the scope of our work. We'd like to keep our manuscript focused

and avoid confusing the readers, considering that the issue is already complex, and considering the broad data collected and summarized in our benchmark.

We certainly strongly support scenarios prioritizing compression strength over any other considerations. In fact, majority of the specialized sequence compressors (with few exceptions such as GTZ and DSRC) tend to prioritize compression strength and neglect speed. We have spent substantial efforts and computation resources benchmarking such compressors, because we believe they should be fairly represented, even though we ourselves don't have much use for them.

Regarding the mentioned very slow PAQ9, currently we already include several closely related compressors: cmix (arguably, a state of the art in compression strength for general-purpose data compression), zpaq (descendant from the PAQ family of algorithms), and zpipe (somewhat redundant piping variant of zpaq, although a bit older code). We are open to including more of such compressors in the future. However, long computation time required by some of such compressors means that they may be benchmarked only on smaller datasets, such as in the case of cmix, which is only benchmarked on datasets smaller than 10 MB.

Regarding "centering somewhat the results in NAF". We removed any mentions of NAF from the "Conclusion" section of the manuscript. We still mention NAF along with other top performing compressors in the "Benchmark" section. We are not aware of any of our results that are "centered in NAF".

We believe the revised version of the text is more neutral.

> I also missed some protein sequence compressors, namely the recent protein compressor AC [AC: A Compression Tool for Amino Acid Sequences (https://link.springer.com/article/10.1007/s12539-019-00322-1)]. Sometimes, these are lost in a keyword search. A chain on amino acids can make a protein, therefore, the authors will find protein compressors defined as amino acid sequence compressors. The AC disadvantages: only for protein sequences (not FASTA), slower and, currently, RAM increases according to the redundancy and size of the sequence (but easily it can be adapted to a cache-hash).

Thank you very much for the suggestion. We have added AC to the benchmark.

> Suggestion:

> Given the current times, perhaps a very important dataset to add to the benchmark would be the whole viral database from the NCBI (FASTA format). It can be easily obtained from here: https://www.ncbi.nlm.nih.gov/labs/virus/vssi

Thank you for the suggestion. We have added two datasets from the NCBI Virus datasets that you mention. One is a 122 MB protein dataset "NCBI Virus RefSeq Protein", another is a 482 MB DNA dataset "NCBI Virus Complete Nucleotide Human".

> Minor:

> From 1993 to 2020 there are 27 years, therefore, the longevity of special-purpose compressors is 27-year-old. Biocompress was already available in 1992, before the publication on the DCC (in march of 1993, after review). Therefore, it could also be 28, although 27 is a safe date.

I'm not completely sure where this comment applies, as we don't specifically discuss longevity of special-purpose compressors. However, we mention longevity of gzip, which, coincidentally, was also first released in 1993. As gzip's Wikipedia article (https://en.wikipedia.org/wiki/Gzip) mentions: "Initial release31 October 1992; 27 years ago". Thus, we updated the number to 27.

> Please, improve the format of the figures and tables.

We improved figures and tables (as far as we saw a space for improvement).

> Some of the bullets have a final ".", others don't. Please, pick one and use the same format.

Thank you, we changed the lists to a consistent format. Namely, we use final "." in those bulleted lists where each entry is a complete sentence. In other bulleted lists, where entries are just items of the list, we don't use final ".".

> Reviewer #2:

> General:

> The article is well constructed and has a good explanation of the use cases for different measurement criteria, such as archival, database retrieval, one-time transfers and memory usage.

> There is good attention to detail with specifying the exact versions and commit hashes of each tool, the parameters used (and their processing scripts), and references for downloading each data set. This aids reproducibility and importantly aids the use of this benchmark framework for future software authors.

> Probably this article came out of the analysis for "naf", by the same authors, demonstrating that nibble-packing plus zstd is an unexpectedly strong contender. However a benchmarking framework is a valid and useful piece of work in its own right. To this end, the authors not only provide the results and a useful website, but also the tools used for producing it permitting future tools to be validated against the same data sets using the same methods. This greatly improves the value of this work.

> Specifics:

> 1. The abstract is good. The assertion that most sequence datasets use gzip is valid, if disappointing. I checked the EMBL sequence archive, UniProt/SwissProt and NCBI's RefSeq, all of which are gzipped.

> The findings / conclusion part are also good, stressing the benchmark framework and presentation rather than recommending specific tools which seems appropriate.

> Language throughout is good.

Thank you very much for the time you spent reviewing our work and for encouraging comments.

> 2. The scope needs to be clearly spelt out.

> Specifically it is targeting genomic sequence datasets (eg the aforementioned EMBL sequence databank) and not DNA sequencing reads, hence no quality values either. This is interesting as it's a little bit of a different focus from several other benchmarks.

> It's also excluding reference based compression tools (eg GRS, GReEn, RLZ, CRAM). The line has to be drawn somewhere so I fully understand this, but the scope of what the article covers as well as what it doesn't cover should be more explicit.

Thank you for the suggestion. We have clarified the scope in the "Scope, compressors and test data" section (previously named "Compressors and test data").

> 3. Mentioning "DNA alignments" is a bit ambiguous as most people now think of output from an aligner such as bwa - ie SAM format. The format being used here is the earlier style of dash-padded sequence sets. Please clarify this distinction. I'm not sure what the proper term is, but I think "multiple sequence alignment" covers it.

Thank you for the suggestion. We have changed all mentions of alignment data to use "multiple sequence alignment" wording.

> 4. It is a little unclear precisely which data is being compressed.

> Obviously quality values are not as mention is made to adapting fastq compressors to the task. How about reference names? Other ancillary data after the reference name (oh how I loathe FASTA for that ill-defined mess). Is it purely sequence being evaluated, or the entire FASTA file? Do tools have to be case sensitive? Do they need to cope with ambiguity codes?

> The wrapper scripts cope with some of these things, but it is unclear if this is simply for purposes of testing the compression worked e.g. given the lack of support for lower case, or whether this information is actually being included in the evaluation and added as a side-channel for tools that don't support it natively. If so, how is that done?

> Looking at the wrapper scripts it appears these other types of data get written to separate files and compressed with zstd. This needs documenting in the paper itself, along with an explanation of whether the size of those ancillary files is added to the compressed size, and also whether the time taken is included. (I am assuming yes, but please be explicit.)

Thank you for the suggestion. Indeed this was not clearly explained. We added long explanation in the "Methods" section, under "Streaming mode" and "FASTA format compatibility" headers.

Each compressor has to losslessly compress the entire full-featured FASTA file, including sequence names, case sensitivity and ambiguity codes. All compressors that lack native support for this, receive it via our wrappers. As you correctly assumed, the size of ancillary files, as well as time spent on pre-processing the FASTA stream and extracting these side channels (as well as adding them back during decompression) is counted as part of the total measurement.

Fortunately our wrappers are really fast and don't impact the results much for most compressors. However, all non-trivial wrappers (which means implementing anything more than streaming support) are benchmarked in "wrapper-only" mode and their results are included in benchmark database. Also fortunately those extra files are usually very small and compress well, so they don't impact overall compression rate much.

While admittedly not perfect, this seemed like the only viable strategy that would allow to compare the diverse array of compressors (each doing their own thing), and at the same time to have them doing a useful task (as opposed to compressing a raw stream of ACGT).

> 5. Tool selection.

> There are various fastq compression tools not benchmarked, including but not limited to FQSqueezer, Minicom, Orcom and FaStore. Are these planned? This is hinted at with "our study is not a one-off benchmark, but marks the start of a project where we will continue to add compressors and test data".

> However this is somewhat of a never ending task, as is alluded to with "Since it's impractical to benchmark every existing compressor, we will continue to only benchmark compressors selected based on their performance, quality and usefulness for sequence compression".

> If there are specific reasons why some tools were not evaluated then perhaps this should be mentioned on the website under rejected tools along with a reason (eg for speed, robustness, reordering of data).

Thank you for a good suggestion. Indeed some compressors are still missing in benchmark, each with their own reason. We've been keeping notes about all such potential additions, so it makes perfect sense to share those notes on the website. We now added the "Missing Compressors" page to the website, accessible from the "Compressors" page. Direct link: http://kirr.dyndns.org/sequence-compression-benchmark/?page=Missing-Compressors .

I believe the benchmark is currently reasonably thorough, but there will always be

more compressors to test.

Regarding the mentioned tools:

FQSqueezer - has been added to the benchmark.

Minicom - has been added to the benchmark.

ORCOM - seems to always re-order the reads, making it incompatible with our conditions.

FaStore - seems to always re-order the reads, making it incompatible with our conditions.

I have to add that testing FASTQ compressors on FASTA data (via adding constant quality) is mainly of theoretical interest and probably has little practical value.

FASTQ compressors are usually designed under a FASTQ-specific set of assumptions, such as: "all reads are very short", "all reads are of same length", "order of reads does not matter", "all reads are sampled from underlying genome with substantial coverage". These assumptions don't hold in typical FASTA data and in our benchmark. So the results we obtain for FASTQ compressors may not transfer well to their performance on actual data they are designed for.

We added a mention of this to the "Scope" section on the "About" page on the website.

Still it's interesting to see how different approaches and compressors handle genomes and other FASTA datasets, so we will probably continue to benchmark FASTQ compressors.

> 6. Wrapper scripts/tools.

> How much time is in processing vs the actual tool? For example bsc.pl $cmd is little more than running bsc, while Quip's has 5 components piped together before piping into quip itself. Is the quip tool the bottleneck here and therefore the speed of the other bits irrelevant? I see most are in C, so it's possibly minimal impact, but it is hard to judge. If the impact is minimal, then it's probably best to acknowledge that it was measured and found to be insignificant.

Following this comment, we now also discuss this in the "FASTA format compatibility" part of the "Methods" section. In case when wrappers add anything other than streaming support, we benchmarked the "wrapper-only" runs, so that such runs can be compared with complete "wrapper+compressor" runs. This allows us to see how much of the time is consumed by the wrapper.

In most cases the impact is minimal. There are few cases where wrappers significantly impact compression or decompression speed. Such cases occur when 2 conditions overlap: 1) Compressor is very fast. 2) Compressor requires extensive data preprocessing. Notable examples are 2bit and DSRC.

This can be seen by including both the compressor and its wrapper in a scatterplot produced on benchmark website. We added links to such analyses to the "Examples" page on the website.

> Was CPU (user+system) time measured at all? If so then the ratio of wall clock to CPU time is a good indication of whether the pipeline is causing stalls or not.

Only total wall clock time was measured. I agree this could be interesting, but I don't expect much stalls. Could be interesting to try it some time.

One problem with such measurements is that I found that they influence speed of the fastest compressors. This is why, for example, memory use and speed are measured separately, using different runs of the same compressor.

	> 7. Using fastq-from-sequence may mean that some tools has timings that aren't entirely comparable. While still a valid time for that tool, it's not indicative of the time for the sequence-only portion of that tool.<br><br>Yes, exactly. This is an inevitable consequence of testing FASTQ compressors on FASTA data, and it will remain until we add actual FASTQ data. Currently we are not sure whether we will be able to do it, but it's a possibility we consider for the future.<br><br>> I don't think there is much you can do to mitigate this bar rewriting other peoples code, so realistically it's just something that could be presented as a warning.<br><br>We added an explanation and a warning in the new "FASTQ Compressors" part of the "Methods" section. We also added corresponding warning in the "Scope" section on the "About" page on the website.<br><br>> 8. Be explicit as to the license on your software. Some had a license declaration (public domain) but not all.<br><br>Thanks, we specified a license (public domain) on the "Wrappers" page of the website.<br><br>> 9. A minor typographical: "[A] wide variety of charts can be produced..."<br><br>Thanks, fixed.<br><br>> Thank you for your work.<br><br>We sincerely appreciate your valuable comments on this manuscript.
Additional Information:	
Question	Response
Are you submitting this manuscript to a special series or article collection?	No
**Experimental design and statistics**<br><br>Full details of the experimental design and statistical methods used should be given in the Methods section, as detailed in our Minimum Standards Reporting Checklist. Information essential to interpreting the data presented should be made available in the figure legends.<br><br>Have you included all the information requested in your manuscript?	Yes
**Resources**<br><br>A description of all resources used, including antibodies, cell lines, animals and software tools, with enough information to allow them to be uniquely identified, should be included in the Methods section. Authors are strongly	Yes

Powered by Editorial Manager® and ProduXion Manager® from Aries Systems Corporation

encouraged to cite Research Resource Identifiers (RRIDs) for antibodies, model organisms and tools, where possible.<br><br>Have you included the information requested as detailed in our Minimum Standards Reporting Checklist?	
**Availability of data and materials**<br><br>All datasets and code on which the conclusions of the paper rely must be either included in your submission or deposited in publicly available repositories (where available and ethically appropriate), referencing such data using a unique identifier in the references and in the “Availability of Data and Materials” section of your manuscript.<br><br>Have you have met the above requirement as detailed in our Minimum Standards Reporting Checklist?	Yes

Manuscript Click here to access/download;Manuscript;Text.pdf

Sequence Compression Benchmark (SCB) database — a comprehensive evaluation of reference-free compressors for FASTA-formatted sequences

Kirill Kryukov*, Mahoko Takahashi Ueda, So Nakagawa, Tadashi Imanishi

Department of Molecular Life Science, Tokai University School of Medicine,

Isehara, Kanagawa 259-1193, Japan.

*Correspondence: kkryukov@gmail.com

Abstract

Background. Nearly all molecular sequence databases currently use gzip for data compression. Ongoing rapid accumulation of stored data calls for more efficient compression tool. Although numerous compressors exist, both specialized and general-purpose, choosing one of them was difficult because no comprehensive analysis of their comparative advantages for sequence compression was available.

Findings. We systematically benchmarked 430 settings of 48 compressors (including 29 specialized sequence compressors and 19 general-purpose compressors) on representative FASTA-formatted datasets of DNA, RNA and protein sequences. Each compressor was evaluated on 17 performance measures, including compression strength, as well as time and memory required for compression and decompression. We used 27 test datasets including individual genomes of various sizes, DNA and RNA datasets, and standard protein datasets. We summarized the results as the Sequence Compression Benchmark database (SCB database, http://kirr.dyndns.org/sequence-compression-benchmark/) that allows building custom visualizations for selected subsets of benchmark results.

Conclusion. We found that modern compressors offer large improvement in compactness and speed compared to gzip. Our benchmark allows comparing compressors and their settings using a variety of performance measures, offering the opportunity to select the optimal compressor based on the data type and usage scenario specific to particular application.

Keywords: compression; benchmark; DNA; RNA; protein; genome; sequence; database.

Background

Molecular sequence databases store and distribute DNA, RNA and protein sequences as compressed FASTA-formatted files. Biological sequence compression was first proposed in 1986 [1] and the first practical compressor was made in 1993 [2]. A lively field emerged that produced a stream of methods, algorithms, and software tools for sequence compression [3,4]. However, despite this activity, currently nearly all databases universally depend on gzip for compressing FASTA-formatted sequence data. This incredible longevity of the 27-year-old compressor probably owes to multiple factors, including conservatism of database operators, wide availability of gzip, and its generally acceptable performance. Through all these years the amount of stored sequence data kept growing steadily [5], increasing the load on database operators, users, storage systems and network infrastructure. However, someone thinking to replace gzip invariably faces the questions: which of the numerous available compressors to choose? And will the resulting gains be even worth the trouble of switching?

Previous attempts at answering these questions are limited by testing too few compressors and by using restricted test data [6-11]. In addition, all of these studies provide results in form of tables, with no graphical outputs, which makes the interpretation difficult. Existing benchmarks with useful visualization such as Squash [12], are limited to general-purpose compressors.

The variety of available specialized and general-purpose compressors is overwhelming. At the same time the field was lacking a thorough investigation of comparative merits of these compressors for sequence data. Therefore we set out to benchmark all available and practically useful compressors on a variety of relevant sequence data. Specifically, we focused on the common task of compressing DNA, RNA and protein sequences, stored in FASTA format, without using reference sequence. The benchmark results were shown in the Sequence Compression Benchmark database (SCB database, http://kirr.dyndns.org/sequence-compression-benchmark/).

Scope, compressors and test data

We considered the common scenario of archiving, transferring and working with large datasets of biological sequences. In this study we did not investigate compression of raw sequencing data in FASTQ

format, which was previously thorougly reviewed in [11]. Instead we focused on typical FASTA-formatted datasets, which includes individual genomes and single gene sets. Consequently we also did not consider referential compression, but only reference-free compression, which is typically used for such data. We evaluated standalone compression tools (rather than libraries), working under Linux OS on a modern workstation PC. In this study we only consider lossless compression.

We tested all DNA sequence compressors that are available and functional in 2020: dnaX [13], XM [14], DELIMINATE [15], Pufferfish [16], DNA-COMPACT [17], MFCompress [18], UHT [19], GeCo [20], GeCo2 [21], JARVIS [22], NAF [23], and NUHT [24]. We also included the relatively compact among homology search database formats: BLAST [25] and 2bit - a database format of BLAT [26].

Since compressors designed for FASTQ data can be trivially adopted for FASTA-formatted inputs, we also included a comprehensive array of compressors designed primarily or specifically for FASTQ data: BEETL [27], Quip [28], fastqz [10], fqzcomp [10], DSRC 2 [29], Leon [30], LFQC [31], KIC [32], ALAPY [33], GTX.Zip [34], HARC [35], LFastqC [36], SPRING [37], Minicom [38], and FQSqueezer [39]. We also included AC - a compressor designed exclusively for protein sequences [40]. We also tested a comprehensive array of general purpose compressors: bcm [41], brieflz[42], brotli [43], bsc [44], bzip2 [45], cmix [46], gzip [47], lizard [48], lz4 [49], lzop [50], lzturbo [51], nakamichi [52], pbzip2 [53], pigz [54], snzip [55], xz [56], zpaq [57], zpipe [57] and zstd [58]. See Table 1 for the list of compressors we used.

For the test data, we selected a variety of commonly used sequence datasets in FASTA format: (1) Individual genomes of various sizes, as examples of non-repetitive data [59,60]; (2) DNA and RNA datasets, such as collections of mitochondrial genomes, influenza virus sequences [60,61,62,59], 16S rRNA gene sequences [63], and genomic multiple DNA sequence alignments [64]; (3) Standard protein datasets [65,66,61,67]. Individual genomes are less repetitive, while other datasets are more repetitive. In total we used 27 test datasets. See Table 2 for the list of test data. All test data is available at the GigaDB repository.

Benchmark

We benchmarked each compressor on every test dataset, except in cases of incompatibility (e.g., DNA compressors cannot compress protein data) or excessive time requirement (some compressors are so slow that they would take weeks on larger datasets). For compressors with adjustable compression level, we

tested the relevant range of levels. We tested both 1 and 4-thread variants of compressors that support multi-threading. In total, we used 430 settings of 48 compressors. We also included the non-compressing "cat" command as control. For compressors using wrappers, we also benchmarked the wrappers.

Currently many sequence analysis tools support gzip-compressed files as input. Switching to another compressor may require either adding support of new format to those tools, or passing the data in uncompressed form. The latter solution can be achieved with the help of Unix pipes, if both the compressor and the analysis tool support streaming mode. Therefore, we benchmarked all compressors in streaming mode (streaming uncompressed data in both compression and decompression).

For each combination of compressor setting and test dataset we recorded compressed size, compression time, decompression time, peak compression memory and peak decompression memory. The details of the method and raw benchmark data are available in the Methods section and Supplementary Data, respectively. We share benchmark results and scripts at the SCM database website: http://kirr.dyndns.org/sequence-compression-benchmark/.

The choice of measure for evaluating compressor performance depends on prospective application. For long-term data storage, compactness may be the single most important criterion. For public sequence database, the key measure is how long time it takes from initiating the download of compressed files until accessing the decompressed data. This time consists of transfer time plus decompression time (TD-Time). Corresponding transfer-decompression speed (TD-Speed) is computed as Original Size / TD-Time. In this use case compression time is relatively unimportant, since compression happens only once, while transfer and decompression times affect every user of the database. For one-time data transfer, all three steps of compression, transfer and decompression are timed (CTD-Time), and used for computing the resulting overall speed (CTD-Speed).

A total of 17 measures, including the above-mentioned ones, are available in our results data (See Methods for the list of measures). Any of these measures can be used for selecting the best setting of each compressor and for sorting the list of compressors. These measures can be then shown in a table and visualized in column charts and scatterplots. This allows tailoring the output to answer specific questions, such as what compressor is better at compressing particular kind of data, or which setting of each compressor

performs best at particular task. The link speed that is used for estimating transfer times is configurable. The default speed of 100 Mbit/sec corresponds to the common speed of a fixed broadband internet connection.

Fig.1 compares the performance of best settings of 36 compressors on human genome. It shows that specialized sequence compressors achieve excellent compression ratio on this genome. However, when total TD-Speed or CTD-Speed is considered (measures that are important in practical applications), most sequence compressors fall behind the general-purpose ones. The best compressors for this dataset in terms of compression ratio, TD-Speed and CTD-Speed are "fastqz-slow", "naf-22" and "naf-1", respectively (numbers in each compressor name indicate compression level and other settings). Interestingly, the non-compressing "cat" command used as control, while naturally showing at the last place on compression ratio (Fig.1A), is not the slowest in terms of TD-Speed and CTD-Speed (Figs.1B and 1C, respectively). In case of CTD-Speed, for example, it means that some compressors are so slow that their compression + transfer + decompression time turns out to be longer than time required for transferring raw uncompressed data (using particular link speed, in this case 100 Mbit/sec).

Fig.2 compares all compressor settings on the same data (human genome). Fig.2A shows that the strongest compressors often provide very low decompression speed (shown using logarithmic scale due to the enormous range of values), which means that quick data transfer (resulting from strong compression) offered by those compressors is offset by significant waiting time required for decompressing the data. Fig.2B shows TD-Speed plotted against CTD-Speed. Similar figures can be constructed for other data and performance measures on the SCB database website.

Visualizing results from multiple test datasets simultaneously is possible, with or without aggregation of data. With aggregation, the numbers will be summed or averaged, and a single measurement will be shown for each setting of each compressor. Without aggregation, the results of each compressor setting will be shown separately on each dataset. Since the resulting number of data points can be huge, in such case it is useful to request only best settings of each compressor to be shown. The criteria for choosing the best setting is selectable among the 17 measurements. In case of a column chart, any of the 17 measures can be used for ordering the shown compressors, independently of the setting used for selecting best version, and independently for the measure actually shown in the chart.

One useful capability of the SCB database is showing measurements relative to specified compressor (and setting). This allows selecting a reference compressor and comparing the other compressors to this reference. For example, we can compare compressors to gzip as shown on Fig.3. In this example, we compare only best settings of each compressor, selected using specific measures (transfer+decompression speed and compression+transfer+decompression speed on Figs.3A and 3B, respectively). We also used fixed scale to show only range above 0.5 on both axes, which means that only performances that are at least half as good as gzip on both axes as shown. In this example, we can see that some compressors improve compactness and some improve speed compared to gzip, but few compressors improve both at the same time, such as lizard, naf, pigz, pbzip, and zstd.

It is important to be aware of the memory requirements when choosing a compressor (Fig.4). In these charts we plotted data size on the X axis, and disabled aggregation. This allows seeing how much memory a particular compressor used on each test dataset. As this example shows memory requirement reaches saturation point for most compressors. On the other hand, some compressors have unbounded growth of consumed memory, which makes then unusable for large data. Interestingly, gzip apparently has the smallest memory footprint, which may be one of the reasons for its popularity. Most compressors can function on a typical desktop hardware, but some require larger memory, which is important to consider when choosing a compressor that will be run by the consumers of distributed data.

A wide variety of charts can be produced on the benchmark website by selecting specific combinations of test data, compressors, and performance measures. At any point the currently visualized data can be obtained in textual form using Table output option. Also, all charts can be downloaded in SVG format.

Conclusions

Our benchmark reveals complex relationship between compressors and between their settings, based on various measures. We found that continued use of gzip is usually far from an optimal choice. Transitioning from gzip to a better compressor brings significant gains for genome and protein data, and is especially beneficial with repetitive DNA/RNA datasets. The optimal choice of compressor depends on many factors, including properties of the data to be compressed (such as sequence type, data size, and

amount of redundancy), relative importance of compression strength, compression speed and decompression speed for particular use scenario, as well as amount of memory available on data machines used for compression and decompression. Our benchmark allows comparing compressors on individual performance metrics, as well as on their combinations.

The Sequence Compression Benchmark (SCB) database will help in navigating the complex landscape of data compression. With dozens of compressors available, making an informed choice is not an easy task and requires careful analysis of the project requirements, data type and compressor capabilities. Our benchmark is the first resource providing a detailed practical evaluation of various compressors on a wide range of molecular sequence datasets. Using the SCB database, users can analyze compressor performances on variety of metrics, and construct custom reports for answering project-specific questions.

In contrast to previous studies that showed their results in static tables, our project is dynamic in two important senses: (1) the result tables and charts can be dynamically constructed for a custom selection of test data, compressors, and measured performance numbers, and (2) our study is not a one-off benchmark, but marks the start of a project where we will continue to add compressors and test data.

Making an informed choice of compressor with the help of our benchmark will lead to increased compactness of sequence databases, with shorter time required for downloading and decompressing. This will reduce the load on network and storage infrastructure, and increase speed and efficiency in biological and medical research.

Declarations

Availability of data and material

All benchmark data is available at the online SCB database:
http://kirr.dyndns.org/sequence-compression-benchmark/

Competing interests

The authors declare no competing interests.

Funding

This work was supported by the 2019 Tokai University School of Medicine Research Aid (to KK), JSPS KAKENHI Grants-in-Aid for Scientific Research (C) (20K06612 to KK) and Scientific Research on Innovative Areas (16H06429, 16K21723, 19H04843 to SN), and Takeda Science Foundation (to TI).

Authors' contributions

KK conceived the study idea and implemented the benchmark. SN provided benchmark hardware. KK, MTU, SN and TI interpreted the data and wrote the manuscript. KK and MTU prepared figures and tables. All authors read and approved the final manuscript.

Acknowledgements

Not applicable.

References

1. Walker JR, Willett P. Compression of nucleic acid and protein sequence data. *Comput. Appl. Biosci.* 1986;2(2):89-93.
2. Grumbach S, Tahi F. Compression of DNA sequences. *Data Compression Conference*, Snowbird, Utah, IEEE Computer Society. 1993. p. 340-50. doi:10.1109/DCC.1993.253115.
3. Deorowicz S, Grabowski S. Data compression for sequencing data. *Algorithms for Molecular Biology*. 2013;8:25. doi:10.1186/1748-7188-8-25.
4. Hernaez M, Pavlichin D, Weissman T, Ochoa I. Genomic Data Compression. *Annual Review of Biomedical Data Science*. 2019;2:19-37. doi:10.1146/annurev-biodatasci-072018-021229.
5. Karsch-Mizrachi I, Takagi T, Cochrane G. The international nucleotide sequence database collaboration. *Nucleic Acids Res.* 2018;46(Database issue):D48–D51. doi:10.1093/nar/gkx1097.
6. Zhu Z, Zhang Y, Ji Z, He S, Yang X. High-throughput DNA sequence data compression. *Brief. Bioinform.* 2013; 16(1):1-15. doi:10.1093/bib/bbt087.
7. Hosseini M, Pratas D, Pinho AJ. A Survey on Data Compression Methods for Biological Sequences. *Information*. 2016;7(4):56. doi:10.3390/info7040056.
8. Sardaraz M, Tahir M. Advances in high throughput DNA sequence data compression. *J. Bioinform. Comput. Biol*. 2016;14(3):1630002. doi:10.1142/S0219720016300021.

9. Biji CL, Achuthsankar SN. Benchmark Dataset for Whole Genome Sequence Compression. *IEEE/ACM Trans. Comput. Biol. Bioinform.* 2017;14(6):1228-36. doi:10.1109/TCBB.2016.2568186.
10. Bonfield JK, Mahoney MV. Compression of FASTQ and SAM Format Sequencing Data. *PLoS One*. 2013;8(3): e59190, doi:10.1371/journal.pone.0059190.
11. Numanagic I, Bonfield JK, Hach F, Voges J, Ostermann J, Alberti C, Mattavelli M. Comparison of high-throughput sequencing data compression tools. *Nature Methods*. 2016;13(12):1005-8, doi:10.1038/nmeth.4037.
12. Squash Compression Benchmark. 2015. https://quixdb.github.io/squash-benchmark/. Accessed July 15, 2019.
13. Manzini G, Rastero M. A simple and fast DNA compressor. *Software - Practice and Experience.* 2004;34:1397-411, doi:10.1002/spe.619.
14. Cao MD, Dix TI, Allison L. Mears C. A simple statistical algorithm for biological sequence compression. *Data Compression Conference*. DCC '07, Snowbird, UT, IEEE Computer Society. 2007. p. 43-52. doi:10.1109/DCC.2007.7.
15. Mohammed MH, Dutta A, Bose T, Chadaram S, Mande SS. DELIMINATE — a fast and efficient method for loss-less compression of genomic sequences. *Bioinformatics*. 2012;28:2527–29. doi:10.1093/bioinformatics/bts467.
16. Pufferfish. 2012. https://github.com/alexholehouse/pufferfish. Accessed May 23, 2019.
17. Li P, Wang S, Kim J, Xiong H, Ohno-Machado L, Jiang X. DNA-COMPACT: DNA COMpression Based on a Pattern-Aware Contextual Modeling Technique. *PLoS ONE.* 2013;8(11):e80377. doi:10.1371/journal.pone.0080377.
18. Pinho AJ, Pratas D. MFCompress: a compression tool for FASTA and multi-FASTA data. *Bioinformatics*. 2014;30:117-8. doi:10.1093/bioinformatics/btt594.
19. Al-Okaily A, Almarri B, Al Yami S, Huang CH. Toward a Better Compression for DNA Sequences Using Huffman Encoding. *J. Comp. Biol.* 2017;24(4):280–8. doi:10.1089/cmb.2016.0151.
20. Pratas D, Pinho AJ, Ferreira PJSG. Efficient compression of genomic sequences. *Data Compression Conference*, DCC-2016, Snowbird, Utah, IEEE Computer Society. 2016. p.231-240. doi: 10.1109/DCC.2016.60.

21. Pratas D, Hosseini M, Pinho AJ. GeCo2: An Optimized Tool for Lossless Compression and Analysis of DNA Sequences. *Practical Applications of Computational Biology and Bioinformatics, 13th International Conference*, PACBB 2019, Advances in Intelligent Systems and Computing, vol 1005, Springer, Cham, 2019a. p.137-145. doi: 10.1007/978-3-030-23873-5_17.
22. Pratas D, Hosseini M, Silva J, Pinho AJ. A Reference-Free Lossless Compression Algorithm for DNA Sequences Using a Competitive Prediction of Two Classes of Weighted Models. *Entropy*, 2019b;21:1074. doi:10.3390/e21111074.
23. Kryukov K, Ueda MT, Nakagawa S, Imanishi T. Nucleotide Archival Format (NAF) enables efficient lossless reference-free compression of DNA sequences. *Bioinformatics.* 2019;35(19):3826-28. doi:10.1093/bioinformatics/btz144.
24. Alyami S, Huang CH. Nongreedy Unbalanced Huffman Tree Compressor for Single and Multifasta Files. Journal of Computational Biology. 2019; 26(0):1-9. doi:10.1089/cmb.2019.0249.
25. Altschul SF, Gish W, Miller W, Myers EW, Lipman DJ. Basic local alignment search tool. *J. Mol. Biol.* 1990;215(3):403-10. doi:10.1016/S0022-2836(05)80360-2.
26. Kent WJ. BLAT - The BLAST-Like Alignment Tool. *Genome Research.* 2002;12(4):656-64. doi:10.1101/gr.229202.
27. Bauer MJ, Cox AJ, Rosone G. Lightweight BWT Construction for Very Large String Collections. *Combinatorial Pattern Matching 2011*, proceedings of the CPM 2011, 2011. p.219-231. doi:10.1007/978-3-642-21458-5_20.
28. Jones DC, Ruzzo WL, Peng X, Katze MG. Compression of next-generation sequencing reads aided by highly efficient de novo assembly. *Nucleic Acids Research.* 2012;40(22):e171. doi:10.1093/nar/gks754.
29. Roguski L, Deorowicz S. DSRC 2—Industry-oriented compression of FASTQ files. *Bioinformatics.* 2014; 30(15):2213-5. doi:10.1093/bioinformatics/btu208.
30. Benoit G, Lemaitre C, Lavenier D, Drezen E, Dayris T, Uricaru R, Rizk G. Reference-free compression of high throughput sequencing data with a probabilistic de Bruijn graph. *BMC Bioinformatics*. 2015;16:288. doi:10.1186/s12859-015-0709-7.
31. Nicolae M, Pathak S, Rajasekaran S. LFQC: a lossless compression algorithm for FASTQ files. *Bioinformatics.* 2015;31(20):3276-81. doi:10.1093/bioinformatics/btv384.

32. Zhang Y, Patel K, Endrawis T, Bowers A, Sun Y. A FASTQ compressor based on integer-mapped k-mer indexing for biologist. *Gene.* 2016;579(1):75-81. doi:10.1016/j.gene.2015.12.053.
33. ALAPY 2017. http://alapy.com/services/alapy-compressor/. Accessed December 2, 2019.
34. Xing Y, Li G, Wang Z, Feng B, Song Z, Wu C. GTZ: a fast compression and cloud transmission tool optimized for FASTQ files. *BMC Bioinformatics.* 2017;18(Suppl 16):549. doi:10.1186/s12859-017-1973-5.
35. Chandak S, Tatwawadi K, Weissman T. Compression of genomic sequencing reads via hash-based reordering: algorithm and analysis. *Bioinformatics.* 2018;34(4):558-67. doi:10.1093/bioinformatics/btx639.
36. Al Yami S, Huang CH. LFastqC: A lossless non-reference-based FASTQ compressor. PLoS One. 2019;14(11):e0224806, doi:10.1371/journal.pone.0224806.
37. Chandak S, Tatwawadi K, Ochoa I, Hernaez M, Weissman T. SPRING: a next-generation compressor for FASTQ data. Bioinformatics. 2019;35(15):2674-6. doi:10.1093/bioinformatics/bty1015.
38. Liu Y, Yu Z, Dinger ME, Li J. Index suffix-prefix overlaps by (w, k)-minimizer to generate long contigs for reads compression. Bioinformatics. 2019. 35(12):2066-2074, doi:10.1093/bioinformatics/bty936.
39. Deorowicz S. FQSqueezer: k-mer-based compression of sequencing data. Scientific Reports. 2020;10:578. doi:10.1038/s41598-020-57452-6.
40. Hosseini M, Pratas D, Pinho AJ. AC: A Compression Tool for Amino Acid Sequences. Interdisciplinary Sciences: Computational Life Sciences. 2019;11:68-76. doi:10.1007/s12539-019-00322-1.
41. BCM. https://github.com/encode84/bcm. Accessed June 6 2019.
42. BriefLZ - small fast Lempel-Ziv. https://github.com/jibsen/brieflz. Accessed May 12 2020.
43. Alakuijala J, Szabadka Z. Brotli Compressed Data Format. RFC 7932. 2016. Accessed April 14 2019.
44. libbsc. https://github.com/IlyaGrebnov/libbsc. Accessed June 22 2019.
45. bzip2. https://www.sourceware.org/bzip2/. Accessed January 20 2019.
46. cmix. https://github.com/byronknoll/cmix. Accessed April 25 2019.
47. GNU Gzip. https://www.gnu.org/software/gzip/. Accessed November 8 2019.
48. Lizard - efficient compression with very fast decompression. https://github.com/inikep/lizard. Accessed June 16 2019.

49. LZ4 - Extremely fast compression. https://github.com/lz4/lz4. Accessed April 25 2019.
50. Lzop. 2017. https://www.lzop.org/. Accessed December 6 2018.
51. LzTurbo - World's fastest compressor. https://sites.google.com/site/powturbo/. Accessed February 11 2019.
52. Nakamichi. http://www.sanmayce.com/Nakamichi/index.html. Accessed May 12 2020.
53. pbzip2. https://launchpad.net/pbzip2/. Accessed April 26 2019.
54. pigz. https://zlib.net/pigz/. Accessed April 26 2019.
55. Snzip, a compression/decompression tool based on snappy. https://github.com/kubo/snzip. Accessed November 11 2018.
56. XZ Utils. https://tukaani.org/xz/. Accessed December 17 2018.
57. ZPAQ Incremental Journaling Backup Utility and Archiver. http://www.mattmahoney.net/dc/zpaq.html. Accessed November 7 2018.
58. Zstandard - Fast real-time compression algorithm. https://github.com/facebook/zstd. Accessed May 22 2020.
59. Clark K, Karsch-Mizrachi I, Lipman DJ, Ostell J, Sayers EW. GenBank. *Nucleic Acids Res.* 2016;44(D1):D67–D72. doi:10.1093/nar/gkv1276.
60. O'Leary NA, Wright MW, Brister JR, Ciufo S, Haddad D, McVeigh R, et al. Reference sequence (RefSeq) database at NCBI: current status, taxonomic expansion, and functional annotation. *Nucleic Acids Res.* 2016;44(D1):D733-45. doi:10.1093/nar/gkv1189.
61. Brister JR, Ako-Adjei D, Bao Y, Blinkova O. NCBI viral genomes resource. *Nucleic Acids Res.* 2015;43(D1):D571-7. doi:10.1093/nar/gku1207.
62. Bao Y, Bolotov P, Dernovoy D, Kiryutin B, Zaslavsky L, Tatusova T, Ostell J, Lipman D. The Influenza Virus Resource at the National Center for Biotechnology Information. *J Virol.* 2008;82(2):596-601. doi:10.1128/JVI.02005-07.
63. Quast C, Pruesse E, Yilmaz P, Gerken J, Schweer T, Yarza P, Peplies J, Glöckner FO. The SILVA ribosomal RNA gene database project: improved data processing and web-based tools. *Nucl. Acids Res.* 2013;41(D1):D590-D596. doi:10.1093/nar/gks1219.

64. Kent WJ, Sugnet CW, Furey TS, Roskin KM, Pringle TH, Zahler AM, Haussler D. The human genome browser at UCSC. *Genome Res.* 2002;12(6):996-1006. doi:10.1101/gr.229102.

65. Berman HM, Westbrook J, Feng Z, Gilliland G, Bhat TN, Weissig H, Shindyalov IN, Bourne PE. The Protein Data Bank. *Nucleic Acids Res.* 2000;28:235-42. doi:10.1093/nar/28.1.235.

66. Yates AD, Achuthan P, Akanni W, Allen J, Allen J, Alvarez-Jarreta J, et al. Ensembl 2020. *Nucleic Acids Res.* 2020;48(D1):D682–8. doi:10.1093/nar/gkz966.

67. The UniProt Consortium. UniProt: a worldwide hub of protein knowledge. *Nucleic Acids Res.* 2019;47(D1):D506-15. doi:10.1093/nar/gky1049.

Methods

Benchmarked task

The task is to compress and decompress a FASTA-formatted file containing DNA, RNA or protein sequences. The process has to be lossless, i.e., decompressed data must be byte-to-byte identical to the original data. Compression and decompression are done without using any reference genome. Each compression and decompression task is executed via a Linux in a command line interface. Input data for compression and output data during decompression are streamed using Unix pipes.

Only well-formed FASTA files are used in the benchmark: They must contain no empty lines and all long sequence lines have to be wrapped at the same position. Both large and small (soft-masked) letters can be present, as well as common ambiguity codes. In multiple sequence alignments, additionally, dash ("-") is used for indicating gaps. Each test dataset is compressed separately from other datasets.

Compressor selection

We used all specialized sequence compressors that we could find and make to work for the above specified task. For general-purpose compressors we used only the major ones, in terms of performance, historical importance, or popularity. For each compressor with configurable compression level (or other parameters related to compression strength of speed), we used the relevant range of settings, including the default.

Benchmark machine

- CPU: dual Xeon E5-2643v3 (3.4 GHz, 6 cores), hyperthreading: off
- RAM: 128 GB DDR4-2133 ECC Registered
- Storage: 4 x 2 TB SSD, in RAID 0, XFS filesystem, block size: 4096 bytes (blockdev --getbsz)
- OS: Ubuntu 18.04.1 LTS, kernel: 4.15.0
- GCC: 7.4.0

Compressor/dataset combinations that were tested

Each setting of each compressor is tested on every test dataset, except when it's difficult or impossible due to compressor limitations:

- AC is a protein-specific compressor, and was tested only on protein datasets.
- Due to their extreme slowness, these compressors are not tested on any data larger than 10 MB: cmix, DNA-COMPACT, GeCo, JARVIS, Leon, and XM.
- UHT fails on the 245 MB dataset and on larger data.
- Nakamichi was only used on data smaller than 200 MB due to its slowness and memory requirements.
- Among sequence compressors, only DELIMINATE, MFCompress and NAF support multiple sequence alignments.
- Among sequence compressors, only AC, BLAST and NAF support protein sequences.
- Some settings of XM crash and/or produce wrong decompressed output on some data - such results are not included.
- NUHT's memory requirement makes it impossible to use on 13.4 GB *Picea abies* genome.
- LFastqC fails on 2.7 GB dataset and larger data.

Benchmark process

The entire benchmark is orchestrated by a perl script. This script loads the lists of compressor settings and test data, and proceeds to test each combination that still has its measurements missing in the output directory. For each such combination (of compressor setting and test dataset), the following steps are performed:

1. Compression is performed by piping the test data into the compressor. Compressed size and compression time is recorded. For compressed formats consisting of multiple files, sizes of all files are summed together.
2. If compression time did not exceed 10 seconds, 9 more compression runs are performed, recording compression times. Compressed data from previous run is deleted before each next compression run.
3. The next set of compression runs is performed to measure peak memory consumption. This set consists of the same number of runs as in steps 1-2 (either 1 or 10 runs). That is, for fast compressors and for small data the measurement is repeated 10 times.
4. Decompression test run is performed. In this run decompressed data is piped to the "md5sum -b -" command. The resulting md5 signature is compared with that of the original file. In case of any mismatch this combination of compressor setting and dataset is disqualified and its measurements are discarded.
5. Decompression time is measured. This time decompressed data is piped to /dev/null.
6. If decompression completed within 10 seconds, 9 more decompression runs are performed and timed.
7. Peak decompression memory is measured. The number of runs is same as in steps 5-6.
8. The measurements are stored to a file. All compressed and temporary files are removed.

Measurement methods

Measuring time: Wall clock time was measured using Perl's Time::HiRes module (gettimeofday and tv_interval subroutines). The resulting time was recorded with millisecond precision.

Measuring peak memory consumption: First, each compression command was stored in a temporary shell script file. Then it was executed via GNU Time, as /usr/bin/time -v cmd.sh >output.txt. "Maximum resident set size" value was extracted from the output. 1638 was then subtracted from this value and the result was stored as peak memory measurement. 1638 is the average "Maximum resident set size" measured by GNU Time in the same way for an empty shell script.

Memory consumption and time were measured separately because measuring memory makes the task slower, especially for very fast tasks.

Collected measurements

For each combination of compressor and dataset that was tested, the following measurements were collected:

- Compressed size (in bytes)
- Compression time (in milliseconds)
- Decompression time (in milliseconds)
- Peak compression memory (in GNU Time's "Kbytes")
- Peak decompression memory (in GNU Time's "Kbytes")

In cases where 10 values are collected, the average value is used by the benchmark web-site.

Computed values

The following values were calculated based on the measured values:

- Compressed size relative to original (%) = Compressed size / Uncompressed size * 100
- Compression ratio (times) = Uncompressed size / Compressed size
- Compression speed (MB/s) = Uncompressed size in MB / Compression time
- Decompression speed (MB/s) = Uncompressed size in MB / Decompression time
- Compression + decompression time (s) = Compression time + Decompression time
- Compression + decompression speed (MB/s) = Uncompressed size in MB / (Compression time + Decompression time)
- Transfer time (s) = Uncompressed size / Link speed in B/s
- Transfer speed (MB/s) = Uncompressed size in MB / Transfer time
- Transfer + decompression time (s) = Transfer time + Decompression time
- Transfer + decompression speed (MB/s) = Uncompressed size in MB / (Transfer time + Decompression time)
- Compression + transfer + decompression time (s) = Compression time + Transfer time + Decompression time
- Compression + transfer + decompression speed (MB/s) = Uncompressed size in MB / (Compression time + Transfer time + Decompression time)

Rationale for non-constant number of runs

Variable number of runs is the only way to have both accurate measurements and large test data (under the constraints of using one test machine, and running benchmark within reasonable time).

On one hand, benchmark takes lot of time. So much that some compressors can't be even tested at all on dataset larger than 10 MB in reasonable time. Therefore repeating every measurement 10 times is impractical. Or, it would imply restricting the test data to only small datasets.

On the other hand, measurements are slightly noisy. The shorter measured time, the more noisy its measurement. Thus for very quick runs, multiple runs allow for substantial noise suppression. For longer runs it does not make much difference, because the relative error is already small with longer times.

Using a threshold of 10 seconds seems to be a reasonable compromise between suppressing noise and including larger test data (and slow compressors).

Streaming mode

For compression, each compressor was reading the input data streamed via unix pipe ("|" in the command line). For decompression, each compressor was set up to stream decompressed data via pipe. This was done to better approximate a common pattern of using compressors in a practical data analysis scenario. In an actual sequence analysis workflow, often decompressed data is piped directly into a downstream analysis command. Also, when compressing the sequences, often the data is first pre-processed with another command, which then pipes processed sequences to a compressor.

Some compressors don't implement streaming mode, and only work with actual files. Since we have to benchmark all compressors on the same task, we added streaming mode to such compressors via wrapper scripts. For compression, a wrapper reads input data from "stdin" and writes it into a temporary file, then executes a compressor on that file, and finally deletes the file. For decompression the reverse process occurs: A wrapper executes decompressor which writes decompressed data into a temporary file, then reads this file and streams it to "stdout", before deleting the file.

The entire process is timed for the benchmark. Normally such wrapping has minimal impact on the overall compression/decompression speed, because we use fast SSD storage, and because actual compression and decompression takes comparatively much longer time than simply streaming the data to/from a file.

FASTA format compatibility

Many specialized compressors don't support the full-featured modern FASTA format, such as the one used in genome databases. Specifically, modern FASTA files often store masked sequence (use a mix of large and small letters), and include ambiguity codes. The degree of completeness of FASTA support varies wildly among compressors. At one end of the spectrum there are full featured compressors that support all FASTA format features. On another end, there are compressors that only work with a string of capital ACGT and nothing else, not even sequence names or newlines. Majority of sequence compressors are somewhere between these two extremes.

Essentially this means that each sequence compressor performs its own task, different from that of the others. If a compressor does not need to care about small vs capital letters, or about storing sequence names, it can possibly work faster. Thus comparing compressors each doing their own thing would not be fair, or very useful to the user. Since full-featured FASTA is in fact commonly used in today's databases, we decided to require complete lossless support of full-featured FASTA from all benchmarked compressors. In practice this means that we had to create a custom wrapper for each incomplete compressor, implementing the missing compatibility features.

A typical wrapper takes FASTA input transforms it into a format acceptable by the compressor being wrapped. For instance, if a compressor only expects capital sequence letters, then the positions of small and capital letters is extracted and saved in a separate file. The original file is converted to all uppercase, which is then fed to the compressor. The separate "mask" file (storing positions of lowercase letters) is compressed with a general purpose compressor. Entire set of files produces in such way counts for the compressed data size measured for this particular compressor and dataset, so that the overall compression strength is comparable with that achieved by other compressors (with or without their respective wrappers). Also the total time is measured, including all transformations and storing/compressing the additional files.

We developed several tools for quickly processing FASTA files to extract or add various channels of information for the purpose of wrapping incomplete compressors. We used C and optimized for speed, so that these steps have maximum speed and minimap impact on the overall compression. The wrapper scripts themselves are written in Perl. We used fast mode of zstd ("-1") to compress the additional files, chosen because of its high speed so that it has minimal impact on measuring the speed of the wrapped compressor.

As for compactness, the impact is minimal as well since the additional files are typically very small and compress well.

For all such wrapped compressors, we benchmarked not only the complete wrapped compressor, but also "wrapper-only" mode, in which only wrapper script is executed, but not the compressor itself. Such results are included in the benchmark under "wrap-NAME" names. This means that it's possible to compare the speed of entire wrapped compressor with "wrapper-only" run, for each dataset. This allows to see how much time is used by the wrapper, and therefore how much impact the wrapper makes on the overall results.

Some of the features implemented via wrappers:

- Supporting RNA input for DNA-only compressors
- Supporting 'N' in DNA/RNA sequences
- Supporting IUPAC's ambiguous nucleotide codes
- Saving and restoring line lengths
- Saving and restoring sequence names
- Saving and restoring sequence mask (upper/lower case)
- Supporting FASTA-formatted input
- Supporting input with more than 1 sequence

FASTQ compressors

Several FASTQ compressors are included in the benchmark. All of them are tested using wrappers which convert FASTA sequences into their respective accepted formats. Some need only adding artificial quality (constant "A" in most cases). Other expect only short reads or reads of identical lengths. These transformations are done in custom wrappers that we made for each FASTQ compressor. Since compression and decompression time recorded for benchmark is the total time of all steps, including wrapper processing, it means that in many cases the wrapped tool may work faster when used directly on FASTQ data. Also many FASTQ compressors are designed under additional assumptions typical for FASTQ data, for example that all reads are sampled from an underlying genome with substantial coverage (which allows meaningful assembly). These assumptions often don't hold on our FASTA-based benchmark datasets. Therefore all results of FASTQ compressors shown in our benchmark should not be taken as indicative of the actual performance of those compressors on FASTQ data that they were designed for.

Benchmark script availability

The benchmark script is available at "Benchmark-script/benchmark.pl" in Supplementary Code. All wrappers are available at "Website/tools/wrappers" in Supplementary Code. Additional tools used by the wrappers are available at "Website/tools/seq-tools-perl" and "Website/tools/seq-tools-c" in Supplementary Code. Compression and decompression commands are listed in files "Benchmark-script/compressors-*.txt" and "Benchmark-script/decompressors.txt" in Supplementary code. All these tools, wrappers and commands are also available at the SCB database website (http://kirr.dyndns.org/sequence-compression-benchmark/). The scripts are provided for reference only.

Website

Benchmark data is merged using script "Benchmark-script/2-collect-results.pl", available in Supplementary Code. The resulting merged data is then uploaded to the website where it is shown using a server-side Perl script. The script is available at "Website/index.cgi" in Supplementary Code. These scripts are provided for reference only.

Update plan

We plan to continue maintaining Sequence Compression Benchmark. This mainly involves benchmarking new or updated compressors, when such compressors become available. Since it's impractical to benchmark every existing compressor, we will continue to only benchmark compressors selected based on their performance, quality and usefulness for sequence compression.

Figure legends

Fig. 1. Comparison of 36 compressors on human genome. Best settings of each compressor are selected based on different aspects of performance: (A) compression ratio, (B) transfer + decompression speed, and (C) compression + transfer + decompression speed. Specialized sequence compressors are shown in orange color, and general-purpose compressors are shown in blue. The copy-compressor ("cat" command), shown in red color, is included as a control. The selected settings of each compressor are shown in their names, after hyphen. Multi-threaded compressors have "-1t" or "-4t" at the end of their names to indicate the number of threads used. Test data is the 3.31 GB reference human genome (accession number GCA_000001405.28).

Benchmark CPU: Intel Xeon E5-2643v3 (3.4 GHz). Link speed of 100 Mbit/s was used for estimating the transfer time.

Fig. 2. Comparison of 334 settings of 36 compressors on human genome. Each point represents a particular setting of some compressor. Panel A shows the relationship between compression ratio and decompression speed. Panel B shows the transfer + decompression speed plotted against compression + transfer + decompression speed. Test data is the 3.31 GB reference human genome (accession number GCA_000001405.28). Benchmark CPU: Intel Xeon E5-2643v3 (3.4 GHz). Link speed of 100 Mbit/s was used for estimating the transfer time.

Fig. 3. Comparison of compressor settings to gzip. Genome datasets were used as test data. Each point shows the performance of a compressor setting on specific genome test dataset. All values are shown relative to representative setting of gzip. Only performances that are at least half as good as gzip on both axes are shown. Panel A shows settings that performed best in Transfer+Decompression speed, B - settings that performed best in Compression+Transfer+Decompression speed. Link speed of 100 Mbit/s was used for estimating the transfer time.

Fig. 4. Compressor memory consumption. Strongest setting of each compressor is shown. On the X axis is the test data size. On the Y axis is the peak memory used by the compressor, for compression (A) and decompression (B).

Table 1. Compressor versions

A) Specialized sequence compressors

Compressor	Version
2bit	"faToTwoBit" and "twoBitToFa" binaries dated 2018-11-07
ac	AC 1.1, 2020-01-29
alapy	ALAPY 1.3.0, 2017-07-25
beetl	BEETL, commit 327cc65, 2019-11-14
blast	"convert2blastmask", "makeblastdb" and "blastdbcmd" binaries from BLAST 2.8.1+, 2018-11-26

dcom	DNA-COMPACT, latest public source 2013-08-29
dlim	DELIMINATE, version 1.3c, 2012
dnaX	dnaX 0.1.0, 2014-08-03
dsrc	DSRC 2.02, commit 5eda82c, 2015-06-04
fastqz	fastqz 1.5, commit 39b2bbc, 2012-03-15
fqs	FQSqueezer 0.1, commit 5741fc5, 2019-05-17
fqzcomp	fqzcomp 4.6, commit 96f2f61, 2019-12-02
geco	GeCo: v.2.1, 2016-12-24<br>GeCo2: v.1.1, 2019-02-02
gtz	GTX.Zip PROFESSIONAL-2.1.3-V-2020-03-18 07:11:20, binary
harc	HARC, commit cf35caf, 2019-10-04
jarvis	JARVIS v.1.1, commit d7daef5, 2019-04-30
kic	KIC binary, 0.2, 2015-11-25
leon	Leon, 1.0.0, 2016-02-27, Linux binary
lfastqc	LFastqC, commit 60e5fda, 2019-02-28, with fixes
lfqc	LFQC, commit 59f56e0, 2016-01-06
mfc	MFCompress,s1.01, 2013-09-03, 64-bit Linux binary
minicom	Minicom, commit 2360dd9, 2019-09-09
naf	NAF, 1.1.0, 2019-10-01
nuht	NUHT, commit 08a42a8, 2018-09-26, Linux binary
pfish	Pufferfish, v.1.0 alpha, 2012-04-11
quip	Quip, commit 9165bb5, 1.1.8-8-g9165bb5, 2017-12-17
spring	SPRING, commit 6536b1b, 2019-11-28
uht	UHT, binary from 2016-12-27
xm	XM (eXpert-Model), 3.0, commit 9b9ea57, 2019-01-07

B) General-purpose compressors

Compressor	Version
bcm	1.30, 2018-01-21
brieflz	1.3.0, 2020-02-15
brotli	1.0.7, 2018-10-23
bsc	3.1.0, 2016-01-01
bzip2	1.0.6, 2010-09-06
cmix	17, 2019-03-24
gzip	1.6, 2013-06-09
lizard	1.0.0, 2019-03-08
lz4	1.9.1, 2019-04-24
lzop	1.04, 2017-08-10
lzturbo	1.2, 2014-08-11

nakamichi	2020-May-09
pbzip2	1.1.13, 2015-12-18
pigz	2.4, 2017-12-26
snzip	1.0.4, 2016-10-02
xz	5.2.2, 2015-09-29
zpaq	7.15, 2016-08-17
zpipe	2.01, 2010-12-23
zstd	1.4.5, 2020-05-22

Table 2. Test datasets

A) Genome sequence datasets

**Category**	**Organism**	**Accession**	**Size**
Virus	*Gordonia phage GAL1* [60]	GCF_001884535.1	50.7 kB
Bacteria	*WS1 bacterium JGI 0000059-K21* [59]	GCA_000398605.1	522 kB
Protist	*Astrammina rara* [59]	GCA_000211355.2	1.71 MB
Fungus	*Nosema ceranae* [59]	GCA_000988165.1	5.81 MB
Protist	*Cryptosporidium parvum Iowa II* [59]	GCA_000165345.1	9.22 MB
Protist	*Spironucleus salmonicida* [59]	GCA_000497125.1	13.1 MB
Protist	*Tieghemostelium lacteum* [59]	GCA_001606155.1	23.7 MB
Fungus	*Fusarium graminearum PH-1* [60]	GCF_000240135.3	36.9 MB
Protist	*Salpingoeca rosetta* [59]	GCA_000188695.1	56.2 MB
Algae	*Chondrus crispus* [59]	GCA_000350225.2	106 MB
Algae	*Kappaphycus alvarezii* [59]	GCA_002205965.2	341 MB
Animal	*Strongylocentrotus purpuratus* [60]	GCF_000002235.4	1.01 GB
Plant	*Picea abies* [59]	GCA_900067695.1	13.4 GB

B) Other DNA datasets

**Dataset**	**Number of sequences**	**Size**	**Source**	**Date**
Mitochondrion [60]	9,402	245 MB	RefSeq FTP:<br>ftp://ftp.ncbi.nlm.nih.gov/refseq/release/<br>mitochondrion/mitochondrion.1.1.genomic.fna.gz<br>ftp://ftp.ncbi.nlm.nih.gov/refseq/release/<br>mitochondrion/mitochondrion.2.1.genomic.fna.gz	2019-03-15
NCBI Virus Complete Nucleotide Human [61]	36,745	482 MB	NCBI Virus:<br>https://www.ncbi.nlm.nih.gov/labs/virus/vssi/	2020-05-11
Influenza [62]	700,001	1.22 GB	Influenza Virus Database:<br>ftp://ftp.ncbi.nih.gov/genomes/INFLUENZA/<br>influenza.fna.gz	2019-04-27
Helicobacter [59]	108,292	2.76 GB	NCBI Assembly:<br>https://www.ncbi.nlm.nih.gov/assembly	2019-04-24

C) RNA datasets

Dataset	Number of sequences	Size	Source	Date
SILVA 132 LSURef [63]	198,843	610 MB	Silva database: https://ftp.arb-silva.de/release_132/Exports/ SILVA_132_LSURef_tax_silva.fasta.gz	2017-12-11
SILVA 132 SSURef Nr99 [63]	695,171	1.11 GB	Silva database: https://ftp.arb-silva.de/release_132/Exports/ SILVA_132_SSURef_Nr99_tax_silva.fasta.gz	2017-12-11
SILVA 132 SSURef [63]	2,090,668	3.28 GB	Silva database: https://ftp.arb-silva.de/release_132/Exports/ SILVA_132_SSURef_tax_silva.fasta.gz	2017-12-11

D) Multiple DNA sequence alignments

Dataset	Number of sequences	Size	Source	Date
UCSC hg38 7way knownCanonical-exonNuc [64]	1,470,154	340 MB	UCSC: https://hgdownload.soe.ucsc.edu/ goldenPath/hg38/multiz7way/alignments/ knownCanonical.exonNuc.fa.gz	2014-06-06
UCSC hg38 20way knownCanonical-exonNuc [64]	4,211,940	969 MB	UCSC: https://hgdownload.soe.ucsc.edu/ goldenPath/hg38/multiz20way/alignments/ knownCanonical.exonNuc.fa.gz	2015-06-30

E) Protein datasets

Dataset	Number of sequences	Size	Source	Date
PDB [65]	109,914	67.6 MB	PDB database FTP: ftp://ftp.ncbi.nih.gov/blast/db/FASTA/pdbaa.gz	2019-04-09
Homo sapiens GRCh38 [66]	105,961	73.2 MB	NCBI FTP: ftp://ftp.ensembl.org/ pub/release-96/fasta/homo_sapiens/pep/ Homo_sapiens.GRCh38.pep.all.fa.gz	2019-03-12
NCBI Virus RefSeq Protein [61]	373,332	122 MB	NCBI Virus: https://www.ncbi.nlm.nih.gov/labs/virus/vssi/	2020-05-10
UniProtKB Reviewed (Swiss-Prot) [67]	560,118	277 MB	UniProt FTP: ftp://ftp.uniprot.org/ pub/databases/uniprot/current_release/ knowledgebase/complete/uniprot_sprot.fasta.gz	2019-04-02

Manuscript with marked changes
Click here to access/download;Manuscript;Text-marked-changes.pdf

Sequence Compression Benchmark (SCB) database — a comprehensive evaluation of reference-free compressors for FASTA-formatted sequences

Kirill Kryukov*, Mahoko Takahashi Ueda, So Nakagawa, Tadashi Imanishi

Department of Molecular Life Science, Tokai University School of Medicine,

Isehara, Kanagawa 259-1193, Japan.

*Correspondence: kkryukov@gmail.com

Abstract

Background. Nearly all molecular sequence databases currently use gzip for data compression. Ongoing rapid accumulation of stored data calls for more efficient compression tool. Although numerous compressors exist, both specialized and general-purpose, choosing one of them was difficult because no comprehensive analysis of their comparative advantages for sequence compression was available.

Findings. We systematically benchmarked 430 settings of 48 compressors (including 29 specialized sequence compressors and 19 general-purpose compressors) on representative FASTA-formatted datasets of DNA, RNA and protein sequences. Each compressor was evaluated on 17 performance measures, including compression strength, as well as time and memory required for compression and decompression. We used 27 test datasets including individual genomes of various sizes, DNA and RNA datasets, and standard protein datasets. We summarized the results as the Sequence Compression Benchmark database (SCB database, http://kirr.dyndns.org/sequence-compression-benchmark/) that allows building custom visualizations for selected subsets of benchmark results.

Conclusion. We found that modern compressors offer large improvement in compactness and speed compared to gzip. Our benchmark allows comparing compressors and their settings using a variety of performance measures, offering the opportunity to select the optimal compressor based on the data type and usage scenario specific to particular application.

Keywords: compression; benchmark; DNA; RNA; protein; genome; sequence; database.

Background

Molecular sequence databases store and distribute DNA, RNA and protein sequences as compressed FASTA-formatted files. Biological sequence compression was first proposed in 1986 [1] and the first practical compressor was made in 1993 [2]. A lively field emerged that produced a stream of methods, algorithms, and software tools for sequence compression [3,4]. However, despite this activity, currently nearly all databases universally depend on gzip for compressing FASTA-formatted sequence data. This incredible longevity of the 27-year-old compressor probably owes to multiple factors, including conservatism of database operators, wide availability of gzip, and its generally acceptable performance. Through all these years the amount of stored sequence data kept growing steadily [5], increasing the load on database operators, users, storage systems and network infrastructure. However, someone thinking to replace gzip invariably faces the questions: which of the numerous available compressors to choose? And will the resulting gains be even worth the trouble of switching?

Previous attempts at answering these questions are limited by testing too few compressors and by using restricted test data [6-11]. In addition, all of these studies provide results in form of tables, with no graphical outputs, which makes the interpretation difficult. Existing benchmarks with useful visualization such as Squash [12], are limited to general-purpose compressors.

The variety of available specialized and general-purpose compressors is overwhelming. At the same time the field was lacking a thorough investigation of comparative merits of these compressors for sequence data. Therefore we set out to benchmark all available and practically useful compressors on a variety of relevant sequence data. Specifically, we focused on the common task of compressing DNA, RNA and protein sequences, stored in FASTA format, without using reference sequence. The benchmark results were shown in the Sequence Compression Benchmark database (SCB database, http://kirr.dyndns.org/sequence-compression-benchmark/).

Scope, compressors and test data

We considered the common scenario of archiving, transferring and working with large datasets of biological sequences. In this study we did not investigate compression of raw sequencing data in FASTQ

format, which was previously thorougly reviewed in [11]. Instead we focused on typical FASTA-formatted datasets, which includes individual genomes and single gene sets. Consequently we also did not consider referential compression, but only reference-free compression, which is typically used for such data. We evaluated standalone compression tools (rather than libraries), working under Linux OS on a modern workstation PC. In this study we only consider lossless compression.

We tested all DNA sequence compressors that are available and functional in 2020: dnaX [13], XM [14], DELIMINATE [15], Pufferfish [16], DNA-COMPACT [17], MFCompress [18], UHT [19], GeCo [20], GeCo2 [21], JARVIS [22], NAF [23], and NUHT [24]. We also included the relatively compact among homology search database formats: BLAST [25] and 2bit - a database format of BLAT [26].

Since compressors designed for FASTQ data can be trivially adopted for FASTA-formatted inputs, we also included a comprehensive array of compressors designed primarily or specifically for FASTQ data: BEETL [27], Quip [28], fastqz [10], fqzcomp [10], DSRC 2 [29], Leon [30], LFQC [31], KIC [32], ALAPY [33], GTX.Zip [34], HARC [35], LFastqC [36], SPRING [37], Minicom [38], and FQSqueezer [39]. We also included AC - a compressor designed exclusively for protein sequences [40]. We also tested a comprehensive array of general purpose compressors: bcm [41], brieflz[42], brotli [43], bsc [44], bzip2 [45], cmix [46], gzip [47], lizard [48], lz4 [49], lzop [50], lzturbo [51], nakamichi [52], pbzip2 [53], pigz [54], snzip [55], xz [56], zpaq [57], zpipe [57] and zstd [58]. See Table 1 for the list of compressors we used.

For the test data, we selected a variety of commonly used sequence datasets in FASTA format: (1) Individual genomes of various sizes, as examples of non-repetitive data [59,60]; (2) DNA and RNA datasets, such as collections of mitochondrial genomes, influenza virus sequences [60,61,62,59], 16S rRNA gene sequences [63], and genomic multiple DNA sequence alignments [64]; (3) Standard protein datasets [65,66,61,67]. Individual genomes are less repetitive, while other datasets are more repetitive. In total we used 27 test datasets. See Table 2 for the list of test data. All test data is available at the GigaDB repository.

Benchmark

We benchmarked each compressor on every test dataset, except in cases of incompatibility (e.g., DNA compressors cannot compress protein data) or excessive time requirement (some compressors are so slow that they would take weeks on larger datasets). For compressors with adjustable compression level, we

tested the relevant range of levels. We tested both 1 and 4-thread variants of compressors that support multi-threading. In total, we used 430 settings of 48 compressors. We also included the non-compressing "cat" command as control. For compressors using wrappers, we also benchmarked the wrappers.

Currently many sequence analysis tools support gzip-compressed files as input. Switching to another compressor may require either adding support of new format to those tools, or passing the data in uncompressed form. The latter solution can be achieved with the help of Unix pipes, if both the compressor and the analysis tool support streaming mode. Therefore, we benchmarked all compressors in streaming mode (streaming uncompressed data in both compression and decompression).

For each combination of compressor setting and test dataset we recorded compressed size, compression time, decompression time, peak compression memory and peak decompression memory. The details of the method and raw benchmark data are available in the Methods section and Supplementary Data, respectively. We share benchmark results and scripts at the SCM database website: http://kirr.dyndns.org/sequence-compression-benchmark/.

The choice of measure for evaluating compressor performance depends on prospective application. For long-term data storage, compactness may be the single most important criterion. For public sequence database, the key measure is how long time it takes from initiating the download of compressed files until accessing the decompressed data. This time consists of transfer time plus decompression time (TD-Time). Corresponding transfer-decompression speed (TD-Speed) is computed as Original Size / TD-Time. In this use case compression time is relatively unimportant, since compression happens only once, while transfer and decompression times affect every user of the database. For one-time data transfer, all three steps of compression, transfer and decompression are timed (CTD-Time), and used for computing the resulting overall speed (CTD-Speed).

A total of 17 measures, including the above-mentioned ones, are available in our results data (See Methods for the list of measures). Any of these measures can be used for selecting the best setting of each compressor and for sorting the list of compressors. These measures can be then shown in a table and visualized in column charts and scatterplots. This allows tailoring the output to answer specific questions, such as what compressor is better at compressing particular kind of data, or which setting of each compressor

performs best at particular task. The link speed that is used for estimating transfer times is configurable. The default speed of 100 Mbit/sec corresponds to the common speed of a fixed broadband internet connection.

Fig.1 compares the performance of best settings of 36 compressors on human genome. It shows that specialized sequence compressors achieve excellent compression ratio on this genome. However, when total TD-Speed or CTD-Speed is considered (measures that are important in practical applications), most sequence compressors fall behind the general-purpose ones. The best compressors for this dataset in terms of compression ratio, TD-Speed and CTD-Speed are "fastqz-slow", "naf-22" and "naf-1", respectively (numbers in each compressor name indicate compression level and other settings). Interestingly, the non-compressing "cat" command used as control, while naturally showing at the last place on compression ratio (Fig.1A), is not the slowest in terms of TD-Speed and CTD-Speed (Figs.1B and 1C, respectively). In case of CTD-Speed, for example, it means that some compressors are so slow that their compression + transfer + decompression time turns out to be longer than time required for transferring raw uncompressed data (using particular link speed, in this case 100 Mbit/sec).

Fig.2 compares all compressor settings on the same data (human genome). Fig.2A shows that the strongest compressors often provide very low decompression speed (shown using logarithmic scale due to the enormous range of values), which means that quick data transfer (resulting from strong compression) offered by those compressors is offset by significant waiting time required for decompressing the data. Fig.2B shows TD-Speed plotted against CTD-Speed. Similar figures can be constructed for other data and performance measures on the SCB database website.

Visualizing results from multiple test datasets simultaneously is possible, with or without aggregation of data. With aggregation, the numbers will be summed or averaged, and a single measurement will be shown for each setting of each compressor. Without aggregation, the results of each compressor setting will be shown separately on each dataset. Since the resulting number of data points can be huge, in such case it is useful to request only best settings of each compressor to be shown. The criteria for choosing the best setting is selectable among the 17 measurements. In case of a column chart, any of the 17 measures can be used for ordering the shown compressors, independently of the setting used for selecting best version, and independently for the measure actually shown in the chart.

One useful capability of the SCB database is showing measurements relative to specified compressor (and setting). This allows selecting a reference compressor and comparing the other compressors to this reference. For example, we can compare compressors to gzip as shown on Fig.3. In this example, we compare only best settings of each compressor, selected using specific measures (transfer+decompression speed and compression+transfer+decompression speed on Figs.3A and 3B, respectively). We also used fixed scale to show only range above 0.5 on both axes, which means that only performances that are at least half as good as gzip on both axes as shown. In this example, we can see that some compressors improve compactness and some improve speed compared to gzip, but few compressors improve both at the same time, such as lizard, naf, pigz, pbzip, and zstd.

It is important to be aware of the memory requirements when choosing a compressor (Fig.4). In these charts we plotted data size on the X axis, and disabled aggregation. This allows seeing how much memory a particular compressor used on each test dataset. As this example shows memory requirement reaches saturation point for most compressors. On the other hand, some compressors have unbounded growth of consumed memory, which makes then unusable for large data. Interestingly, gzip apparently has the smallest memory footprint, which may be one of the reasons for its popularity. Most compressors can function on a typical desktop hardware, but some require larger memory, which is important to consider when choosing a compressor that will be run by the consumers of distributed data.

A wide variety of charts can be produced on the benchmark website by selecting specific combinations of test data, compressors, and performance measures. At any point the currently visualized data can be obtained in textual form using Table output option. Also, all charts can be downloaded in SVG format.

Conclusions

Our benchmark reveals complex relationship between compressors and between their settings, based on various measures. We found that continued use of gzip is usually far from an optimal choice. Transitioning from gzip to a better compressor brings significant gains for genome and protein data, and is especially beneficial with repetitive DNA/RNA datasets. The optimal choice of compressor depends on many factors, including properties of the data to be compressed (such as sequence type, data size, and

amount of redundancy), relative importance of compression strength, compression speed and decompression speed for particular use scenario, as well as amount of memory available on data machines used for compression and decompression. Our benchmark allows comparing compressors on individual performance metrics, as well as on their combinations.

The Sequence Compression Benchmark (SCB) database will help in navigating the complex landscape of data compression. With dozens of compressors available, making an informed choice is not an easy task and requires careful analysis of the project requirements, data type and compressor capabilities. Our benchmark is the first resource providing a detailed practical evaluation of various compressors on a wide range of molecular sequence datasets. Using the SCB database, users can analyze compressor performances on variety of metrics, and construct custom reports for answering project-specific questions.

In contrast to previous studies that showed their results in static tables, our project is dynamic in two important senses: (1) the result tables and charts can be dynamically constructed for a custom selection of test data, compressors, and measured performance numbers, and (2) our study is not a one-off benchmark, but marks the start of a project where we will continue to add compressors and test data.

Making an informed choice of compressor with the help of our benchmark will lead to increased compactness of sequence databases, with shorter time required for downloading and decompressing. This will reduce the load on network and storage infrastructure, and increase speed and efficiency in biological and medical research.

Declarations

Availability of data and material

All benchmark data is available at the online SCB database:
http://kirr.dyndns.org/sequence-compression-benchmark/

Competing interests

The authors declare no competing interests.

Funding

This work was supported by the 2019 Tokai University School of Medicine Research Aid (to KK), JSPS KAKENHI Grants-in-Aid for Scientific Research (C) (20K06612 to KK) and Scientific Research on Innovative Areas (16H06429, 16K21723, 19H04843 to SN), and Takeda Science Foundation (to TI).

Authors' contributions

KK conceived the study idea and implemented the benchmark. SN provided benchmark hardware. KK, MTU, SN and TI interpreted the data and wrote the manuscript. KK and MTU prepared figures and tables. All authors read and approved the final manuscript.

Acknowledgements

Not applicable.

References

1. Walker JR, Willett P. Compression of nucleic acid and protein sequence data. *Comput. Appl. Biosci.* 1986;2(2):89-93.
2. Grumbach S, Tahi F. Compression of DNA sequences. *Data Compression Conference*, Snowbird, Utah, IEEE Computer Society. 1993. p. 340-50. doi:10.1109/DCC.1993.253115.
3. Deorowicz S, Grabowski S. Data compression for sequencing data. *Algorithms for Molecular Biology*. 2013;8:25. doi:10.1186/1748-7188-8-25.
4. Hernaez M, Pavlichin D, Weissman T, Ochoa I. Genomic Data Compression. *Annual Review of Biomedical Data Science*. 2019;2:19-37. doi:10.1146/annurev-biodatasci-072018-021229.
5. Karsch-Mizrachi I, Takagi T, Cochrane G. The international nucleotide sequence database collaboration. *Nucleic Acids Res.* 2018;46(Database issue):D48–D51. doi:10.1093/nar/gkx1097.
6. Zhu Z, Zhang Y, Ji Z, He S, Yang X. High-throughput DNA sequence data compression. *Brief. Bioinform.* 2013; 16(1):1-15. doi:10.1093/bib/bbt087.
7. Hosseini M, Pratas D, Pinho AJ. A Survey on Data Compression Methods for Biological Sequences. *Information*. 2016;7(4):56. doi:10.3390/info7040056.
8. Sardaraz M, Tahir M. Advances in high throughput DNA sequence data compression. *J. Bioinform. Comput. Biol*. 2016;14(3):1630002. doi:10.1142/S0219720016300021.

9. Biji CL, Achuthsankar SN. Benchmark Dataset for Whole Genome Sequence Compression. *IEEE/ACM Trans. Comput. Biol. Bioinform.* 2017;14(6):1228-36. doi:10.1109/TCBB.2016.2568186.
10. Bonfield JK, Mahoney MV. Compression of FASTQ and SAM Format Sequencing Data. *PLoS One*. 2013;8(3): e59190, doi:10.1371/journal.pone.0059190.
11. Numanagic I, Bonfield JK, Hach F, Voges J, Ostermann J, Alberti C, Mattavelli M. Comparison of high-throughput sequencing data compression tools. *Nature Methods*. 2016;13(12):1005-8, doi:10.1038/nmeth.4037.
12. Squash Compression Benchmark. 2015. https://quixdb.github.io/squash-benchmark/. Accessed July 15, 2019.
13. Manzini G, Rastero M. A simple and fast DNA compressor. *Software - Practice and Experience.* 2004;34:1397-411, doi:10.1002/spe.619.
14. Cao MD, Dix TI, Allison L. Mears C. A simple statistical algorithm for biological sequence compression. *Data Compression Conference*. DCC '07, Snowbird, UT, IEEE Computer Society. 2007. p. 43-52. doi:10.1109/DCC.2007.7.
15. Mohammed MH, Dutta A, Bose T, Chadaram S, Mande SS. DELIMINATE — a fast and efficient method for loss-less compression of genomic sequences. *Bioinformatics*. 2012;28:2527–29. doi:10.1093/bioinformatics/bts467.
16. Pufferfish. 2012. https://github.com/alexholehouse/pufferfish. Accessed May 23, 2019.
17. Li P, Wang S, Kim J, Xiong H, Ohno-Machado L, Jiang X. DNA-COMPACT: DNA COMpression Based on a Pattern-Aware Contextual Modeling Technique. *PLoS ONE.* 2013;8(11):e80377. doi:10.1371/journal.pone.0080377.
18. Pinho AJ, Pratas D. MFCompress: a compression tool for FASTA and multi-FASTA data. *Bioinformatics*. 2014;30:117-8. doi:10.1093/bioinformatics/btt594.
19. Al-Okaily A, Almarri B, Al Yami S, Huang CH. Toward a Better Compression for DNA Sequences Using Huffman Encoding. *J. Comp. Biol.* 2017;24(4):280–8. doi:10.1089/cmb.2016.0151.
20. Pratas D, Pinho AJ, Ferreira PJSG. Efficient compression of genomic sequences. *Data Compression Conference*, DCC-2016, Snowbird, Utah, IEEE Computer Society. 2016. p.231-240. doi: 10.1109/DCC.2016.60.

21. Pratas D, Hosseini M, Pinho AJ. GeCo2: An Optimized Tool for Lossless Compression and Analysis of DNA Sequences. *Practical Applications of Computational Biology and Bioinformatics, 13th International Conference*, PACBB 2019, Advances in Intelligent Systems and Computing, vol 1005, Springer, Cham, 2019a. p.137-145. doi: 10.1007/978-3-030-23873-5_17.
22. Pratas D, Hosseini M, Silva J, Pinho AJ. A Reference-Free Lossless Compression Algorithm for DNA Sequences Using a Competitive Prediction of Two Classes of Weighted Models. *Entropy*, 2019b;21:1074. doi:10.3390/e21111074.
23. Kryukov K, Ueda MT, Nakagawa S, Imanishi T. Nucleotide Archival Format (NAF) enables efficient lossless reference-free compression of DNA sequences. *Bioinformatics.* 2019;35(19):3826-28. doi:10.1093/bioinformatics/btz144.
24. Alyami S, Huang CH. Nongreedy Unbalanced Huffman Tree Compressor for Single and Multifasta Files. Journal of Computational Biology. 2019; 26(0):1-9. doi:10.1089/cmb.2019.0249.
25. Altschul SF, Gish W, Miller W, Myers EW, Lipman DJ. Basic local alignment search tool. *J. Mol. Biol.* 1990;215(3):403-10. doi:10.1016/S0022-2836(05)80360-2.
26. Kent WJ. BLAT - The BLAST-Like Alignment Tool. *Genome Research.* 2002;12(4):656-64. doi:10.1101/gr.229202.
27. Bauer MJ, Cox AJ, Rosone G. Lightweight BWT Construction for Very Large String Collections. *Combinatorial Pattern Matching 2011*, proceedings of the CPM 2011, 2011. p.219-231. doi:10.1007/978-3-642-21458-5_20.
28. Jones DC, Ruzzo WL, Peng X, Katze MG. Compression of next-generation sequencing reads aided by highly efficient de novo assembly. *Nucleic Acids Research.* 2012;40(22):e171. doi:10.1093/nar/gks754.
29. Roguski L, Deorowicz S. DSRC 2—Industry-oriented compression of FASTQ files. *Bioinformatics.* 2014; 30(15):2213-5. doi:10.1093/bioinformatics/btu208.
30. Benoit G, Lemaitre C, Lavenier D, Drezen E, Dayris T, Uricaru R, Rizk G. Reference-free compression of high throughput sequencing data with a probabilistic de Bruijn graph. *BMC Bioinformatics*. 2015;16:288. doi:10.1186/s12859-015-0709-7.
31. Nicolae M, Pathak S, Rajasekaran S. LFQC: a lossless compression algorithm for FASTQ files. *Bioinformatics.* 2015;31(20):3276-81. doi:10.1093/bioinformatics/btv384.

32. Zhang Y, Patel K, Endrawis T, Bowers A, Sun Y. A FASTQ compressor based on integer-mapped k-mer indexing for biologist. *Gene.* 2016;579(1):75-81. doi:10.1016/j.gene.2015.12.053.
33. ALAPY 2017. http://alapy.com/services/alapy-compressor/. Accessed December 2, 2019.
34. Xing Y, Li G, Wang Z, Feng B, Song Z, Wu C. GTZ: a fast compression and cloud transmission tool optimized for FASTQ files. *BMC Bioinformatics.* 2017;18(Suppl 16):549. doi:10.1186/s12859-017-1973-5.
35. Chandak S, Tatwawadi K, Weissman T. Compression of genomic sequencing reads via hash-based reordering: algorithm and analysis. *Bioinformatics.* 2018;34(4):558-67. doi:10.1093/bioinformatics/btx639.
36. Al Yami S, Huang CH. LFastqC: A lossless non-reference-based FASTQ compressor. PLoS One. 2019;14(11):e0224806, doi:10.1371/journal.pone.0224806.
37. Chandak S, Tatwawadi K, Ochoa I, Hernaez M, Weissman T. SPRING: a next-generation compressor for FASTQ data. Bioinformatics. 2019;35(15):2674-6. doi:10.1093/bioinformatics/bty1015.
38. Liu Y, Yu Z, Dinger ME, Li J. Index suffix-prefix overlaps by (w, k)-minimizer to generate long contigs for reads compression. Bioinformatics. 2019. 35(12):2066-2074, doi:10.1093/bioinformatics/bty936.
39. Deorowicz S. FQSqueezer: k-mer-based compression of sequencing data. Scientific Reports. 2020;10:578. doi:10.1038/s41598-020-57452-6.
40. Hosseini M, Pratas D, Pinho AJ. AC: A Compression Tool for Amino Acid Sequences. Interdisciplinary Sciences: Computational Life Sciences. 2019;11:68-76. doi:10.1007/s12539-019-00322-1.
41. BCM. https://github.com/encode84/bcm. Accessed June 6 2019.
42. BriefLZ - small fast Lempel-Ziv. https://github.com/jibsen/brieflz. Accessed May 12 2020.
43. Alakuijala J, Szabadka Z. Brotli Compressed Data Format. RFC 7932. 2016. Accessed April 14 2019.
44. libbsc. https://github.com/IlyaGrebnov/libbsc. Accessed June 22 2019.
45. bzip2. https://www.sourceware.org/bzip2/. Accessed January 20 2019.
46. cmix. https://github.com/byronknoll/cmix. Accessed April 25 2019.
47. GNU Gzip. https://www.gnu.org/software/gzip/. Accessed November 8 2019.
48. Lizard - efficient compression with very fast decompression. https://github.com/inikep/lizard. Accessed June 16 2019.

49. LZ4 - Extremely fast compression. https://github.com/lz4/lz4. Accessed April 25 2019.
50. Lzop. 2017. https://www.lzop.org/. Accessed December 6 2018.
51. LzTurbo - World's fastest compressor. https://sites.google.com/site/powturbo/. Accessed February 11 2019.
52. Nakamichi. http://www.sanmayce.com/Nakamichi/index.html. Accessed May 12 2020.
53. pbzip2. https://launchpad.net/pbzip2/. Accessed April 26 2019.
54. pigz. https://zlib.net/pigz/. Accessed April 26 2019.
55. Snzip, a compression/decompression tool based on snappy. https://github.com/kubo/snzip. Accessed November 11 2018.
56. XZ Utils. https://tukaani.org/xz/. Accessed December 17 2018.
57. ZPAQ Incremental Journaling Backup Utility and Archiver. http://www.mattmahoney.net/dc/zpaq.html. Accessed November 7 2018.
58. Zstandard - Fast real-time compression algorithm. https://github.com/facebook/zstd. Accessed May 22 2020.
59. Clark K, Karsch-Mizrachi I, Lipman DJ, Ostell J, Sayers EW. GenBank. *Nucleic Acids Res.* 2016;44(D1):D67–D72. doi:10.1093/nar/gkv1276.
60. O'Leary NA, Wright MW, Brister JR, Ciufo S, Haddad D, McVeigh R, et al. Reference sequence (RefSeq) database at NCBI: current status, taxonomic expansion, and functional annotation. *Nucleic Acids Res.* 2016;44(D1):D733-45. doi:10.1093/nar/gkv1189.
61. Brister JR, Ako-Adjei D, Bao Y, Blinkova O. NCBI viral genomes resource. *Nucleic Acids Res.* 2015;43(D1):D571-7. doi:10.1093/nar/gku1207.
62. Bao Y, Bolotov P, Dernovoy D, Kiryutin B, Zaslavsky L, Tatusova T, Ostell J, Lipman D. The Influenza Virus Resource at the National Center for Biotechnology Information. *J Virol.* 2008;82(2):596-601. doi:10.1128/JVI.02005-07.
63. Quast C, Pruesse E, Yilmaz P, Gerken J, Schweer T, Yarza P, Peplies J, Glöckner FO. The SILVA ribosomal RNA gene database project: improved data processing and web-based tools. *Nucl. Acids Res.* 2013;41(D1):D590-D596. doi:10.1093/nar/gks1219.

64. Kent WJ, Sugnet CW, Furey TS, Roskin KM, Pringle TH, Zahler AM, Haussler D. The human genome browser at UCSC. *Genome Res.* 2002;12(6):996-1006. doi:10.1101/gr.229102.

65. Berman HM, Westbrook J, Feng Z, Gilliland G, Bhat TN, Weissig H, Shindyalov IN, Bourne PE. The Protein Data Bank. *Nucleic Acids Res.* 2000;28:235-42. doi:10.1093/nar/28.1.235.

66. Yates AD, Achuthan P, Akanni W, Allen J, Allen J, Alvarez-Jarreta J, et al. Ensembl 2020. *Nucleic Acids Res.* 2020;48(D1):D682–8. doi:10.1093/nar/gkz966.

67. The UniProt Consortium. UniProt: a worldwide hub of protein knowledge. *Nucleic Acids Res.* 2019;47(D1):D506-15. doi:10.1093/nar/gky1049.

Methods

Benchmarked task

The task is to compress and decompress a FASTA-formatted file containing DNA, RNA or protein sequences. The process has to be lossless, i.e., decompressed data must be byte-to-byte identical to the original data. Compression and decompression are done without using any reference genome. Each compression and decompression task is executed via a Linux in a command line interface. Input data for compression and output data during decompression are streamed using Unix pipes.

Only well-formed FASTA files are used in the benchmark: They must contain no empty lines and all long sequence lines have to be wrapped at the same position. Both large and small (soft-masked) letters can be present, as well as common ambiguity codes. In multiple sequence alignments, additionally, dash ("-") is used for indicating gaps. Each test dataset is compressed separately from other datasets.

Compressor selection

We used all specialized sequence compressors that we could find and make to work for the above specified task. For general-purpose compressors we used only the major ones, in terms of performance, historical importance, or popularity. For each compressor with configurable compression level (or other parameters related to compression strength of speed), we used the relevant range of settings, including the default.

Benchmark machine

- CPU: dual Xeon E5-2643v3 (3.4 GHz, 6 cores), hyperthreading: off
- RAM: 128 GB DDR4-2133 ECC Registered
- Storage: 4 x 2 TB SSD, in RAID 0, XFS filesystem, block size: 4096 bytes (blockdev --getbsz)
- OS: Ubuntu 18.04.1 LTS, kernel: 4.15.0
- GCC: 7.4.0

Compressor/dataset combinations that were tested

Each setting of each compressor is tested on every test dataset, except when it's difficult or impossible due to compressor limitations:

- AC is a protein-specific compressor, and was tested only on protein datasets.
- Due to their extreme slowness, these compressors are not tested on any data larger than 10 MB: cmix, DNA-COMPACT, GeCo, JARVIS, Leon, and XM.
- UHT fails on the 245 MB dataset and on larger data.
- Nakamichi was only used on data smaller than 200 MB due to its slowness and memory requirements.
- Among sequence compressors, only DELIMINATE, MFCompress and NAF support multiple sequence alignments.
- Among sequence compressors, only AC, BLAST and NAF support protein sequences.
- Some settings of XM crash and/or produce wrong decompressed output on some data - such results are not included.
- NUHT's memory requirement makes it impossible to use on 13.4 GB *Picea abies* genome.
- LFastqC fails on 2.7 GB dataset and larger data.

Benchmark process

The entire benchmark is orchestrated by a perl script. This script loads the lists of compressor settings and test data, and proceeds to test each combination that still has its measurements missing in the output directory. For each such combination (of compressor setting and test dataset), the following steps are performed:

1. Compression is performed by piping the test data into the compressor. Compressed size and compression time is recorded. For compressed formats consisting of multiple files, sizes of all files are summed together.
2. If compression time did not exceed 10 seconds, 9 more compression runs are performed, recording compression times. Compressed data from previous run is deleted before each next compression run.
3. The next set of compression runs is performed to measure peak memory consumption. This set consists of the same number of runs as in steps 1-2 (either 1 or 10 runs). That is, for fast compressors and for small data the measurement is repeated 10 times.
4. Decompression test run is performed. In this run decompressed data is piped to the "md5sum -b -" command. The resulting md5 signature is compared with that of the original file. In case of any mismatch this combination of compressor setting and dataset is disqualified and its measurements are discarded.
5. Decompression time is measured. This time decompressed data is piped to /dev/null.
6. If decompression completed within 10 seconds, 9 more decompression runs are performed and timed.
7. Peak decompression memory is measured. The number of runs is same as in steps 5-6.
8. The measurements are stored to a file. All compressed and temporary files are removed.

Measurement methods

Measuring time: Wall clock time was measured using Perl's Time::HiRes module (gettimeofday and tv_interval subroutines). The resulting time was recorded with millisecond precision.

Measuring peak memory consumption: First, each compression command was stored in a temporary shell script file. Then it was executed via GNU Time, as /usr/bin/time -v cmd.sh >output.txt. "Maximum resident set size" value was extracted from the output. 1638 was then subtracted from this value and the result was stored as peak memory measurement. 1638 is the average "Maximum resident set size" measured by GNU Time in the same way for an empty shell script.

Memory consumption and time were measured separately because measuring memory makes the task slower, especially for very fast tasks.

Collected measurements

For each combination of compressor and dataset that was tested, the following measurements were collected:

- Compressed size (in bytes)
- Compression time (in milliseconds)
- Decompression time (in milliseconds)
- Peak compression memory (in GNU Time's "Kbytes")
- Peak decompression memory (in GNU Time's "Kbytes")

In cases where 10 values are collected, the average value is used by the benchmark web-site.

Computed values

The following values were calculated based on the measured values:

- Compressed size relative to original (%) = Compressed size / Uncompressed size * 100
- Compression ratio (times) = Uncompressed size / Compressed size
- Compression speed (MB/s) = Uncompressed size in MB / Compression time
- Decompression speed (MB/s) = Uncompressed size in MB / Decompression time
- Compression + decompression time (s) = Compression time + Decompression time
- Compression + decompression speed (MB/s) = Uncompressed size in MB / (Compression time + Decompression time)
- Transfer time (s) = Uncompressed size / Link speed in B/s
- Transfer speed (MB/s) = Uncompressed size in MB / Transfer time
- Transfer + decompression time (s) = Transfer time + Decompression time
- Transfer + decompression speed (MB/s) = Uncompressed size in MB / (Transfer time + Decompression time)
- Compression + transfer + decompression time (s) = Compression time + Transfer time + Decompression time
- Compression + transfer + decompression speed (MB/s) = Uncompressed size in MB / (Compression time + Transfer time + Decompression time)

Rationale for non-constant number of runs

Variable number of runs is the only way to have both accurate measurements and large test data (under the constraints of using one test machine, and running benchmark within reasonable time).

On one hand, benchmark takes lot of time. So much that some compressors can't be even tested at all on dataset larger than 10 MB in reasonable time. Therefore repeating every measurement 10 times is impractical. Or, it would imply restricting the test data to only small datasets.

On the other hand, measurements are slightly noisy. The shorter measured time, the more noisy its measurement. Thus for very quick runs, multiple runs allow for substantial noise suppression. For longer runs it does not make much difference, because the relative error is already small with longer times.

Using a threshold of 10 seconds seems to be a reasonable compromise between suppressing noise and including larger test data (and slow compressors).

Streaming mode

For compression, each compressor was reading the input data streamed via unix pipe ("|" in the command line). For decompression, each compressor was set up to stream decompressed data via pipe. This was done to better approximate a common pattern of using compressors in a practical data analysis scenario. In an actual sequence analysis workflow, often decompressed data is piped directly into a downstream analysis command. Also, when compressing the sequences, often the data is first pre-processed with another command, which then pipes processed sequences to a compressor.

Some compressors don't implement streaming mode, and only work with actual files. Since we have to benchmark all compressors on the same task, we added streaming mode to such compressors via wrapper scripts. For compression, a wrapper reads input data from "stdin" and writes it into a temporary file, then executes a compressor on that file, and finally deletes the file. For decompression the reverse process occurs: A wrapper executes decompressor which writes decompressed data into a temporary file, then reads this file and streams it to "stdout", before deleting the file.

The entire process is timed for the benchmark. Normally such wrapping has minimal impact on the overall compression/decompression speed, because we use fast SSD storage, and because actual compression and decompression takes comparatively much longer time than simply streaming the data to/from a file.

FASTA format compatibility

Many specialized compressors don't support the full-featured modern FASTA format, such as the one used in genome databases. Specifically, modern FASTA files often store masked sequence (use a mix of large and small letters), and include ambiguity codes. The degree of completeness of FASTA support varies wildly among compressors. At one end of the spectrum there are full featured compressors that support all FASTA format features. On another end, there are compressors that only work with a string of capital ACGT and nothing else, not even sequence names or newlines. Majority of sequence compressors are somewhere between these two extremes.

Essentially this means that each sequence compressor performs its own task, different from that of the others. If a compressor does not need to care about small vs capital letters, or about storing sequence names, it can possibly work faster. Thus comparing compressors each doing their own thing would not be fair, or very useful to the user. Since full-featured FASTA is in fact commonly used in today's databases, we decided to require complete lossless support of full-featured FASTA from all benchmarked compressors. In practice this means that we had to create a custom wrapper for each incomplete compressor, implementing the missing compatibility features.

A typical wrapper takes FASTA input transforms it into a format acceptable by the compressor being wrapped. For instance, if a compressor only expects capital sequence letters, then the positions of small and capital letters is extracted and saved in a separate file. The original file is converted to all uppercase, which is then fed to the compressor. The separate "mask" file (storing positions of lowercase letters) is compressed with a general purpose compressor. Entire set of files produces in such way counts for the compressed data size measured for this particular compressor and dataset, so that the overall compression strength is comparable with that achieved by other compressors (with or without their respective wrappers). Also the total time is measured, including all transformations and storing/compressing the additional files.

We developed several tools for quickly processing FASTA files to extract or add various channels of information for the purpose of wrapping incomplete compressors. We used C and optimized for speed, so that these steps have maximum speed and minimap impact on the overall compression. The wrapper scripts themselves are written in Perl. We used fast mode of zstd ("-1") to compress the additional files, chosen because of its high speed so that it has minimal impact on measuring the speed of the wrapped compressor.

As for compactness, the impact is minimal as well since the additional files are typically very small and compress well.

For all such wrapped compressors, we benchmarked not only the complete wrapped compressor, but also "wrapper-only" mode, in which only wrapper script is executed, but not the compressor itself. Such results are included in the benchmark under "wrap-NAME" names. This means that it's possible to compare the speed of entire wrapped compressor with "wrapper-only" run, for each dataset. This allows to see how much time is used by the wrapper, and therefore how much impact the wrapper makes on the overall results.

Some of the features implemented via wrappers:

- Supporting RNA input for DNA-only compressors
- Supporting 'N' in DNA/RNA sequences
- Supporting IUPAC's ambiguous nucleotide codes
- Saving and restoring line lengths
- Saving and restoring sequence names
- Saving and restoring sequence mask (upper/lower case)
- Supporting FASTA-formatted input
- Supporting input with more than 1 sequence

FASTQ compressors

Several FASTQ compressors are included in the benchmark. All of them are tested using wrappers which convert FASTA sequences into their respective accepted formats. Some need only adding artificial quality (constant "A" in most cases). Other expect only short reads or reads of identical lengths. These transformations are done in custom wrappers that we made for each FASTQ compressor. Since compression and decompression time recorded for benchmark is the total time of all steps, including wrapper processing, it means that in many cases the wrapped tool may work faster when used directly on FASTQ data. Also many FASTQ compressors are designed under additional assumptions typical for FASTQ data, for example that all reads are sampled from an underlying genome with substantial coverage (which allows meaningful assembly). These assumptions often don't hold on our FASTA-based benchmark datasets. Therefore all results of FASTQ compressors shown in our benchmark should not be taken as indicative of the actual performance of those compressors on FASTQ data that they were designed for.

Benchmark script availability

The benchmark script is available at "Benchmark-script/benchmark.pl" in Supplementary Code. All wrappers are available at "Website/tools/wrappers" in Supplementary Code. Additional tools used by the wrappers are available at "Website/tools/seq-tools-perl" and "Website/tools/seq-tools-c" in Supplementary Code. Compression and decompression commands are listed in files "Benchmark-script/compressors-*.txt" and "Benchmark-script/decompressors.txt" in Supplementary code. All these tools, wrappers and commands are also available at the SCB database website (http://kirr.dyndns.org/sequence-compression-benchmark/). The scripts are provided for reference only.

Website

Benchmark data is merged using script "Benchmark-script/2-collect-results.pl", available in Supplementary Code. The resulting merged data is then uploaded to the website where it is shown using a server-side Perl script. The script is available at "Website/index.cgi" in Supplementary Code. These scripts are provided for reference only.

Update plan

We plan to continue maintaining Sequence Compression Benchmark. This mainly involves benchmarking new or updated compressors, when such compressors become available. Since it's impractical to benchmark every existing compressor, we will continue to only benchmark compressors selected based on their performance, quality and usefulness for sequence compression.

Figure legends

Fig. 1. Comparison of 36 compressors on human genome. Best settings of each compressor are selected based on different aspects of performance: (A) compression ratio, (B) transfer + decompression speed, and (C) compression + transfer + decompression speed. Specialized sequence compressors are shown in orange color, and general-purpose compressors are shown in blue. The copy-compressor ("cat" command), shown in red color, is included as a control. The selected settings of each compressor are shown in their names, after hyphen. Multi-threaded compressors have "-1t" or "-4t" at the end of their names to indicate the number of threads used. Test data is the 3.31 GB reference human genome (accession number GCA_000001405.28).

Benchmark CPU: Intel Xeon E5-2643v3 (3.4 GHz). Link speed of 100 Mbit/s was used for estimating the transfer time.

Fig. 2. Comparison of 334 settings of 36 compressors on human genome. Each point represents a particular setting of some compressor. Panel A shows the relationship between compression ratio and decompression speed. Panel B shows the transfer + decompression speed plotted against compression + transfer + decompression speed. Test data is the 3.31 GB reference human genome (accession number GCA_000001405.28). Benchmark CPU: Intel Xeon E5-2643v3 (3.4 GHz). Link speed of 100 Mbit/s was used for estimating the transfer time.

Fig. 3. Comparison of compressor settings to gzip. Genome datasets were used as test data. Each point shows the performance of a compressor setting on specific genome test dataset. All values are shown relative to representative setting of gzip. Only performances that are at least half as good as gzip on both axes are shown. Panel A shows settings that performed best in Transfer+Decompression speed, B - settings that performed best in Compression+Transfer+Decompression speed. Link speed of 100 Mbit/s was used for estimating the transfer time.

Fig. 4. Compressor memory consumption. Strongest setting of each compressor is shown. On the X axis is the test data size. On the Y axis is the peak memory used by the compressor, for compression (A) and decompression (B).

Table 1. Compressor versions

A) Specialized sequence compressors

Compressor	Version
2bit	"faToTwoBit" and "twoBitToFa" binaries dated 2018-11-07
ac	AC 1.1, 2020-01-29
alapy	ALAPY 1.3.0, 2017-07-25
beetl	BEETL, commit 327cc65, 2019-11-14
blast	"convert2blastmask", "makeblastdb" and "blastdbcmd" binaries from BLAST 2.8.1+, 2018-11-26

dcom	DNA-COMPACT, latest public source 2013-08-29
dlim	DELIMINATE, version 1.3c, 2012
dnaX	dnaX 0.1.0, 2014-08-03
dsrc	DSRC 2.02, commit 5eda82c, 2015-06-04
fastqz	fastqz 1.5, commit 39b2bbc, 2012-03-15
fqs	FQSqueezer 0.1, commit 5741fc5, 2019-05-17
fqzcomp	fqzcomp 4.6, commit 96f2f61, 2019-12-02
geco	GeCo: v.2.1, 2016-12-24<br>GeCo2: v.1.1, 2019-02-02
gtz	GTX.Zip PROFESSIONAL-2.1.3-V-2020-03-18 07:11:20, binary
harc	HARC, commit cf35caf, 2019-10-04
jarvis	JARVIS v.1.1, commit d7daef5, 2019-04-30
kic	KIC binary, 0.2, 2015-11-25
leon	Leon, 1.0.0, 2016-02-27, Linux binary
lfastqc	LFastqC, commit 60e5fda, 2019-02-28, with fixes
lfqc	LFQC, commit 59f56e0, 2016-01-06
mfc	MFCompress,s1.01, 2013-09-03, 64-bit Linux binary
minicom	Minicom, commit 2360dd9, 2019-09-09
naf	NAF, 1.1.0, 2019-10-01
nuht	NUHT, commit 08a42a8, 2018-09-26, Linux binary
pfish	Pufferfish, v.1.0 alpha, 2012-04-11
quip	Quip, commit 9165bb5, 1.1.8-8-g9165bb5, 2017-12-17
spring	SPRING, commit 6536b1b, 2019-11-28
uht	UHT, binary from 2016-12-27
xm	XM (eXpert-Model), 3.0, commit 9b9ea57, 2019-01-07

B) General-purpose compressors

Compressor	Version
bcm	1.30, 2018-01-21
brieflz	1.3.0, 2020-02-15
brotli	1.0.7, 2018-10-23
bsc	3.1.0, 2016-01-01
bzip2	1.0.6, 2010-09-06
cmix	17, 2019-03-24
gzip	1.6, 2013-06-09
lizard	1.0.0, 2019-03-08
lz4	1.9.1, 2019-04-24
lzop	1.04, 2017-08-10
lzturbo	1.2, 2014-08-11

nakamichi	2020-May-09
pbzip2	1.1.13, 2015-12-18
pigz	2.4, 2017-12-26
snzip	1.0.4, 2016-10-02
xz	5.2.2, 2015-09-29
zpaq	7.15, 2016-08-17
zpipe	2.01, 2010-12-23
zstd	1.4.5, 2020-05-22

Table 2. Test datasets

A) Genome sequence datasets

Category	Organism	Accession	Size
Virus	*Gordonia phage GAL1* [60]	GCF_001884535.1	50.7 kB
Bacteria	*WS1 bacterium JGI 0000059-K21* [59]	GCA_000398605.1	522 kB
Protist	*Astrammina rara* [59]	GCA_000211355.2	1.71 MB
Fungus	*Nosema ceranae* [59]	GCA_000988165.1	5.81 MB
Protist	*Cryptosporidium parvum Iowa II* [59]	GCA_000165345.1	9.22 MB
Protist	*Spironucleus salmonicida* [59]	GCA_000497125.1	13.1 MB
Protist	*Tieghemostelium lacteum* [59]	GCA_001606155.1	23.7 MB
Fungus	*Fusarium graminearum PH-1* [60]	GCF_000240135.3	36.9 MB
Protist	*Salpingoeca rosetta* [59]	GCA_000188695.1	56.2 MB
Algae	*Chondrus crispus* [59]	GCA_000350225.2	106 MB
Algae	*Kappaphycus alvarezii* [59]	GCA_002205965.2	341 MB
Animal	*Strongylocentrotus purpuratus* [60]	GCF_000002235.4	1.01 GB
Plant	*Picea abies* [59]	GCA_900067695.1	13.4 GB

B) Other DNA datasets

Dataset	Number of sequences	Size	Source	Date
Mitochondrion [60]	9,402	245 MB	RefSeq FTP:<br>ftp://ftp.ncbi.nlm.nih.gov/refseq/release/mitochondrion/mitochondrion.1.1.genomic.fna.gz<br>ftp://ftp.ncbi.nlm.nih.gov/refseq/release/mitochondrion/mitochondrion.2.1.genomic.fna.gz	2019-03-15
NCBI Virus Complete Nucleotide Human [61]	36,745	482 MB	NCBI Virus:<br>https://www.ncbi.nlm.nih.gov/labs/virus/vssi/	2020-05-11
Influenza [62]	700,001	1.22 GB	Influenza Virus Database:<br>ftp://ftp.ncbi.nih.gov/genomes/INFLUENZA/influenza.fna.gz	2019-04-27
Helicobacter [59]	108,292	2.76 GB	NCBI Assembly:<br>https://www.ncbi.nlm.nih.gov/assembly	2019-04-24

C) RNA datasets

Dataset	Number of sequences	Size	Source	Date
SILVA 132 LSURef [63]	198,843	610 MB	Silva database: https://ftp.arb-silva.de/release_132/Exports/ SILVA_132_LSURef_tax_silva.fasta.gz	2017-12-11
SILVA 132 SSURef Nr99 [63]	695,171	1.11 GB	Silva database: https://ftp.arb-silva.de/release_132/Exports/ SILVA_132_SSURef_Nr99_tax_silva.fasta.gz	2017-12-11
SILVA 132 SSURef [63]	2,090,668	3.28 GB	Silva database: https://ftp.arb-silva.de/release_132/Exports/ SILVA_132_SSURef_tax_silva.fasta.gz	2017-12-11

D) Multiple DNA sequence alignments

Dataset	Number of sequences	Size	Source	Date
UCSC hg38 7way knownCanonical-exonNuc [64]	1,470,154	340 MB	UCSC: https://hgdownload.soe.ucsc.edu/ goldenPath/hg38/multiz7way/alignments/ knownCanonical.exonNuc.fa.gz	2014-06-06
UCSC hg38 20way knownCanonical-exonNuc [64]	4,211,940	969 MB	UCSC: https://hgdownload.soe.ucsc.edu/ goldenPath/hg38/multiz20way/alignments/ knownCanonical.exonNuc.fa.gz	2015-06-30

E) Protein datasets

Dataset	Number of sequences	Size	Source	Date
PDB [65]	109,914	67.6 MB	PDB database FTP: ftp://ftp.ncbi.nih.gov/blast/db/FASTA/pdbaa.gz	2019-04-09
Homo sapiens GRCh38 [66]	105,961	73.2 MB	NCBI FTP: ftp://ftp.ensembl.org/ pub/release-96/fasta/homo_sapiens/pep/ Homo_sapiens.GRCh38.pep.all.fa.gz	2019-03-12
NCBI Virus RefSeq Protein [61]	373,332	122 MB	NCBI Virus: https://www.ncbi.nlm.nih.gov/labs/virus/vssi/	2020-05-10
UniProtKB Reviewed (Swiss-Prot) [67]	560,118	277 MB	UniProt FTP: ftp://ftp.uniprot.org/ pub/databases/uniprot/current_release/ knowledgebase/complete/uniprot_sprot.fasta.gz	2019-04-02

Click here to access/download;Manuscript;Reply-to-reviewers.pdf

Reply to reviewers

We sincerely appreciate a thorough review by the two reviewers. Our replies are below.

Reviewer #1:

This article describes a benchmark for FASTA data that includes online material with a very high potential to be used by the genomic/proteomic data compression community. The benchmark is wide, balanced, and fair. The online tool for visualization of the benchmark is efficiently implemented and easy to follow. The benchmark includes a good set of tools. In general, the work reflects a high knowledge of the tools and bioinformatics background. However, some concerns first need to be addressed before entering in a much more detailed review mode.

Thank you for taking time to review our work in detail and for the kind comment.

Major concerns:

There are many compressors for many purposes. Choosing a compressor depends on the purpose. These purposes are not limited to fast decompression of good representations, namely to fast data transfer or integration with other tools. For example, long-term storage removes the importance of fast decompression and increases the importance over the compression ratio. The same can be seen for compressors that aim to approximate the Kolmogorov complexity, namely for genomic or proteomic analysis (phylogenomics, authentication, motif localization, rearrangements, among many others). Here, the importance in only at the efficiency of the compressor side using affordable (usually high) computational time and RAM.

Developing efficient genomic/proteomic compressors is also a methodology to improve unsupervised algorithms for data mining or machine learning. An example of this can be seen in the Hutter prize (http://prize.hutter1.net/), a half-million-dollar prize where compressors can spend up to 10GB of RAM and 100 hours to compress 1 GB of data. A version of the PAQ9 algorithm, which is comparatively a very "slow" program, is currently the state-of-the-art. Therefore, centering somewhat the results in NAF (which is perhaps the best industry-oriented FASTA compressor) and limiting the conclusions to the fastest decompression algorithms according to somewhat good compression capabilities does not entirely represent the field of genomic/proteomic data compression. This because FASTA data is already in post-processed state [semi-assembled (contig, scaffold), or assembled], unlike FASTQ. This exclusivity would make sense in FASTQ. Therefore, these notions and wider conclusions would make the manuscript stronger.

Thank you for your detailed comment. We agree that there are many purposes for compression. This is why our benchmark includes 17 performance measures, including compression ratio. The users of our benchmark are free to consider measures that are most relevant to their application. In the manuscript, we tried to repeatedly emphasize the diversity of applications of our benchmark, because we believe that this benchmark should be useful for a broad variety of compressor uses.

In our study, we consider an application of compressors for actual data compression, with the main goals of conserving storage, network and computation resources required for managing large amounts of data. We believe that many compressor users (ourselves included) working with large biological datasets may benefit from a detailed investigation of compressor performances, such as what we offer in the current benchmark.

We do not explicitly address related topics such as "approximating the Kolmogorov complexity", "phylogenomics, authentication, motif localization, rearrangements", "unsupervised algorithms for data mining or machine learning". Involving such topics is currently outside of the scope of our work. We'd like to keep our manuscript focused and avoid confusing the readers, considering that the issue is already complex, and considering the broad data collected and summarized in our benchmark.

We certainly strongly support scenarios prioritizing compression strength over any other considerations. In fact, majority of the specialized sequence compressors (with few exceptions such as GTZ and DSRC) tend to prioritize compression strength and neglect speed. We have spent substantial efforts and computation resources benchmarking such compressors, because we believe they should be fairly represented, even though we ourselves don't have much use for them.

Regarding the mentioned very slow PAQ9, currently we already include several closely related compressors: cmix (arguably, a state of the art in compression strength for general-purpose data compression), zpaq (descendant from the PAQ family of algorithms), and zpipe (somewhat redundant piping variant of zpaq, although a bit older code). We are open to including more of such compressors in the future. However, long computation time required by some of such compressors means that they may be benchmarked only on smaller datasets, such as in the case of cmix, which is only benchmarked on datasets smaller than 10 MB.

Regarding "centering somewhat the results in NAF". We removed any mentions of NAF from the "Conclusion" section of the manuscript. We still mention NAF along with other top performing compressors in the "Benchmark" section. We are not aware of any of our results that are "centered in NAF".

We believe the revised version of the text is more neutral.

I also missed some protein sequence compressors, namely the recent protein compressor AC [AC: A Compression Tool for Amino Acid Sequences (https://link.springer.com/article/10.1007/s12539-019-00322-1)]. Sometimes, these are lost in a keyword search. A chain on amino acids can make a protein, therefore, the authors will find protein compressors defined as amino acid sequence compressors. The AC disadvantages: only for protein sequences (not FASTA), slower and, currently, RAM increases according to the redundancy and size of the sequence (but easily it can be adapted to a cache-hash).

Thank you very much for the suggestion. We have added AC to the benchmark.

Suggestion:

Given the current times, perhaps a very important dataset to add to the benchmark would be the whole viral database from the NCBI (FASTA format). It can be easily obtained from here: https://www.ncbi.nlm.nih.gov/labs/virus/vssi

Thank you for the suggestion. We have added two datasets from the NCBI Virus datasets that you mention. One is a 122 MB protein dataset "NCBI Virus RefSeq Protein", another is a 482 MB DNA dataset "NCBI Virus Complete Nucleotide Human".

Minor:

From 1993 to 2020 there are 27 years, therefore, the longevity of special-purpose compressors is 27-year-old. Biocompress was already available in 1992, before the publication on the DCC (in march of 1993, after review). Therefore, it could also be 28, although 27 is a safe date.

I'm not completely sure where this comment applies, as we don't specifically discuss longevity of special-purpose compressors. However, we mention longevity of gzip, which, coincidentally, was also first released in 1993. As gzip's Wikipedia article (https://en.wikipedia.org/wiki/Gzip) mentions: "Initial release 31 October 1992; 27 years ago". Thus, we updated the number to 27.

Please, improve the format of the figures and tables.

We improved figures and tables (as far as we saw a space for improvement).

Some of the bullets have a final ".", others don't. Please, pick one and use the same format.

Thank you, we changed the lists to a consistent format. Namely, we use final "." in those bulleted lists where each entry is a complete sentence. In other bulleted lists, where entries are just items of the list, we don't use final ".".

Reviewer #2:

General:

The article is well constructed and has a good explanation of the use cases for different measurement criteria, such as archival, database retrieval, one-time transfers and memory usage.

There is good attention to detail with specifying the exact versions and commit hashes of each tool, the parameters used (and their processing scripts), and references for downloading each data set. This aids reproducibility and importantly aids the use of this benchmark framework for future software authors.

Probably this article came out of the analysis for "naf", by the same authors, demonstrating that nibble-packing plus zstd is an unexpectedly strong contender. However a benchmarking framework is a valid and useful piece of work in its own right. To this end, the authors not only provide the results and a useful website, but also the tools used for producing it permitting future tools to be validated against the same data sets using the same methods. This greatly improves the value of this work.

Specifics:

1. The abstract is good. The assertion that most sequence datasets use gzip is valid, if disappointing. I checked the EMBL sequence archive, UniProt/SwissProt and NCBI's RefSeq, all of which are gzipped.

The findings / conclusion part are also good, stressing the benchmark framework and presentation rather than recommending specific tools which seems appropriate.

Language throughout is good.

Thank you very much for the time you spent reviewing our work and for encouraging comments.

2. The scope needs to be clearly spelt out.

Specifically it is targeting genomic sequence datasets (eg the aforementioned EMBL sequence databank) and not DNA sequencing reads, hence no quality values either. This is interesting as it's a little bit of a different focus from several other benchmarks.

It's also excluding reference based compression tools (eg GRS, GReEn, RLZ, CRAM). The line has to be drawn somewhere so I fully understand this, but the scope of what the article covers as well as what it doesn't cover should be more explicit.

Thank you for the suggestion. We have clarified the scope in the "Scope, compressors and test data" section (previously named "Compressors and test data").

3. Mentioning "DNA alignments" is a bit ambiguous as most people now think of output from an aligner such as bwa - ie SAM format. The format being used here is the earlier style of dash-padded sequence sets. Please clarify this distinction. I'm not sure what the proper term is, but I think "multiple sequence alignment" covers it.

Thank you for the suggestion. We have changed all mentions of alignment data to use "multiple sequence alignment" wording.

4. It is a little unclear precisely which data is being compressed.

Obviously quality values are not as mention is made to adapting fastq compressors to the task. How about reference names? Other ancillary data after the reference name (oh how I loathe FASTA for that ill-defined mess). Is it purely sequence being evaluated, or the entire FASTA file? Do tools have to be case sensitive? Do they need to cope with ambiguity codes?

The wrapper scripts cope with some of these things, but it is unclear if this is simply for purposes of testing the compression worked e.g. given the lack of support for lower case, or whether this information is actually being included in the evaluation and added as a side-channel for tools that don't support it natively. If so, how is that done?

Looking at the wrapper scripts it appears these other types of data get written to separate files and compressed with zstd. This needs documenting in the paper itself, along with an explanation of whether the size of those ancillary files is added to the compressed size, and also whether the time taken is included. (I am assuming yes, but please be explicit.)

Thank you for the suggestion. Indeed this was not clearly explained. We added long explanation in the "Methods" section, under "Streaming mode" and "FASTA format compatibility" headers.

Each compressor has to losslessly compress the entire full-featured FASTA file, including sequence names, case sensitivity and ambiguity codes. All compressors that lack native support for this, receive it via our wrappers. As you correctly assumed, the size of ancillary files, as well as time spent on pre-processing the FASTA stream and extracting these side channels (as well as adding them back during decompression) is counted as part of the total measurement.

Fortunately our wrappers are really fast and don't impact the results much for most compressors. However, all non-trivial wrappers (which means implementing anything more than streaming support) are benchmarked in "wrapper-only" mode and their results are included in benchmark database. Also fortunately those extra files are usually very small and compress well, so they don't impact overall compression rate much.

While admittedly not perfect, this seemed like the only viable strategy that would allow to compare the diverse array of compressors (each doing their own thing), and at the same time to have them doing a useful task (as opposed to compressing a raw stream of ACGT).

5. Tool selection.

There are various fastq compression tools not benchmarked, including but not limited to FQSqueezer, Minicom, Orcom and FaStore. Are these planned? This is hinted at with "our study is not a one-off benchmark, but marks the start of a project where we will continue to add compressors and test data".

However this is somewhat of a never ending task, as is alluded to with "Since it's impractical to benchmark every existing compressor, we will continue to only benchmark compressors selected based on their performance, quality and usefulness for sequence compression".

If there are specific reasons why some tools were not evaluated then perhaps this should be mentioned on the website under rejected tools along with a reason (eg for speed, robustness, reordering of data).

Thank you for a good suggestion. Indeed some compressors are still missing in benchmark, each with their own reason. We've been keeping notes about all such potential additions, so it makes perfect sense to share those notes on the website. We now added the "Missing Compressors" page to the website, accessible from the "Compressors" page. Direct link: http://kirr.dyndns.org/sequence-compression-benchmark/?page=Missing-Compressors .

I believe the benchmark is currently reasonably thorough, but there will always be more compressors to test.

Regarding the mentioned tools:

- FQSqueezer - has been added to the benchmark.
- Minicom - has been added to the benchmark.
- ORCOM - seems to always re-order the reads, making it incompatible with our conditions.
- FaStore - seems to always re-order the reads, making it incompatible with our conditions.

I have to add that testing FASTQ compressors on FASTA data (via adding constant quality) is mainly of theoretical interest and probably has little practical value.

FASTQ compressors are usually designed under a FASTQ-specific set of assumptions, such as: "all reads are very short", "all reads are of same length", "order of reads does not matter", "all reads are sampled from underlying genome with substantial coverage". These assumptions don't hold in typical FASTA data and in our benchmark. So the results we obtain for FASTQ compressors may not transfer well to their performance on actual data they are designed for.

We added a mention of this to the "Scope" section on the "About" page on the website.

Still it's interesting to see how different approaches and compressors handle genomes and other FASTA datasets, so we will probably continue to benchmark FASTQ compressors.

6. Wrapper scripts/tools.

How much time is in processing vs the actual tool? For example bsc.pl $cmd is little more than running bsc, while Quip's has 5 components piped together before piping into quip itself. Is the quip tool the bottleneck here and therefore the speed of the other bits irrelevant? I see most are in C, so it's possibly minimal impact, but it is hard to judge. If the impact is minimal, then it's probably best to acknowledge that it was measured and found to be insignificant.

Following this comment, we now also discuss this in the "FASTA format compatibility" part of the "Methods" section. In case when wrappers add anything other than streaming support, we

benchmarked the "wrapper-only" runs, so that such runs can be compared with complete "wrapper+compressor" runs. This allows us to see how much of the time is consumed by the wrapper.

In most cases the impact is minimal. There are few cases where wrappers significantly impact compression or decompression speed. Such cases occur when 2 conditions overlap: 1) Compressor is very fast. 2) Compressor requires extensive data preprocessing. Notable examples are 2bit and DSRC.

This can be seen by including both the compressor and its wrapper in a scatterplot produced on benchmark website. We added links to such analyses to the "Examples" page on the website.

Was CPU (user+system) time measured at all? If so then the ratio of wall clock to CPU time is a good indication of whether the pipeline is causing stalls or not.

Only total wall clock time was measured. I agree this could be interesting, but I don't expect much stalls. Could be interesting to try it some time.

One problem with such measurements is that I found that they influence speed of the fastest compressors. This is why, for example, memory use and speed are measured separately, using different runs of the same compressor.

7. Using fastq-from-sequence may mean that some tools has timings that aren't entirely comparable. While still a valid time for that tool, it's not indicative of the time for the sequence-only portion of that tool.

Yes, exactly. This is an inevitable consequence of testing FASTQ compressors on FASTA data, and it will remain until we add actual FASTQ data. Currently we are not sure whether we will be able to do it, but it's a possibility we consider for the future.

I don't think there is much you can do to mitigate this bar rewriting other peoples code, so realistically it's just something that could be presented as a warning.

We added an explanation and a warning in the new "FASTQ Compressors" part of the "Methods" section. We also added corresponding warning in the "Scope" section on the "About" page on the website.

8. Be explicit as to the license on your software. Some had a license declaration (public domain) but not all.

Thanks, we specified a license (public domain) on the "Wrappers" page of the website.

9. A minor typographical: "[A] wide variety of charts can be produced..."

Thanks, fixed.

Thank you for your work.

We sincerely appreciate your valuable comments on this manuscript.

 Click here to access/download;Manuscript;Cover-Letter.pdf

Kirill Kryukov, PhD
Department of Molecular Life Science,
Tokai University School of Medicine,
Shimokasuya 143, Isehara, Kanagawa 259-1193, Japan
Tel: +81-80-5107-3251
E-mail: kkryukov@gmail.com

June 1, 2020

Dear *GigaScience* Editor,

I am submitting a revision of a Technical Note titled "Sequence Compression Benchmark (SCB) database - a comprehensive evaluation of reference-free compressors for FASTA-formatted sequences" by Kirill Kryukov, Mahoko Takahashi Ueda, So Nakagawa, and Tadashi Imanishi.

We appreciate detailed comments by the two reviewers. We improved the benchmark and manuscript based on their comments. In particular, we added protein-specific compressor "AC" and two viral sequence datasets, as suggested by reviewer #1. We also added 2 compressors recommended by reviewer #2.

During submission process, the GigaScience EditorialManager website fails to process our supplementary file "Supplementary-Code.zip", therefore, could you please attach it to the submission separately? We upload it here:

http://kirill-kryukov.com/study/temp/Gigascience/Supplementary-Code.zip

This manuscript has been seen and approved by all authors. We have read and understood your journal’s policies, and we believe that neither the manuscript nor the study violates any of these. There are no conflicts of interest to declare.

Sincerely,

Kirill Kryukov, PhD
Department of Molecular Life Science,
Tokai University School of Medicine

Figure 1

Click here to access/download;Figure;Fig1.pdf

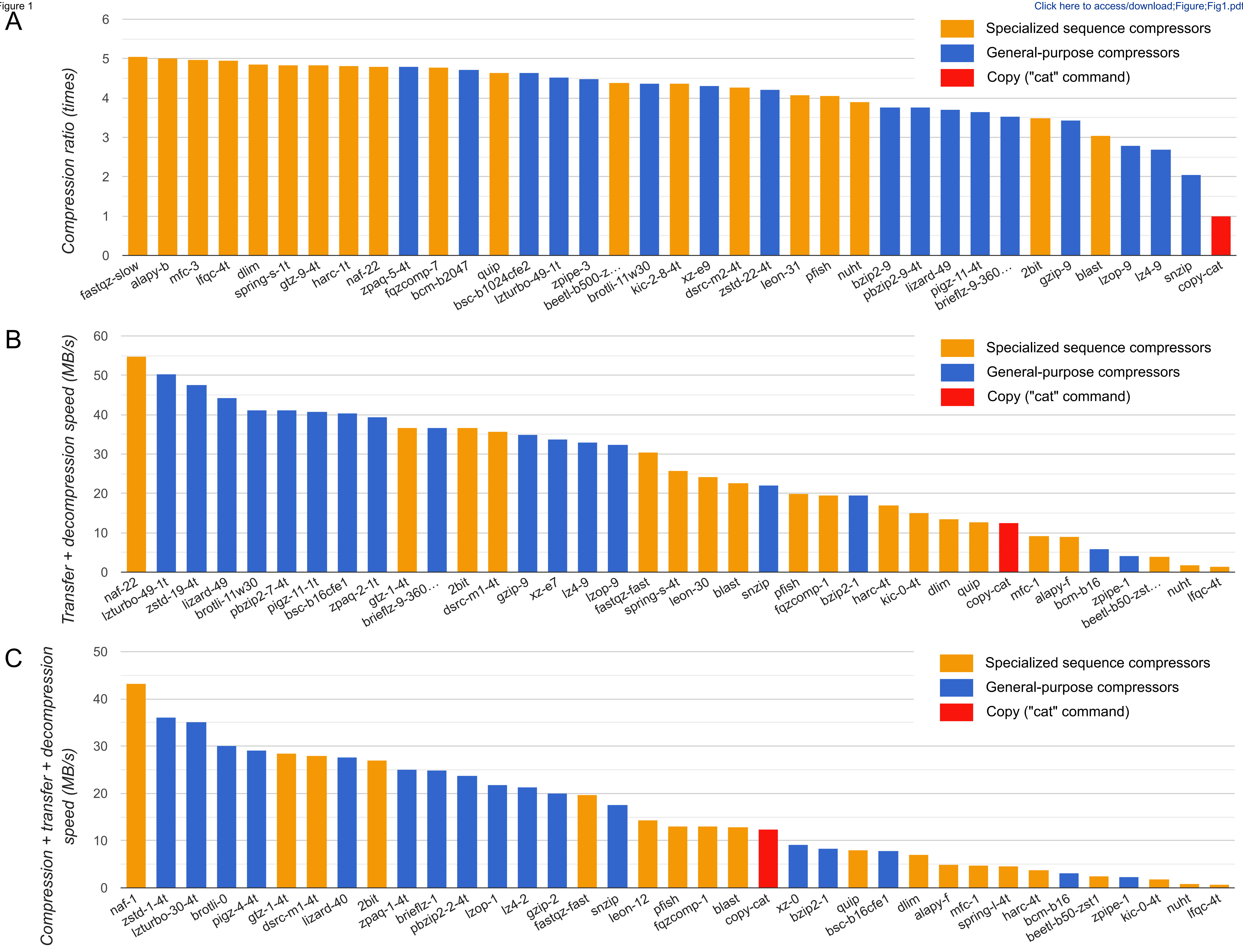

Figure 2

Click here to access/download;Figure;Fig2.pdf

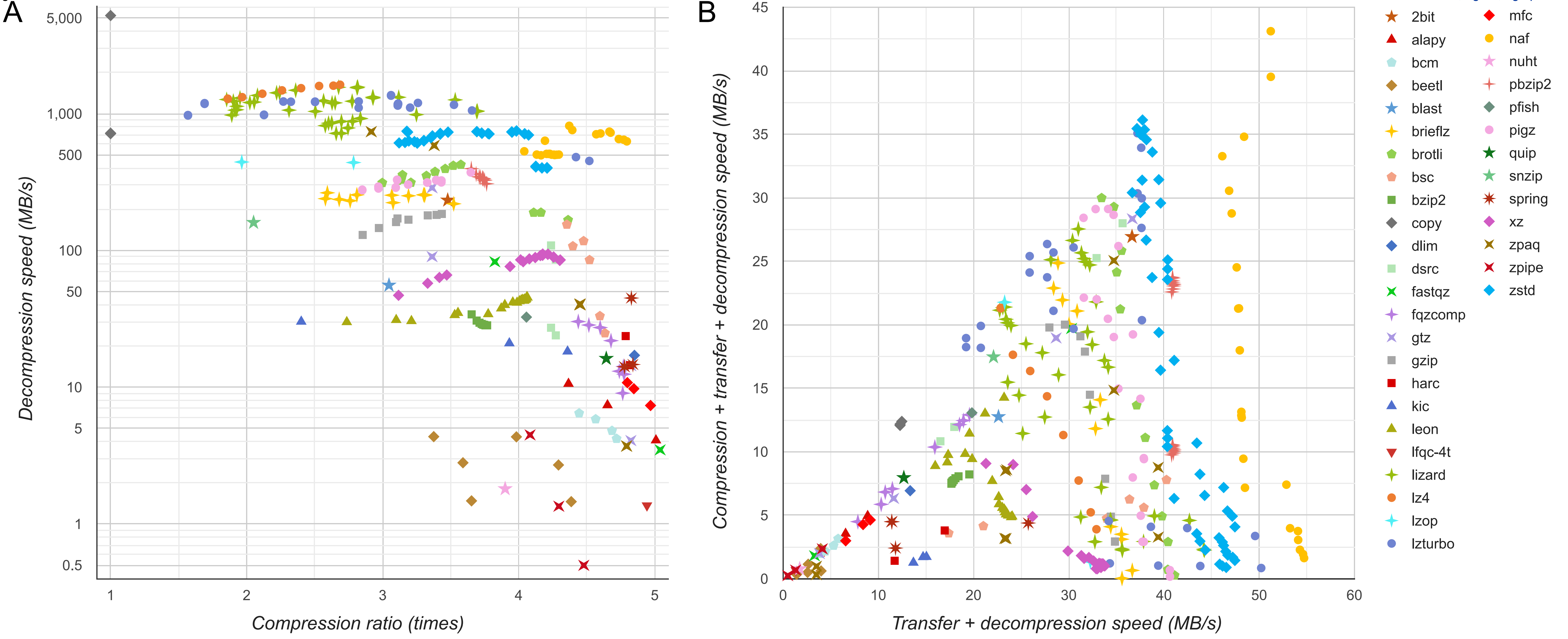

Figure 3

Click here to access/download;Figure;Fig3.pdf

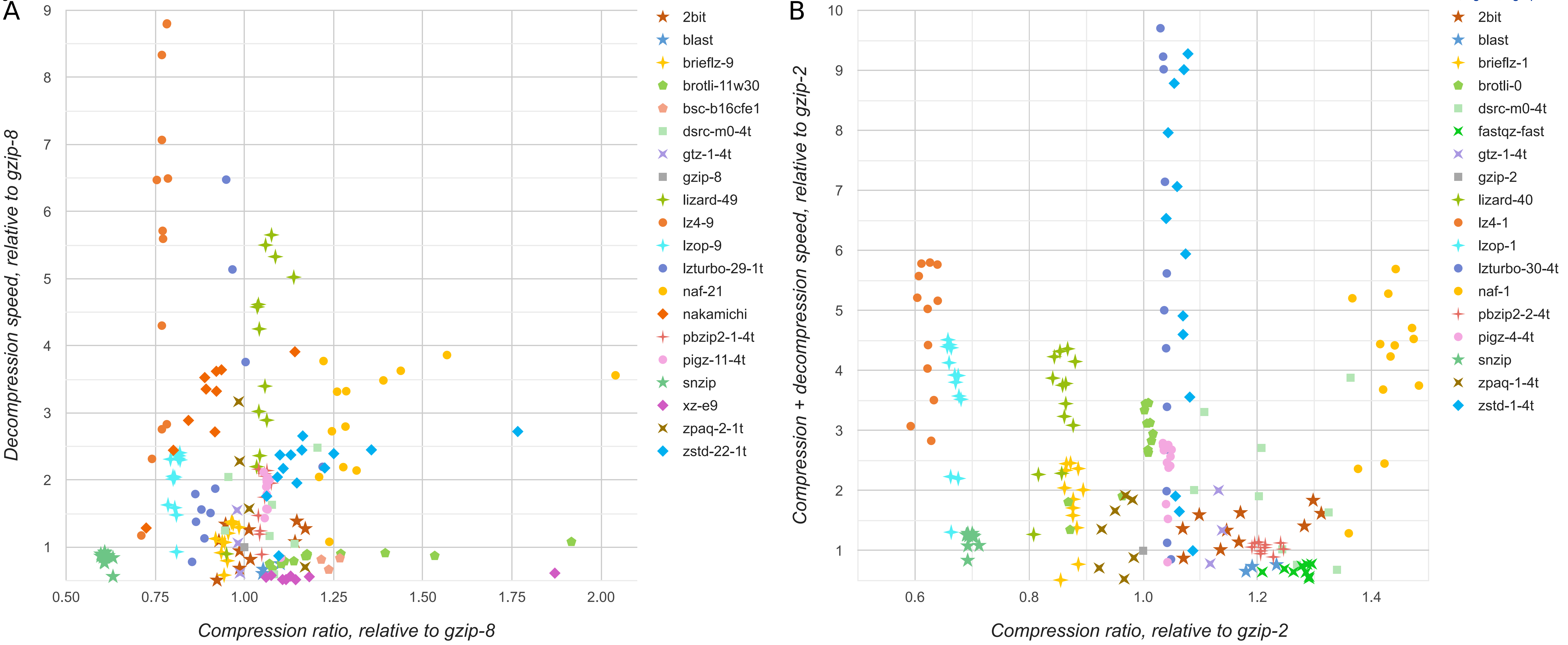

Figure 4

Click here to access/download;Figure;Fig4.pdf

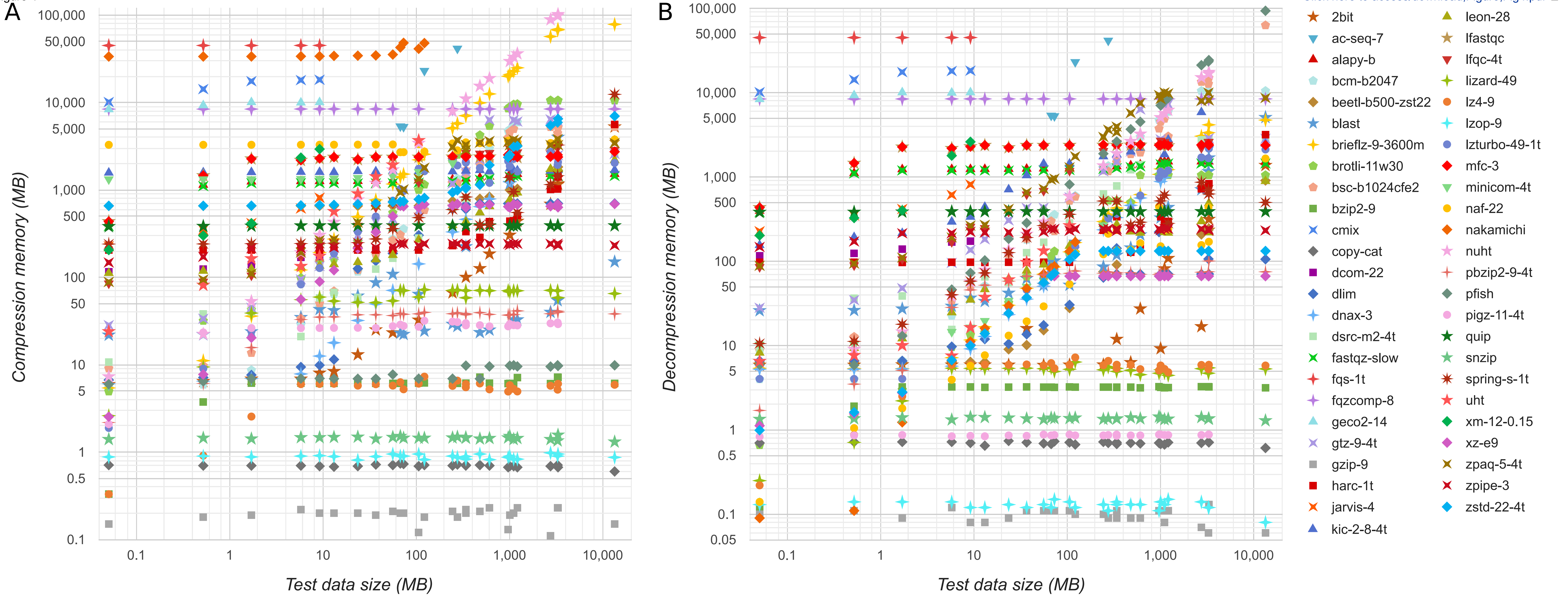

Supplementary Data

Click here to access/download
Supplementary Material
Supplementary-Data.csv

Supplementary Code Link

Click here to access/download
Supplementary Material
Supplementary-Code-Link.txt
