# Supplementary material for: Sequence Compression Benchmark (SCB) database—A comprehensive evaluation of reference-free compressors for FASTA-formatted sequences
Source: Gigascience. 2020 Jul 6;9(7):giaa072. doi: 10.1093/gigascience/giaa072 (PMC7336184; doi:10.1093/gigascience/giaa072)
Supplement: giaa072_GIGA-D-19-00442_Revision_2 [file giaa072_giga-d-19-00442_revision_2.pdf]

GigaScience

Sequence Compression Benchmark (SCB) database — a comprehensive evaluation of reference-free compressors for FASTA-formatted sequences

--Manuscript Draft--

**Manuscript Number:**	GIGA-D-19-00442R2
**Full Title:**	Sequence Compression Benchmark (SCB) database — a comprehensive evaluation of reference-free compressors for FASTA-formatted sequences
**Article Type:**	Technical Note
**Funding Information:**	School of Medicine, Tokai University — Dr. Kirill Kryukov<br>KAKENHI Grants-in-Aid for Scientific Research on Innovative Areas (16H06429) — Dr. So Nakagawa<br>KAKENHI Grants-in-Aid for Scientific Research on Innovative Areas (16K21723) — Dr. So Nakagawa<br>KAKENHI Grants-in-Aid for Scientific Research on Innovative Areas (19H04843) — Dr. So Nakagawa<br>Takeda Science Foundation — Dr. Tadashi Imanishi<br>KAKENHI Grants-in-Aid for Scientific Research (C) (20K06612) — Dr. Kirill Kryukov
**Abstract:**	Background. Nearly all molecular sequence databases currently use gzip for data compression. Ongoing rapid accumulation of stored data calls for more efficient compression tool. Although numerous compressors exist, both specialized and general-purpose, choosing one of them was difficult because no comprehensive analysis of their comparative advantages for sequence compression was available.<br>Findings. We systematically benchmarked 430 settings of 48 compressors (including 29 specialized sequence compressors and 19 general-purpose compressors) on representative FASTA-formatted datasets of DNA, RNA and protein sequences. Each compressor was evaluated on 17 performance measures, including compression strength, as well as time and memory required for compression and decompression. We used 27 test datasets including individual genomes of various sizes, DNA and RNA datasets, and standard protein datasets. We summarized the results as the Sequence Compression Benchmark database (SCB database, http://kirr.dyndns.org/sequence-compression-benchmark/) that allows building custom visualizations for selected subsets of benchmark results.<br>Conclusion. We found that modern compressors offer large improvement in compactness and speed compared to gzip. Our benchmark allows comparing compressors and their settings using a variety of performance measures, offering the opportunity to select the optimal compressor based on the data type and usage scenario specific to particular application.
**Corresponding Author:**	Kirill Kryukov, Ph.D.<br><br>JAPAN
**Corresponding Author Secondary Information:**	
**Corresponding Author's Institution:**	
**Corresponding Author's Secondary Institution:**	
**First Author:**	Kirill Kryukov, Ph.D.
**First Author Secondary Information:**	
**Order of Authors:**	Kirill Kryukov, Ph.D.<br>Mahoko Takahashi Ueda, Ph.D.<br>So Nakagawa, Ph.D.

Powered by Editorial Manager® and ProduXion Manager® from Aries Systems Corporation

	Tadashi Imanishi, Ph.D.
**Order of Authors Secondary Information:**	
**Response to Reviewers:**	Dear Dr. Zauner,<br><br>Thank you very much for the quick response! I did all the requested changes. My replies are below.<br><br>Sincerely,<br>Kirill<br><br><br>> Before we proceed to acceptance, please consider the (very minor) follow-up comments of reviewer 1, below. Our publisher OUP will provide some copy-editing, but I think the reviewer is right that it would he helpful if you go over the manuscript again to correct typos, grammatical mistakes etc.<br><br>Thank you for suggestion. I did all changes suggested by reviewer 1. I also went over the manuscript again and corrected some more typos and improved wording and grammar in a few cases.<br><br><br>> Please also add a section "Availability of supporting source code and requirements" to share details about your benchmarking script and other supporting code. If the code is not hosted in a repository, the GigaScience GitHub repository is also available for this purpose.<br><br>> List the following:<br><br>> Project name: e.g. My bioinformatics project<br>> Project home page: e.g. https://github.com/ISA-tools<br>> Operating system(s): e.g. Platform independent<br>> Programming language: e.g. Java<br>> Other requirements: e.g. Java 1.3.1 or higher, Tomcat 4.0 or higher<br>> License: e.g. GNU GPL, FreeBSD etc.<br>> RRID: if applicable, e.g. RRID: SCR_014986<br><br>> The code needs to be under an Open Source Initiative approved license (see https://opensource.org/licenses).<br><br>I uploaded all code used for this study on GitHub: https://github.com/KirillKryukov/scb. I changed the manuscript text accordingly. I also removed the now redundant Supplementary Code from submission.<br><br>All code is in Public Domain.<br><br><br>> Also before acceptance, our data curators will contact you to prepare an archival copy of code (and supporting data) for our repository GigaDB. Please include a citation to your GigaDB dataset (including the DOI link) to your reference list, and cite this in the data availability section.<br><br>> Please follow this example format for the reference:<br><br>> [xx] Author1 N, Author2 N, AuthorX N. Supporting data for "Title of your manuscript". GigaScience Database. 2020. http://dx.doi.orgxxxxxxxxxxxx<br><br>> (You will get the doi link form our curators)<br><br>I added reference [68] according to this advice.<br><br><br>> In the "availability" section, please also include a sentence to refer to this GigaDB set. For example, write: "An archival copy of scripts [and supporting data] is also

	available via the GigaScience database GigaDB [xx]."<br><br>I changed the text according to this suggestion.<br><br><br>> Reviewer reports:<br><br>> Reviewer #1: The authors have addressed all of my concerns. Thank you for addressing all.<br>> The amount of time and dedication applied to this work is impressive. Thank you for your contribution.<br>> Regarding the 27-years, I made a mistake while I was reading (please, ignore that comment).<br>> Regarding the text, I would recommend a minor review in order to improve the description (for example, using an online editor program).<br>> Some of the possible changes are:<br>> * thorougly -> thoroughly;<br>> * general purpose -> general-purpose;<br>> * X axis -> x-axis;<br>> * offer large improvement -> offer a large improvement (similar characteristics exist in several places of the text);<br>> * also, some of the parts of the text (not very often) are somewhat dense and, hence, they could be simplified.<br><br>Thanks, fixed all these typos and went through the text trying to improve the text again.
**Additional Information:**	
**Question**	**Response**
Are you submitting this manuscript to a special series or article collection?	No
**Experimental design and statistics**<br><br><br>Full details of the experimental design and statistical methods used should be given in the Methods section, as detailed in our Minimum Standards Reporting Checklist. Information essential to interpreting the data presented should be made available in the figure legends.<br><br><br>Have you included all the information requested in your manuscript?	Yes
**Resources**<br><br><br>A description of all resources used, including antibodies, cell lines, animals and software tools, with enough information to allow them to be uniquely identified, should be included in the Methods section. Authors are strongly encouraged to cite Research Resource Identifiers (RRIDs) for antibodies, model	Yes

organisms and tools, where possible.<br><br>Have you included the information requested as detailed in our Minimum Standards Reporting Checklist?	
**Availability of data and materials**<br><br>All datasets and code on which the conclusions of the paper rely must be either included in your submission or deposited in publicly available repositories (where available and ethically appropriate), referencing such data using a unique identifier in the references and in the "Availability of Data and Materials" section of your manuscript.<br><br>Have you have met the above requirement as detailed in our Minimum Standards Reporting Checklist?	Yes

Powered by Editorial Manager® and ProduXion Manager® from Aries Systems Corporation

Manuscript Click here to access/download;Manuscript;Text.pdf

Sequence Compression Benchmark (SCB) database — a comprehensive evaluation of reference-free compressors for FASTA-formatted sequences

Kirill Kryukov*, Mahoko Takahashi Ueda, So Nakagawa, Tadashi Imanishi

Department of Molecular Life Science, Tokai University School of Medicine,

Isehara, Kanagawa 259-1193, Japan.

*Correspondence: kkryukov@gmail.com

Abstract

Background. Nearly all molecular sequence databases currently use gzip for data compression. Ongoing rapid accumulation of stored data calls for more efficient compression tool. Although numerous compressors exist, both specialized and general-purpose, choosing one of them was difficult because no comprehensive analysis of their comparative advantages for sequence compression was available.

Findings. We systematically benchmarked 430 settings of 48 compressors (including 29 specialized sequence compressors and 19 general-purpose compressors) on representative FASTA-formatted datasets of DNA, RNA and protein sequences. Each compressor was evaluated on 17 performance measures, including compression strength, as well as time and memory required for compression and decompression. We used 27 test datasets including individual genomes of various sizes, DNA and RNA datasets, and standard protein datasets. We summarized the results as the Sequence Compression Benchmark database (SCB database, http://kirr.dyndns.org/sequence-compression-benchmark/) that allows building custom visualizations for selected subsets of benchmark results.

Conclusion. We found that modern compressors offer a large improvement in compactness and speed compared to gzip. Our benchmark allows comparing compressors and their settings using a variety of performance measures, offering the opportunity to select the optimal compressor based on the data type and usage scenario specific to a particular application.

Keywords: compression; benchmark; DNA; RNA; protein; genome; sequence; database.

Background

Molecular sequence databases store and distribute DNA, RNA and protein sequences as compressed FASTA-formatted files. Biological sequence compression was first proposed in 1986 [1] and the first practical compressor was made in 1993 [2]. A lively field emerged that produced a stream of methods, algorithms, and software tools for sequence compression [3,4]. However, despite this activity, currently nearly all databases universally depend on gzip for compressing FASTA-formatted sequence data. This incredible longevity of the 27-year-old compressor probably owes to multiple factors, including conservatism of database operators, wide availability of gzip, and its generally acceptable performance. Through all these years the amount of stored sequence data kept growing steadily [5], increasing the load on database operators, users, storage systems and network infrastructure. However, someone thinking to replace gzip invariably faces the questions: which of the numerous available compressors to choose? And will the resulting gains be even worth the trouble of switching?

Previous attempts at answering these questions are limited by testing too few compressors and by using restricted test data [6-11]. In addition, all of these studies provide results in form of tables, with no graphical outputs, which makes the interpretation difficult. Existing benchmarks with useful visualization such as Squash [12], are limited to general-purpose compressors.

The variety of available specialized and general-purpose compressors is overwhelming. At the same time the field was lacking a thorough investigation of comparative merits of these compressors for sequence data. Therefore we set out to benchmark all available practically useful compressors on a variety of relevant sequence data. Specifically, we focused on the common task of compressing DNA, RNA and protein sequences, stored in FASTA format, without using reference sequence. The benchmark results are available in the Sequence Compression Benchmark database (SCB database, http://kirr.dyndns.org/sequence-compression-benchmark/).

Scope, compressors and test data

We considered the common scenario of archiving, transferring and working with large datasets of biological sequences. In this study we did not investigate compression of raw sequencing data in FASTQ

format, which was previously thoroughly reviewed in [11]. Instead we focused on typical FASTA-formatted datasets, which includes individual genomes and single gene sets. Consequently we also did not consider referential compression, but only reference-free compression, which is typically used for such data. We evaluated standalone compression tools (rather than libraries), working under Linux OS on a modern workstation PC. In this study we only consider lossless compression.

We tested all DNA sequence compressors that are available and functional in 2020: dnaX [13], XM [14], DELIMINATE [15], Pufferfish [16], DNA-COMPACT [17], MFCompress [18], UHT [19], GeCo [20], GeCo2 [21], JARVIS [22], NAF [23], and NUHT [24]. We also included the relatively compact among homology search database formats: BLAST [25] and 2bit - a database format of BLAT [26].

Since compressors designed for FASTQ data can be trivially adopted for FASTA-formatted inputs, we also included a comprehensive array of compressors designed primarily or specifically for FASTQ data: BEETL [27], Quip [28], fastqz [10], fqzcomp [10], DSRC 2 [29], Leon [30], LFQC [31], KIC [32], ALAPY [33], GTX.Zip [34], HARC [35], LFastqC [36], SPRING [37], Minicom [38], and FQSqueezer [39]. We also included AC - a compressor designed exclusively for protein sequences [40]. We also tested a comprehensive array of general-purpose compressors: bcm [41], brieflz[42], brotli [43], bsc [44], bzip2 [45], cmix [46], gzip [47], lizard [48], lz4 [49], lzop [50], lzturbo [51], nakamichi [52], pbzip2 [53], pigz [54], snzip [55], xz [56], zpaq [57], zpipe [57] and zstd [58]. See Table 1 for the list of compressors we used.

For the test data, we selected a variety of commonly used sequence datasets in FASTA format: (1) Individual genomes of various sizes, as examples of non-repetitive data [59,60]; (2) DNA and RNA datasets, such as collections of mitochondrial genomes, influenza virus sequences [60,61,62,59], 16S rRNA gene sequences [63], and genomic multiple DNA sequence alignments [64]; (3) Standard protein datasets [65,66,61,67]. Individual genomes are less repetitive, while other datasets are more repetitive. In total we used 27 test datasets. See Table 2 for the list of test data. All test data is available at the GigaDB repository [68].

Benchmark

We benchmarked each compressor on every test dataset, except in cases of incompatibility (e.g., DNA compressors cannot compress protein data) or excessive time requirement (some compressors are so

slow that they would take weeks on larger datasets). For compressors with adjustable compression level, we tested the relevant range of levels. We tested both 1 and 4-thread variants of compressors that support multi-threading. In total, we used 430 settings of 48 compressors. We also included the non-compressing "cat" command as control. For compressors using non-trivial wrappers, we also benchmarked the wrappers.

Currently many sequence analysis tools accept gzip-compressed files as input. Switching to another compressor may require either adding support of new format to those tools, or passing the data in uncompressed form. The latter solution can be achieved with the help of Unix pipes, if both the compressor and the analysis tool support streaming mode. Therefore, we benchmarked all compressors in streaming mode (streaming uncompressed data in both compression and decompression).

For each combination of compressor setting and test dataset we recorded compressed size, compression time, decompression time, peak compression memory and peak decompression memory. The details of the method and raw benchmark data are available in the Methods section and Supplementary Data, respectively. We share benchmark results at the online SCB database: http://kirr.dyndns.org/sequence-compression-benchmark/. All benchmark code is available at https://github.com/KirillKryukov/scb.

The choice of measure for evaluating compressor performance depends on a prospective application. For a long-term data storage, compactness may be the single most important criterion. For a public sequence database, the key measure is how long time it takes from initiating the download of compressed files until accessing the decompressed data. This time consists of transfer time plus decompression time (TD-Time). Corresponding transfer-decompression speed (TD-Speed) is computed as Original Size / TD-Time. In this use case, compression time is relatively unimportant, since compression happens only once, while transfer and decompression times affect every user of the database. For a one-time data transfer, all three steps of compression, transfer and decompression are timed (CTD-Time), and used for computing the resulting overall speed (CTD-Speed).

A total of 17 measures, including the above-mentioned ones, are available in our results data (See Methods for the list of measures). Any of these measures can be used for selecting the best setting of each compressor and for sorting the list of compressors. These measures can be then shown in a table and visualized in form of column charts and scatterplots. This allows tailoring the output to answer specific questions, such as what compressor is better at compressing particular kind of data, or which setting of each

compressor performs best at particular task. The link speed that is used for estimating transfer times is configurable. The default speed of 100 Mbit/sec corresponds to the common speed of a fixed broadband internet connection.

Fig.1 compares the performance of best settings of 36 compressors on human genome. It shows that specialized sequence compressors achieve excellent compression ratio on this genome. However, when total TD-Speed or CTD-Speed is considered (measures that are important in practical applications), most sequence compressors fall behind the general-purpose ones. The best compressors for this dataset in terms of compression ratio, TD-Speed and CTD-Speed are "fastqz-slow", "naf-22" and "naf-1", respectively (numbers in each compressor name indicate compression level and other settings). Interestingly, the non-compressing "cat" command used as a control, while naturally showing at the last place on compression ratio (Fig.1A), is not the slowest in terms of TD-Speed and CTD-Speed (Figs.1B and 1C, respectively). In case of CTD-Speed, for example, it means that some compressors are so slow that their compression + transfer + decompression time turns out to be longer than the time required for transferring raw uncompressed data (using a particular link speed, in this case 100 Mbit/sec).

Fig.2 compares all compressor settings on the same data (human genome). Fig.2A shows that the strongest compressors often provide a very low decompression speed (shown using logarithmic scale due to the enormous range of values), which means that quick data transfer (resulting from strong compression) offered by those compressors is offset by significant waiting time required for decompressing the data. Fig.2B shows TD-Speed plotted against the CTD-Speed. Similar figures can be constructed for other data and performance measures on the SCB database website.

Visualizing results from multiple test datasets simultaneously is possible, with or without aggregation of data. With aggregation, the numbers will be summed or averaged, and a single measurement will be shown for each setting of each compressor. Without aggregation, the results of each compressor setting will be shown separately on each dataset. Since the resulting number of data points can be huge, in such case it is useful to request only the best setting of each compressor to be shown. The criteria for choosing the best setting is selectable among the 17 measurements. In case of a column chart, any of the 17 measures can be used for ordering the shown compressors, independently of the measure used for selecting best version, and independently of the measure actually shown in the chart.

One useful capability of the SCB database is showing measurements relative to the specified compressor (and setting). This allows selecting a reference compressor and comparing the other compressors to this reference. For example, we can compare compressors to gzip as shown on Fig.3. In this example, we compare only the best settings of each compressor, selected using specific measures (transfer+decompression speed and compression+transfer+decompression speed on Figs.3A and 3B, respectively). We also used a fixed scale to show only range above 0.5 on both axes, which means that only performances that are at least half as good as gzip on both axes as shown. In this example, we can see that some compressors improve compactness and some improve speed compared to gzip, but few compressors improve both at the same time, such as lizard, naf, pigz, pbzip, and zstd.

It is important to be aware of the memory requirements when choosing a compressor (Fig.4). In these charts we plotted data size on the x-axis, and disabled aggregation. This allows seeing how much memory a particular compressor used on each test dataset. As this example shows, memory requirement reaches saturation point for most compressors. On the other hand, some compressors have unbounded growth of consumed memory, which makes them unusable for large data. Interestingly, gzip apparently has the smallest memory footprint, which may be one of the reasons for its popularity. Most compressors can function on a typical desktop hardware, but some require larger memory, which is important to consider when choosing a compressor that will be run by the consumers of distributed data.

A wide variety of charts can be produced on the benchmark website by selecting specific combinations of test data, compressors, and performance measures. At any point the currently visualized data can be obtained in textual form using Table output option. Also, all charts can be downloaded in SVG format.

Conclusions

Our benchmark reveals complex relationship between compressors and between their settings, based on various measures. We found that continued use of gzip is usually far from an optimal choice. Transitioning from gzip to a better compressor brings significant gains for genome and protein data, and is especially beneficial with repetitive DNA/RNA datasets. The optimal choice of compressor depends on many factors, including properties of the data to be compressed (such as sequence type, data size, and

amount of redundancy), relative importance of compression strength, compression speed and decompression speed for particular use scenario, as well as amount of memory available on data machines used for compression and decompression. Our benchmark allows comparing compressors on individual performance metrics, as well as on their combinations.

The Sequence Compression Benchmark (SCB) database will help in navigating the complex landscape of sequence data compression. With dozens of compressors available, making an informed choice is not an easy task and requires careful analysis of the project requirements, data type and compressor capabilities. Our benchmark is the first resource providing a detailed practical evaluation of various compressors on a wide range of molecular sequence datasets. Using the SCB database, users can analyze compressor performances on a variety of metrics, and construct custom reports for answering project-specific questions.

In contrast to previous studies that showed their results in static tables, our project is dynamic in two important senses: (1) the result tables and charts can be dynamically constructed for a custom selection of test data, compressors, and measured performance numbers, and (2) our study is not a one-off benchmark, but marks the start of a project where we will continue to add compressors and test data.

Making an informed choice of a compressor with the help of our benchmark will lead to increased compactness of sequence databases, with shorter time required for downloading and decompressing. This will reduce the load on network and storage infrastructure, and increase the speed and efficiency in biological and medical research.

Declarations

Availability of data and material

All benchmark data is available at the online SCB database:

http://kirr.dyndns.org/sequence-compression-benchmark/

An archival copy of benchmark data is also available via the GigaScience database GigaDB [68].

Availability of supporting source code and requirements

All code used for conducting the benchmark is available at the SCB GitHub repository.

Project name: Sequence Compression Benchmark

Project home page: https://github.com/KirillKryukov/scb

Operating system(s): Linux

Programming language: Perl

Other requirements: None

License: Public Domain

Competing interests

The authors declare no competing interests.

Funding

This work was supported by the 2019 Tokai University School of Medicine Research Aid (to KK), JSPS KAKENHI Grants-in-Aid for Scientific Research (C) (20K06612 to KK) and Scientific Research on Innovative Areas (16H06429, 16K21723, 19H04843 to SN), and Takeda Science Foundation (to TI).

Authors' contributions

KK conceived the study idea and implemented the benchmark. SN provided benchmark hardware. KK, MTU, SN and TI interpreted the data and wrote the manuscript. KK and MTU prepared figures and tables. All authors read and approved the final manuscript.

Acknowledgements

Not applicable.

References

1. Walker JR, Willett P. Compression of nucleic acid and protein sequence data. *Comput. Appl. Biosci.* 1986;2(2):89-93.
2. Grumbach S, Tahi F. Compression of DNA sequences. *Data Compression Conference*, Snowbird, Utah, IEEE Computer Society. 1993. p. 340-50. doi:10.1109/DCC.1993.253115.
3. Deorowicz S, Grabowski S. Data compression for sequencing data. *Algorithms for Molecular Biology*. 2013;8:25. doi:10.1186/1748-7188-8-25.

4. Hernaez M, Pavlichin D, Weissman T, Ochoa I. Genomic Data Compression. *Annual Review of Biomedical Data Science*. 2019;2:19-37. doi:10.1146/annurev-biodatasci-072018-021229.
5. Karsch-Mizrachi I, Takagi T, Cochrane G. The international nucleotide sequence database collaboration. *Nucleic Acids Res.* 2018;46(Database issue):D48–D51. doi:10.1093/nar/gkx1097.
6. Zhu Z, Zhang Y, Ji Z, He S, Yang X. High-throughput DNA sequence data compression. *Brief. Bioinform.* 2013; 16(1):1-15. doi:10.1093/bib/bbt087.
7. Hosseini M, Pratas D, Pinho AJ. A Survey on Data Compression Methods for Biological Sequences. *Information*. 2016;7(4):56. doi:10.3390/info7040056.
8. Sardaraz M, Tahir M. Advances in high throughput DNA sequence data compression. *J. Bioinform. Comput. Biol*. 2016;14(3):1630002. doi:10.1142/S0219720016300021.
9. Biji CL, Achuthsankar SN. Benchmark Dataset for Whole Genome Sequence Compression. *IEEE/ACM Trans. Comput. Biol. Bioinform.* 2017;14(6):1228-36. doi:10.1109/TCBB.2016.2568186.
10. Bonfield JK, Mahoney MV. Compression of FASTQ and SAM Format Sequencing Data. *PLoS One*. 2013;8(3): e59190, doi:10.1371/journal.pone.0059190.
11. Numanagic I, Bonfield JK, Hach F, Voges J, Ostermann J, Alberti C, Mattavelli M. Comparison of high-throughput sequencing data compression tools. *Nature Methods*. 2016;13(12):1005-8, doi:10.1038/nmeth.4037.
12. Squash Compression Benchmark. 2015. https://quixdb.github.io/squash-benchmark/. Accessed July 15, 2019.
13. Manzini G, Rastero M. A simple and fast DNA compressor. *Software - Practice and Experience.* 2004;34:1397-411, doi:10.1002/spe.619.
14. Cao MD, Dix TI, Allison L. Mears C. A simple statistical algorithm for biological sequence compression. *Data Compression Conference*. DCC '07, Snowbird, UT, IEEE Computer Society. 2007. p. 43-52. doi:10.1109/DCC.2007.7.
15. Mohammed MH, Dutta A, Bose T, Chadaram S, Mande SS. DELIMINATE — a fast and efficient method for loss-less compression of genomic sequences. *Bioinformatics*. 2012;28:2527–29. doi:10.1093/bioinformatics/bts467.
16. Pufferfish. 2012. https://github.com/alexholehouse/pufferfish. Accessed May 23, 2019.

17. Li P, Wang S, Kim J, Xiong H, Ohno-Machado L, Jiang X. DNA-COMPACT: DNA COMpression Based on a Pattern-Aware Contextual Modeling Technique. *PLoS ONE.* 2013;8(11):e80377. doi:10.1371/journal.pone.0080377.
18. Pinho AJ, Pratas D. MFCompress: a compression tool for FASTA and multi-FASTA data. *Bioinformatics*. 2014;30:117-8. doi:10.1093/bioinformatics/btt594.
19. Al-Okaily A, Almarri B, Al Yami S, Huang CH. Toward a Better Compression for DNA Sequences Using Huffman Encoding. *J. Comp. Biol.* 2017;24(4):280–8. doi:10.1089/cmb.2016.0151.
20. Pratas D, Pinho AJ, Ferreira PJSG. Efficient compression of genomic sequences. *Data Compression Conference,* DCC-2016, Snowbird, Utah, IEEE Computer Society. 2016. p.231-240. doi: 10.1109/DCC.2016.60.
21. Pratas D, Hosseini M, Pinho AJ. GeCo2: An Optimized Tool for Lossless Compression and Analysis of DNA Sequences. *Practical Applications of Computational Biology and Bioinformatics, 13th International Conference,* PACBB 2019, Advances in Intelligent Systems and Computing, vol 1005, Springer, Cham, 2019a. p.137-145. doi: 10.1007/978-3-030-23873-5_17.
22. Pratas D, Hosseini M, Silva J, Pinho AJ. A Reference-Free Lossless Compression Algorithm for DNA Sequences Using a Competitive Prediction of Two Classes of Weighted Models. *Entropy,* 2019b;21:1074. doi:10.3390/e21111074.
23. Kryukov K, Ueda MT, Nakagawa S, Imanishi T. Nucleotide Archival Format (NAF) enables efficient lossless reference-free compression of DNA sequences. *Bioinformatics.* 2019;35(19):3826-28. doi:10.1093/bioinformatics/btz144.
24. Alyami S, Huang CH. Nongreedy Unbalanced Huffman Tree Compressor for Single and Multifasta Files. Journal of Computational Biology. 2019; 26(0):1-9. doi:10.1089/cmb.2019.0249.
25. Altschul SF, Gish W, Miller W, Myers EW, Lipman DJ. Basic local alignment search tool. *J. Mol. Biol.* 1990;215(3):403-10. doi:10.1016/S0022-2836(05)80360-2.
26. Kent WJ. BLAT - The BLAST-Like Alignment Tool. *Genome Research.* 2002;12(4):656-64. doi:10.1101/gr.229202.

27. Bauer MJ, Cox AJ, Rosone G. Lightweight BWT Construction for Very Large String Collections. *Combinatorial Pattern Matching 2011*, proceedings of the CPM 2011, 2011. p.219-231. doi:10.1007/978-3-642-21458-5_20.
28. Jones DC, Ruzzo WL, Peng X, Katze MG. Compression of next-generation sequencing reads aided by highly efficient de novo assembly. *Nucleic Acids Research.* 2012;40(22):e171. doi:10.1093/nar/gks754.
29. Roguski L, Deorowicz S. DSRC 2—Industry-oriented compression of FASTQ files. *Bioinformatics.* 2014; 30(15):2213-5. doi:10.1093/bioinformatics/btu208.
30. Benoit G, Lemaitre C, Lavenier D, Drezen E, Dayris T, Uricaru R, Rizk G. Reference-free compression of high throughput sequencing data with a probabilistic de Bruijn graph. *BMC Bioinformatics*. 2015;16:288. doi:10.1186/s12859-015-0709-7.
31. Nicolae M, Pathak S, Rajasekaran S. LFQC: a lossless compression algorithm for FASTQ files. *Bioinformatics.* 2015;31(20):3276-81. doi:10.1093/bioinformatics/btv384.
32. Zhang Y, Patel K, Endrawis T, Bowers A, Sun Y. A FASTQ compressor based on integer-mapped k-mer indexing for biologist. *Gene.* 2016;579(1):75-81. doi:10.1016/j.gene.2015.12.053.
33. ALAPY 2017. http://alapy.com/services/alapy-compressor/. Accessed December 2, 2019.
34. Xing Y, Li G, Wang Z, Feng B, Song Z, Wu C. GTZ: a fast compression and cloud transmission tool optimized for FASTQ files. *BMC Bioinformatics.* 2017;18(Suppl 16):549. doi:10.1186/s12859-017-1973-5.
35. Chandak S, Tatwawadi K, Weissman T. Compression of genomic sequencing reads via hash-based reordering: algorithm and analysis. *Bioinformatics.* 2018;34(4):558-67. doi:10.1093/bioinformatics/btx639.
36. Al Yami S, Huang CH. LFastqC: A lossless non-reference-based FASTQ compressor. PLoS One. 2019;14(11):e0224806, doi:10.1371/journal.pone.0224806.
37. Chandak S, Tatwawadi K, Ochoa I, Hernaez M, Weissman T. SPRING: a next-generation compressor for FASTQ data. Bioinformatics. 2019;35(15):2674-6. doi:10.1093/bioinformatics/bty1015.
38. Liu Y, Yu Z, Dinger ME, Li J. Index suffix-prefix overlaps by (w, k)-minimizer to generate long contigs for reads compression. Bioinformatics. 2019. 35(12):2066-2074, doi:10.1093/bioinformatics/bty936.

39. Deorowicz S. FQSqueezer: k-mer-based compression of sequencing data. Scientific Reports. 2020;10:578. doi:10.1038/s41598-020-57452-6.
40. Hosseini M, Pratas D, Pinho AJ. AC: A Compression Tool for Amino Acid Sequences. Interdisciplinary Sciences: Computational Life Sciences. 2019;11:68-76. doi:10.1007/s12539-019-00322-1.
41. BCM. https://github.com/encode84/bcm. Accessed June 6 2019.
42. BriefLZ - small fast Lempel-Ziv. https://github.com/jibsen/brieflz. Accessed May 12 2020.
43. Alakuijala J, Szabadka Z. Brotli Compressed Data Format. RFC 7932. 2016. Accessed April 14 2019.
44. libbsc. https://github.com/IlyaGrebnov/libbsc. Accessed June 22 2019.
45. bzip2. https://www.sourceware.org/bzip2/. Accessed January 20 2019.
46. cmix. https://github.com/byronknoll/cmix. Accessed April 25 2019.
47. GNU Gzip. https://www.gnu.org/software/gzip/. Accessed November 8 2019.
48. Lizard - efficient compression with very fast decompression. https://github.com/inikep/lizard. Accessed June 16 2019.
49. LZ4 - Extremely fast compression. https://github.com/lz4/lz4. Accessed April 25 2019.
50. Lzop. 2017. https://www.lzop.org/. Accessed December 6 2018.
51. LzTurbo - World's fastest compressor. https://sites.google.com/site/powturbo/. Accessed February 11 2019.
52. Nakamichi. http://www.sanmayce.com/Nakamichi/index.html. Accessed May 12 2020.
53. pbzip2. https://launchpad.net/pbzip2/. Accessed April 26 2019.
54. pigz. https://zlib.net/pigz/. Accessed April 26 2019.
55. Snzip, a compression/decompression tool based on snappy. https://github.com/kubo/snzip. Accessed November 11 2018.
56. XZ Utils. https://tukaani.org/xz/. Accessed December 17 2018.
57. ZPAQ Incremental Journaling Backup Utility and Archiver. http://www.mattmahoney.net/dc/zpaq.html. Accessed November 7 2018.
58. Zstandard - Fast real-time compression algorithm. https://github.com/facebook/zstd. Accessed May 22 2020.

59. Clark K, Karsch-Mizrachi I, Lipman DJ, Ostell J, Sayers EW. GenBank. *Nucleic Acids Res.* 2016;44(D1):D67–D72. doi:10.1093/nar/gkv1276.
60. O'Leary NA, Wright MW, Brister JR, Ciufo S, Haddad D, McVeigh R, et al. Reference sequence (RefSeq) database at NCBI: current status, taxonomic expansion, and functional annotation. *Nucleic Acids Res.* 2016;44(D1):D733-45. doi:10.1093/nar/gkv1189.
61. Brister JR, Ako-Adjei D, Bao Y, Blinkova O. NCBI viral genomes resource. *Nucleic Acids Res.* 2015;43(D1):D571-7. doi:10.1093/nar/gku1207.
62. Bao Y, Bolotov P, Dernovoy D, Kiryutin B, Zaslavsky L, Tatusova T, Ostell J, Lipman D. The Influenza Virus Resource at the National Center for Biotechnology Information. *J Virol.* 2008;82(2):596-601. doi:10.1128/JVI.02005-07.
63. Quast C, Pruesse E, Yilmaz P, Gerken J, Schweer T, Yarza P, Peplies J, Glöckner FO. The SILVA ribosomal RNA gene database project: improved data processing and web-based tools. *Nucl. Acids Res.* 2013;41(D1):D590-D596. doi:10.1093/nar/gks1219.
64. Kent WJ, Sugnet CW, Furey TS, Roskin KM, Pringle TH, Zahler AM, Haussler D. The human genome browser at UCSC. *Genome Res.* 2002;12(6):996-1006. doi:10.1101/gr.229102.
65. Berman HM, Westbrook J, Feng Z, Gilliland G, Bhat TN, Weissig H, Shindyalov IN, Bourne PE. The Protein Data Bank. *Nucleic Acids Res.* 2000;28:235-42. doi:10.1093/nar/28.1.235.
66. Yates AD, Achuthan P, Akanni W, Allen J, Allen J, Alvarez-Jarreta J, et al. Ensembl 2020. *Nucleic Acids Res.* 2020;48(D1):D682–8. doi:10.1093/nar/gkz966.
67. The UniProt Consortium. UniProt: a worldwide hub of protein knowledge. *Nucleic Acids Res.* 2019;47(D1):D506-15. doi:10.1093/nar/gky1049.
68. Kryukov K, Ueda MT, Nakagawa S, Imanishi T. Supporting data for "Sequence Compression Benchmark (SCB) database — a comprehensive evaluation of reference-free compressors for FASTA-formatted sequences". GigaScience Database. 2020. http://dx.doi.orgxxxxxxxxxxxx

Methods

Benchmarked task

The task is to compress and decompress a FASTA-formatted file containing DNA, RNA or protein sequences. The process has to be lossless, i.e., decompressed data must be byte-to-byte identical to the original data. Compression and decompression are done without using any reference genome. Each compression and decompression task is executed under the Linux OS, via a command line interface. Input data for compression and output data during decompression are streamed using Unix pipes.

Only well-formed FASTA files are used in the benchmark: They must contain no empty lines and all long sequence lines have to be wrapped at the same position. Both upper- and lower-case (soft-masked) letters can be present, as well as common ambiguity codes. In multiple sequence alignments, additionally, dashes ("-") are used for indicating gaps. Each test dataset is compressed separately from other datasets.

Compressor selection

We used all specialized sequence compressors that we could find and make to work for the above specified task. For general-purpose compressors we used only the major ones, in terms of performance, historical importance, or popularity. For each compressor with configurable compression level (or other parameters related to compression strength of speed), we used the relevant range of settings, including the default.

Benchmark machine

- CPU: dual Xeon E5-2643v3 (3.4 GHz, 6 cores), hyperthreading: off
- RAM: 128 GB DDR4-2133 ECC Registered
- Storage: 4 x 2 TB SSD, in RAID 0, XFS filesystem, block size: 4096 bytes (blockdev --getbsz)
- OS: Ubuntu 18.04.1 LTS, kernel: 4.15.0
- GCC: 7.4.0

Compressor/dataset combinations that were tested

Each setting of each compressor is tested on every test dataset, except when it's difficult or impossible due to compressor limitations:

- AC is a protein-specific compressor, and was tested only on protein datasets.
- Due to their extreme slowness, these compressors are not tested on any data larger than 10 MB: cmix, DNA-COMPACT, GeCo, JARVIS, Leon, and XM.

- UHT fails on the 245 MB dataset and on larger data.
- Nakamichi was only used on data smaller than 200 MB due to its slowness and memory requirements.
- Among sequence compressors, only DELIMINATE, MFCompress and NAF support multiple sequence alignments.
- Among sequence compressors, only AC, BLAST and NAF support protein sequences.
- Some settings of XM crash and/or produce wrong decompressed output on some data - such results are not included.
- NUHT's memory requirement makes it impossible to use on 13.4 GB *Picea abies* genome.
- LFastqC fails on 2.7 GB dataset and larger data.

Benchmark process

The entire benchmark is orchestrated by a perl script. This script loads the lists of compressor settings and test data, and proceeds to test each combination that still has its measurements missing in the output directory. For each such combination (of compressor setting and test dataset), the following steps are performed:

1. Compression is performed by piping the test data into the compressor. Compressed size and compression time is recorded. For compressed formats consisting of multiple files, sizes of all files are summed together.
2. If compression time did not exceed 10 seconds, 9 more compression runs are performed, recording compression times. Compressed data from previous run is deleted before each next compression run.
3. The next set of compression runs is performed to measure peak memory consumption. This set consists of the same number of runs as in steps 1-2 (either 1 or 10 runs). That is, for fast compressors and for small data the measurement is repeated 10 times.
4. Decompression test run is performed. In this run decompressed data is piped to the "md5sum -b -" command. The resulting md5 signature is compared with that of the original file. In case of any mismatch this combination of compressor setting and dataset is disqualified and its measurements are discarded.
5. Decompression time is measured. This time decompressed data is piped to /dev/null.

6. If decompression completed within 10 seconds, 9 more decompression runs are performed and timed.
7. Peak decompression memory is measured. The number of runs is same as in steps 5-6.
8. The measurements are stored to a file. All compressed and temporary files are removed.

Measurement methods

Measuring time: Wall clock time was measured using Perl's Time::HiRes module (gettimeofday and tv_interval subroutines). The resulting time was recorded with millisecond precision.

Measuring peak memory consumption: First, each compression command was stored in a temporary shell script file. Then it was executed via GNU Time, as /usr/bin/time -v cmd.sh >output.txt. "Maximum resident set size" value was extracted from the output. 1638 was then subtracted from this value and the result was stored as peak memory measurement. 1638 is the average "Maximum resident set size" measured by GNU Time in the same way for an empty shell script.

Memory consumption and time were measured separately because measuring memory makes the task slower, especially for very fast tasks.

Collected measurements

For each combination of compressor and dataset that was tested, the following measurements were collected:

- Compressed size (in bytes)
- Compression time (in milliseconds)
- Decompression time (in milliseconds)
- Peak compression memory (in GNU Time's "Kbytes")
- Peak decompression memory (in GNU Time's "Kbytes")

In cases where 10 values are collected, the average value is used by the benchmark web-site.

Computed values

The following values were calculated based on the measured values:

- Compressed size relative to original (%) = Compressed size / Uncompressed size * 100
- Compression ratio (times) = Uncompressed size / Compressed size

- Compression speed (MB/s) = Uncompressed size in MB / Compression time
- Decompression speed (MB/s) = Uncompressed size in MB / Decompression time
- Compression + decompression time (s) = Compression time + Decompression time
- Compression + decompression speed (MB/s) = Uncompressed size in MB / (Compression time + Decompression time)
- Transfer time (s) = Uncompressed size / Link speed in B/s
- Transfer speed (MB/s) = Uncompressed size in MB / Transfer time
- Transfer + decompression time (s) = Transfer time + Decompression time
- Transfer + decompression speed (MB/s) = Uncompressed size in MB / (Transfer time + Decompression time)
- Compression + transfer + decompression time (s) = Compression time + Transfer time + Decompression time
- Compression + transfer + decompression speed (MB/s) = Uncompressed size in MB / (Compression time + Transfer time + Decompression time)

Rationale for non-constant number of runs

Variable number of runs is the only way to have both accurate measurements and large test data (under the constraints of using one test machine, and running benchmark within reasonable time).

On one hand, benchmark takes lot of time. So much that some compressors can't be even tested at all on dataset larger than 10 MB in reasonable time. Therefore repeating every measurement 10 times is impractical. Or, it would imply restricting the test data to only small datasets.

On the other hand, measurements are slightly noisy. The shorter measured time, the more noisy its measurement. Thus for very quick runs, multiple runs allow for substantial noise suppression. For longer runs it does not make much difference, because the relative error is already small with longer times.

Using a threshold of 10 seconds seems to be a reasonable compromise between suppressing noise and including larger test data (and slow compressors).

Streaming mode

For compression, each compressor was reading the input data streamed via unix pipe ("|" in the command line). For decompression, each compressor was set up to stream decompressed data via pipe. This was done to better approximate a common pattern of using compressors in a practical data analysis scenario. In an actual sequence analysis workflow, often decompressed data is piped directly into a downstream analysis command. Also, when compressing the sequences, often the data is first pre-processed with another command, which then pipes processed sequences to a compressor.

Some compressors don't implement the streaming mode, and only work with actual files. Since we have to benchmark all compressors on the same task, we added streaming mode to such compressors via wrapper scripts. For compression, a wrapper reads input data from "stdin" and writes it into a temporary file, then executes a compressor on that file, and finally deletes the file. For decompression the reverse process occurs: The wrapper script executes a decompressor, which writes the decompressed data into a temporary file; then the wrapper reads this file and streams it to "stdout", before deleting the file.

The entire process is timed for the benchmark. Normally such wrapping has minimal impact on the overall compression/decompression speed, because we use fast SSD storage, and because the actual compression and decompression takes comparatively much longer time than simply streaming the data to/from a file.

FASTA format compatibility

Many specialized compressors don't support the full-featured modern FASTA format, such as the one used in genome databases. Specifically, modern FASTA files often store masked sequence (use a mix of upper- and lower-case letters), and include ambiguity codes. The degree of completeness of FASTA support varies wildly among compressors. At one end of the spectrum, there are compressors with comprehensive support for all FASTA format features. At another end, there are compressors that only work with a string of capital ACGT and nothing else, not even sequence names or newlines. Majority of sequence compressors are somewhere in-between these two extremes.

Essentially this means that each sequence compressor performs its own task, different from that of the others. If a compressor does not need to care about upper vs lower-case letters, or about storing sequence names, it can possibly work faster. Thus comparing compressors each doing their own thing would not be fair, or very useful to the user. Since full-featured FASTA is in fact commonly used in today's databases, we

decided to require complete lossless support of full-featured FASTA files from all benchmarked compressors. In practice this means that we had to create a custom wrapper for each incomplete compressor, implementing the missing compatibility features.

A typical wrapper takes the original FASTA-formatted input and transforms it into a format acceptable by the compressor being wrapped. For instance, if a compressor only expects upper-case nucleotide codes, then the positions of upper- and lowe-case characters are extracted and saved in a separate file. The original file is converted to all upper-case, which is then fed to the compressor. The separate "mask" file (storing positions of lower-case letters) is compressed with a general-purpose compressor. Entire set of files produces in such way counts for the compressed data size measured for this particular compressor and dataset, so that the overall compression strength is comparable with that achieved by other compressors (with or without their respective wrappers). Also the total time is measured, including the time taken by all transformations and by storing/compressing the additional files.

We developed several tools for quickly processing FASTA files to extract or add various channels of information for the purpose of wrapping the incomplete compressors. We used C and optimized for speed, so that these steps have maximum speed and minimap impact on the overall compression. The wrapper scripts themselves are written in Perl. We used the fast mode of zstd ("-1") to compress the additional files, chosen because of its high speed so that it has minimal impact on measuring the speed of the wrapped compressor. As for compactness, the impact is minimal as well since the additional files are typically very small and compress well.

For all such wrapped compressors, we benchmarked not only the complete wrapped compressor, but also the "wrapper-only" mode, in which only the wrapper script is executed, but not the compressor itself. Such results are included in the benchmark under the "wrap-NAME" names. This means that it's possible to compare the speed of the entire wrapped compressor with its corresponding "wrapper-only" run, for each dataset. This allows to see how much time is used by the wrapper, and therefore how much impact the wrapper makes on the overall results.

Some of the features implemented via wrappers:

- Supporting RNA sequences for DNA-only compressors
- Supporting 'N' in DNA/RNA sequences

- Supporting IUPAC's ambiguous nucleotide codes
- Saving and restoring line lengths
- Saving and restoring sequence names
- Saving and restoring sequence mask (upper/lower case)
- Supporting FASTA-formatted input
- Supporting input with more than 1 sequence

FASTQ compressors

Several FASTQ compressors are included in the benchmark. All of them are tested using wrappers which convert FASTA sequences into their respective accepted formats. Some need only adding the artificial quality (constant "A" in most cases). Other expect only short reads or reads of identical lengths. These transformations are done in custom wrappers that we made for each FASTQ compressor. Since compression and decompression time recorded for benchmark is the total time of all steps, including wrapper processing, it means that in many cases the wrapped tool may work faster when used directly on FASTQ data. Also many FASTQ compressors are designed under additional assumptions typical for FASTQ data, for example that all reads are sampled from an underlying genome with substantial coverage (which allows meaningful assembly). These assumptions often don't hold on our FASTA-formatted benchmark datasets. Therefore the results of FASTQ compressors shown in our benchmark should not be taken as indicative of the actual performance of those compressors on FASTQ data that they were designed for.

Benchmark code availability

All scripts used for conducting the benchmark are available at the GitHub repository: https://github.com/KirillKryukov/scb. The main benchmark scrripts and configuration files are in the "benchmark" directory. All wrappers are in the "wrappers" directory. Additional tools used by the wrappers are in "seq-tools-c" and "seq-tools-perl" directories. Compression and decompression commands are listed in files "benchmark/compressors-*.txt" and "benchmark/decompressors.txt". Benchmark data is merged using the "benchmark/2-collect-results.pl" script. The resulting merged data is visualized using a server-side script in the "website" directory. The scripts are provided for reference only.

Update plan

We plan to continue maintaining Sequence Compression Benchmark. This mainly involves benchmarking new or updated compressors, when such compressors become available. Since it's impractical to benchmark every existing compressor, we will continue to only benchmark compressors selected based on their performance, quality and usefulness for sequence compression.

Figure legends

Fig. 1. Comparison of 36 compressors on human genome. The best settings of each compressor are selected based on different aspects of performance: (A) compression ratio, (B) transfer + decompression speed, and (C) compression + transfer + decompression speed. Specialized sequence compressors are shown in orange color, and general-purpose compressors are shown in blue. The copy-compressor ("cat" command), shown in red color, is included as a control. The selected settings of each compressor are shown in their names, after hyphen. Multi-threaded compressors have "-1t" or "-4t" at the end of their names to indicate the number of threads used. Test data is the 3.31 GB reference human genome (accession number GCA_000001405.28). Benchmark CPU: Intel Xeon E5-2643v3 (3.4 GHz). Link speed of 100 Mbit/s was used for estimating the transfer time.

Fig. 2. Comparison of 334 settings of 36 compressors on human genome. Each point represents a particular setting of some compressor. Panel A shows the relationship between compression ratio and decompression speed. Panel B shows the transfer + decompression speed plotted against compression + transfer + decompression speed. Test data is the 3.31 GB reference human genome (accession number GCA_000001405.28). Benchmark CPU: Intel Xeon E5-2643v3 (3.4 GHz). Link speed of 100 Mbit/s was used for estimating the transfer time.

Fig. 3. Comparison of compressor settings to gzip. Genome datasets were used as test data. Each point shows the performance of a compressor setting on specific genome test dataset. All values are shown relative to representative setting of gzip. Only performances that are at least half as good as gzip on both axes are shown. Panel A shows settings that performed best in Transfer+Decompression speed, B - settings that

performed best in Compression+Transfer+Decompression speed. Link speed of 100 Mbit/s was used for estimating the transfer time.

Fig. 4. Compressor memory consumption. The strongest setting of each compressor is shown. On the x-axis is the test data size. On the y-axis is the peak memory used by the compressor, for compression (A) and decompression (B).

Table 1. Compressor versions

A) Specialized sequence compressors

Compressor	Version
2bit	"faToTwoBit" and "twoBitToFa" binaries dated 2018-11-07
ac	AC 1.1, 2020-01-29
alapy	ALAPY 1.3.0, 2017-07-25
beetl	BEETL, commit 327cc65, 2019-11-14
blast	"convert2blastmask", "makeblastdb" and "blastdbcmd" binaries from BLAST 2.8.1+, 2018-11-26
dcom	DNA-COMPACT, latest public source 2013-08-29
dlim	DELIMINATE, version 1.3c, 2012
dnaX	dnaX 0.1.0, 2014-08-03
dsrc	DSRC 2.02, commit 5eda82c, 2015-06-04
fastqz	fastqz 1.5, commit 39b2bbc, 2012-03-15
fqs	FQSqueezer 0.1, commit 5741fc5, 2019-05-17
fqzcomp	fqzcomp 4.6, commit 96f2f61, 2019-12-02
geco	GeCo: v.2.1, 2016-12-24<br>GeCo2: v.1.1, 2019-02-02
gtz	GTX.Zip PROFESSIONAL-2.1.3-V-2020-03-18 07:11:20, binary
harc	HARC, commit cf35caf, 2019-10-04
jarvis	JARVIS v.1.1, commit d7daef5, 2019-04-30
kic	KIC binary, 0.2, 2015-11-25
leon	Leon, 1.0.0, 2016-02-27, Linux binary
lfastqc	LFastqC, commit 60e5fda, 2019-02-28, with fixes
lfqc	LFQC, commit 59f56e0, 2016-01-06
mfc	MFCompress,s1.01, 2013-09-03, 64-bit Linux binary
minicom	Minicom, commit 2360dd9, 2019-09-09
naf	NAF, 1.1.0, 2019-10-01
nuht	NUHT, commit 08a42a8, 2018-09-26, Linux binary

pfish	Pufferfish, v.1.0 alpha, 2012-04-11
quip	Quip, commit 9165bb5, 1.1.8-8-g9165bb5, 2017-12-17
spring	SPRING, commit 6536b1b, 2019-11-28
uht	UHT, binary from 2016-12-27
xm	XM (eXpert-Model), 3.0, commit 9b9ea57, 2019-01-07

B) General-purpose compressors

Compressor	Version
bcm	1.30, 2018-01-21
brieflz	1.3.0, 2020-02-15
brotli	1.0.7, 2018-10-23
bsc	3.1.0, 2016-01-01
bzip2	1.0.6, 2010-09-06
cmix	17, 2019-03-24
gzip	1.6, 2013-06-09
lizard	1.0.0, 2019-03-08
lz4	1.9.1, 2019-04-24
lzop	1.04, 2017-08-10
lzturbo	1.2, 2014-08-11
nakamichi	2020-May-09
pbzip2	1.1.13, 2015-12-18
pigz	2.4, 2017-12-26
snzip	1.0.4, 2016-10-02
xz	5.2.2, 2015-09-29
zpaq	7.15, 2016-08-17
zpipe	2.01, 2010-12-23
zstd	1.4.5, 2020-05-22

Table 2. Test datasets

A) Genome sequence datasets

Category	Organism	Accession	Size
Virus	*Gordonia phage GAL1* [60]	GCF_001884535.1	50.7 kB
Bacteria	*WS1 bacterium JGI 0000059-K21* [59]	GCA_000398605.1	522 kB
Protist	*Astrammina rara* [59]	GCA_000211355.2	1.71 MB
Fungus	*Nosema ceranae* [59]	GCA_000988165.1	5.81 MB
Protist	*Cryptosporidium parvum Iowa II* [59]	GCA_000165345.1	9.22 MB
Protist	*Spironucleus salmonicida* [59]	GCA_000497125.1	13.1 MB

Protist	*Tieghemostelium lacteum* [59]	GCA_001606155.1	23.7 MB
Fungus	*Fusarium graminearum PH-1* [60]	GCF_000240135.3	36.9 MB
Protist	*Salpingoeca rosetta* [59]	GCA_000188695.1	56.2 MB
Algae	*Chondrus crispus* [59]	GCA_000350225.2	106 MB
Algae	*Kappaphycus alvarezii* [59]	GCA_002205965.2	341 MB
Animal	*Strongylocentrotus purpuratus* [60]	GCF_000002235.4	1.01 GB
Plant	*Picea abies* [59]	GCA_900067695.1	13.4 GB

B) Other DNA datasets

Dataset	Number of sequences	Size	Source	Date
Mitochondrion [60]	9,402	245 MB	RefSeq FTP: ftp://ftp.ncbi.nlm.nih.gov/refseq/release/ mitochondrion/mitochondrion.1.1.genomic.fna.gz ftp://ftp.ncbi.nlm.nih.gov/refseq/release/ mitochondrion/mitochondrion.2.1.genomic.fna.gz	2019-03-15
NCBI Virus Complete Nucleotide Human [61]	36,745	482 MB	NCBI Virus: https://www.ncbi.nlm.nih.gov/labs/virus/vssi/	2020-05-11
Influenza [62]	700,001	1.22 GB	Influenza Virus Database: ftp://ftp.ncbi.nih.gov/genomes/INFLUENZA/ influenza.fna.gz	2019-04-27
Helicobacter [59]	108,292	2.76 GB	NCBI Assembly: https://www.ncbi.nlm.nih.gov/assembly	2019-04-24

C) RNA datasets

Dataset	Number of sequences	Size	Source	Date
SILVA 132 LSURef [63]	198,843	610 MB	Silva database: https://ftp.arb-silva.de/release_132/Exports/ SILVA_132_LSURef_tax_silva.fasta.gz	2017-12-11
SILVA 132 SSURef Nr99 [63]	695,171	1.11 GB	Silva database: https://ftp.arb-silva.de/release_132/Exports/ SILVA_132_SSURef_Nr99_tax_silva.fasta.gz	2017-12-11
SILVA 132 SSURef [63]	2,090,668	3.28 GB	Silva database: https://ftp.arb-silva.de/release_132/Exports/ SILVA_132_SSURef_tax_silva.fasta.gz	2017-12-11

D) Multiple DNA sequence alignments

Dataset	Number of sequences	Size	Source	Date
UCSC hg38 7way knownCanonical-exonNuc [64]	1,470,154	340 MB	UCSC: https://hgdownload.soe.ucsc.edu/ goldenPath/hg38/multiz7way/alignments/ knownCanonical.exonNuc.fa.gz	2014-06-06
UCSC hg38 20way knownCanonical-exonNuc [64]	4,211,940	969 MB	UCSC: https://hgdownload.soe.ucsc.edu/ goldenPath/hg38/multiz20way/alignments/ knownCanonical.exonNuc.fa.gz	2015-06-30

E) Protein datasets

Dataset	Number of sequences	Size	Source	Date

PDB [65]	109,914	67.6 MB	PDB database FTP: ftp://ftp.ncbi.nih.gov/blast/db/FASTA/pdbaa.gz	2019-04-09
Homo sapiens GRCh38 [66]	105,961	73.2 MB	NCBI FTP: ftp://ftp.ensembl.org/ pub/release-96/fasta/homo_sapiens/pep/ Homo_sapiens.GRCh38.pep.all.fa.gz	2019-03-12
NCBI Virus RefSeq Protein [61]	373,332	122 MB	NCBI Virus: https://www.ncbi.nlm.nih.gov/labs/virus/vssi/	2020-05-10
UniProtKB Reviewed (Swiss-Prot) [67]	560,118	277 MB	UniProt FTP: ftp://ftp.uniprot.org/ pub/databases/uniprot/current_release/ knowledgebase/complete/uniprot_sprot.fasta.gz	2019-04-02

Figure 1

Click here to access/download;Figure;Fig1.pdf

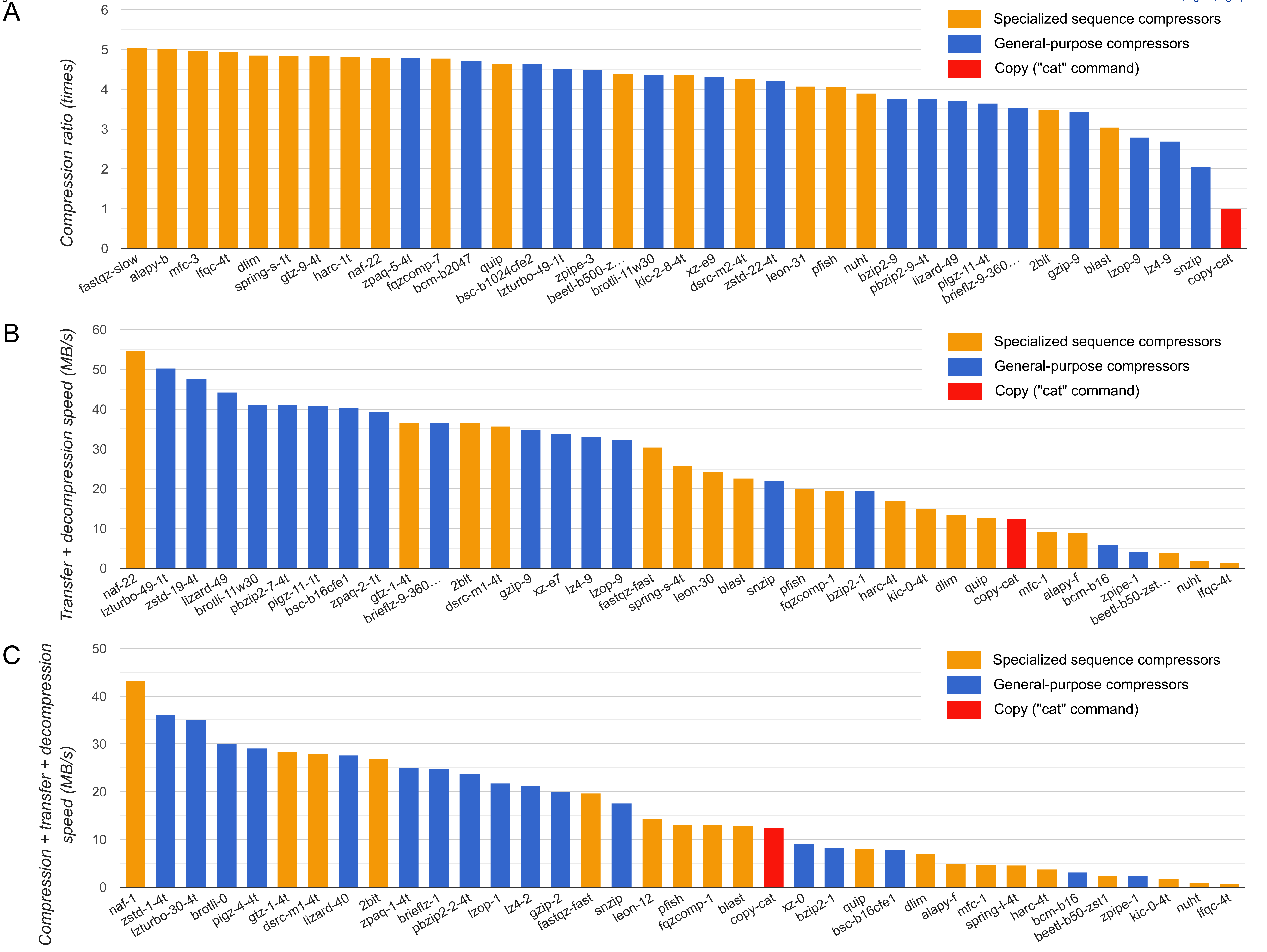

Figure 2

Click here to access/download;Figure;Fig2.pdf

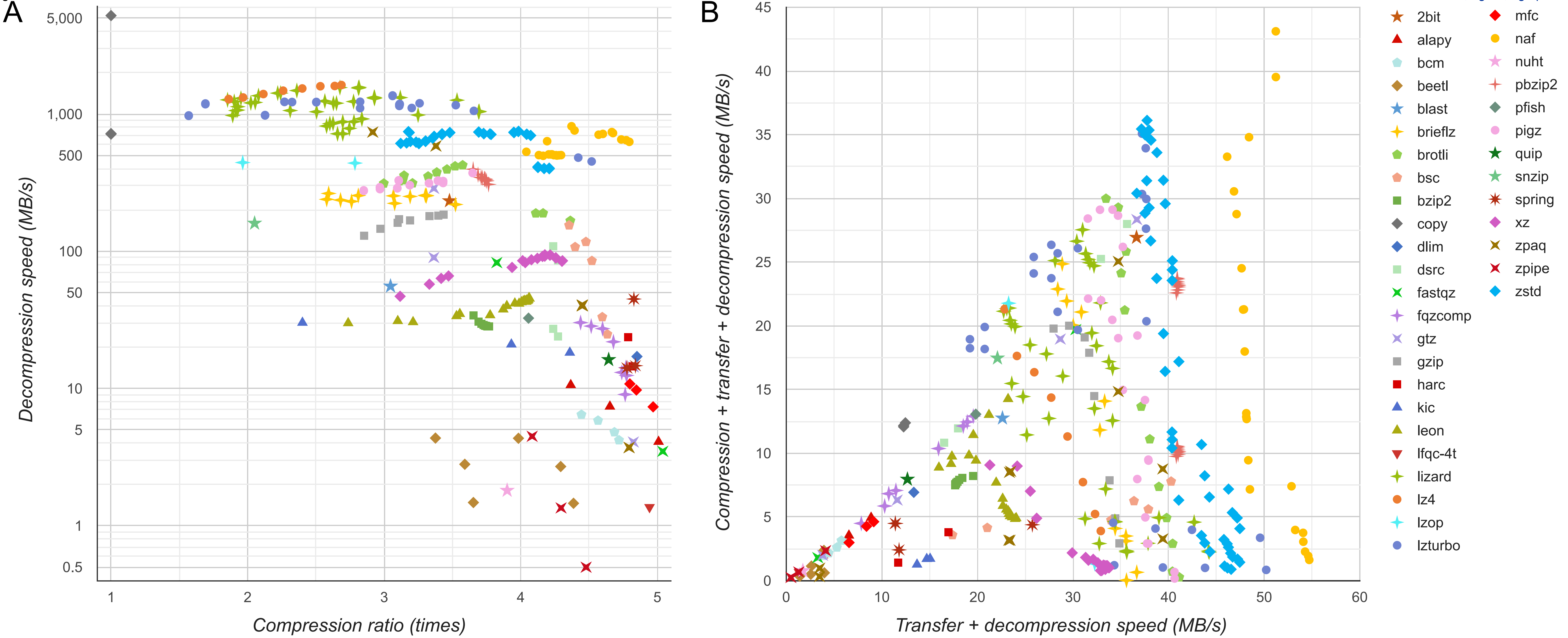

Figure 3

Click here to access/download;Figure;Fig3.pdf

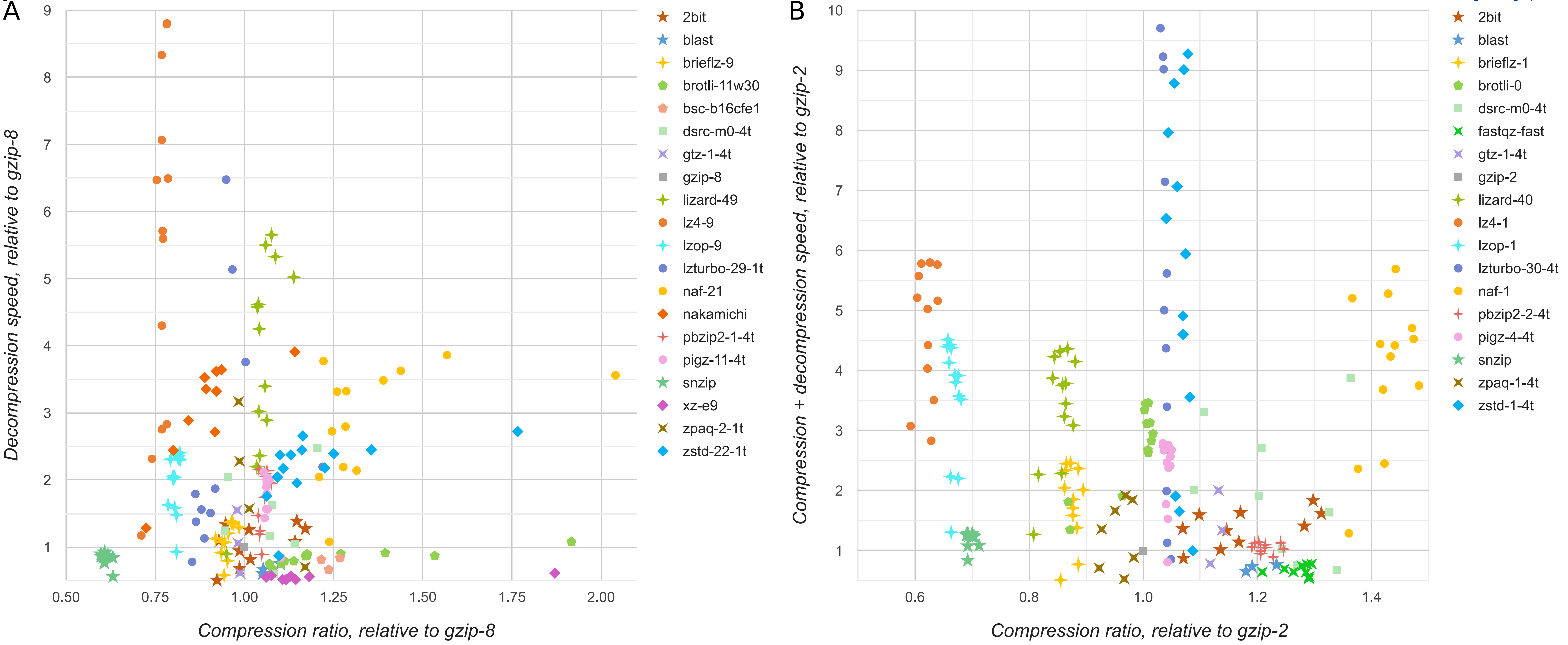

Figure 4

Click here to access/download;Figure;Fig4.pdf

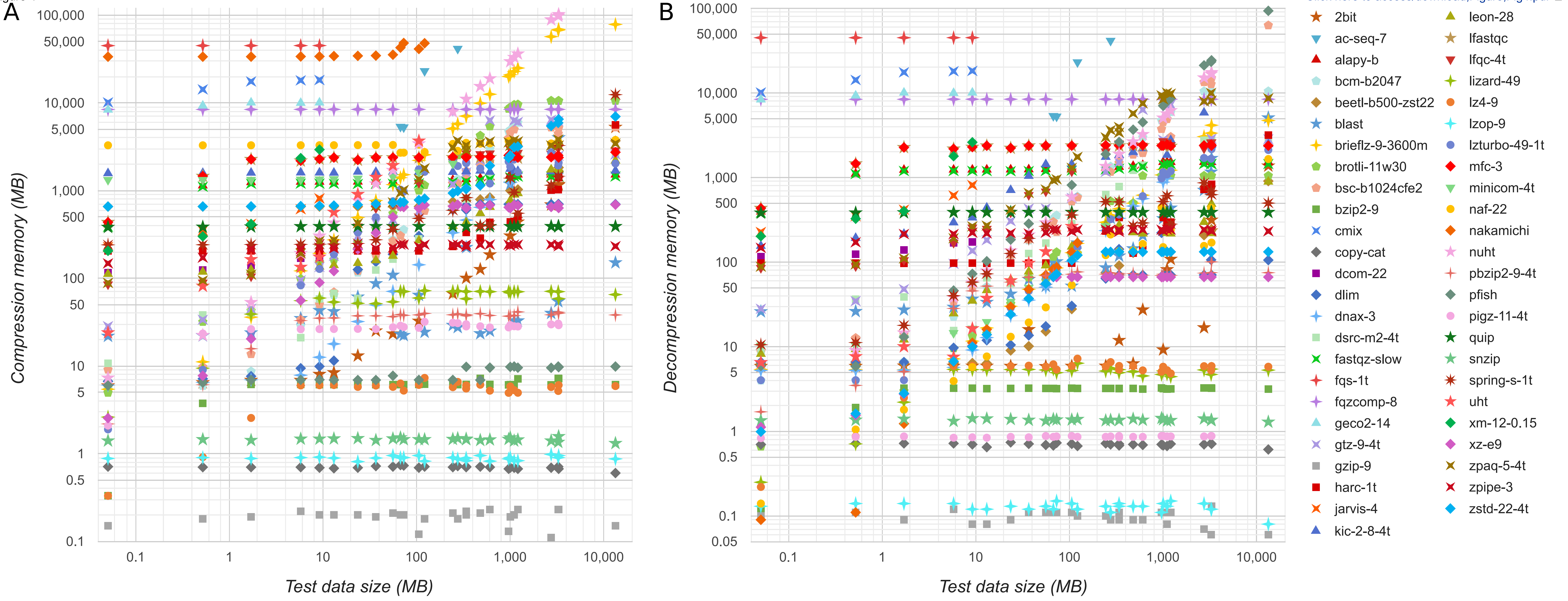

Supplementary Data

Click here to access/download
Supplementary Material
Supplementary-Data.csv
