# Supplementary material for: Sequence Compression Benchmark (SCB) database—A comprehensive evaluation of reference-free compressors for FASTA-formatted sequences
Source: Gigascience. 2020 Jul 6;9(7):giaa072. doi: 10.1093/gigascience/giaa072 (PMC7336184; doi:10.1093/gigascience/giaa072)
Supplement: giaa072_Response_to_Reviewer_Comments_Original_Submission [file giaa072_response_to_reviewer_comments_original_submission.pdf]

Author's Response To Reviewer Comments

Close

Reply to reviewers

We sincerely appreciate a thorough review by the two reviewers. Our replies are below.

> Reviewer #1:

> This article describes a benchmark for FASTA data that includes online material with a very high potential to be used by the genomic/proteomic data compression community. The benchmark is wide, balanced, and fair. The online tool for visualization of the benchmark is efficiently implemented and easy to follow. The benchmark includes a good set of tools. In general, the work reflects a high knowledge of the tools and bioinformatics background. However, some concerns first need to be addressed before entering in a much more detailed review mode.

Thank you for taking time to review our work in detail and for the kind comment.

> Major concerns:

> There are many compressors for many purposes. Choosing a compressor depends on the purpose. These purposes are not limited to fast decompression of good representations, namely to fast data transfer or integration with other tools. For example, long-term storage removes the importance of fast decompression and increases the importance over the compression ratio. The same can be seen for compressors that aim to approximate the Kolmogorov complexity, namely for genomic or proteomic analysis (phylogenomics, authentication, motif localization, rearrangements, among many others). Here, the importance in only at the efficiency of the compressor side using affordable (usually high) computational time and RAM.

> Developing efficient genomic/proteomic compressors is also a methodology to improve unsupervised algorithms for data mining or machine learning. An example of this can be seen in the Hutter prize (http://prize.hutter1.net/), a half-million-dollar prize where compressors can spend up to 10GB of RAM and 100 hours to compress 1 GB of data. A version of the PAQ9 algorithm, which is comparatively a very "slow" program, is currently the state-of-the-art. Therefore, centering somewhat the results in NAF (which is perhaps the best industry-oriented FASTA compressor) and limiting the conclusions to the fastest decompression algorithms according to somewhat good compression capabilities does not entirely represent the field of genomic/proteomic data compression. This because FASTA data is already in post-processed state [semi-assembled (contig, scaffold), or assembled], unlike FASTQ. This exclusivity would make sense in FASTQ. Therefore, these notions and wider conclusions would make the manuscript stronger.

Thank you for your detailed comment. We agree that there are many purposes for compression. This is why our benchmark includes 17 performance measures, including compression ratio. The users of our benchmark are free to consider measures that are most relevant to their application. In the manuscript, we tried to repeatedly emphasize the diversity of applications of our benchmark, because we believe that this benchmark should be useful for a broad variety of compressor uses.

In our study, we consider an application of compressors for actual data compression, with the main goals of conserving storage, network and computation resources required for managing large amounts of data. We believe that many compressor users (ourselves included) working with large biological datasets may benefit from a detailed investigation of compressor performances, such as what we offer in the current benchmark.

We do not explicitly address related topics such as "approximating the Kolmogorov complexity", "phylogenomics, authentication, motif localization, rearrangements", "unsupervised algorithms for data mining or machine learning". Involving such topics is currently outside of the scope of our work. We'd

like to keep our manuscript focused and avoid confusing the readers, considering that the issue is already complex, and considering the broad data collected and summarized in our benchmark.

We certainly strongly support scenarios prioritizing compression strength over any other considerations. In fact, majority of the specialized sequence compressors (with few exceptions such as GTZ and DSRC) tend to prioritize compression strength and neglect speed. We have spent substantial efforts and computation resources benchmarking such compressors, because we believe they should be fairly represented, even though we ourselves don't have much use for them.

Regarding the mentioned very slow PAQ9, currently we already include several closely related compressors: cmix (arguably, a state of the art in compression strength for general-purpose data compression), zpaq (descendant from the PAQ family of algorithms), and zpipe (somewhat redundant piping variant of zpaq, although a bit older code). We are open to including more of such compressors in the future. However, long computation time required by some of such compressors means that they may be benchmarked only on smaller datasets, such as in the case of cmix, which is only benchmarked on datasets smaller than 10 MB.

Regarding "centering somewhat the results in NAF". We removed any mentions of NAF from the "Conclusion" section of the manuscript. We still mention NAF along with other top performing compressors in the "Benchmark" section. We are not aware of any of our results that are "centered in NAF".

We believe the revised version of the text is more neutral.

> I also missed some protein sequence compressors, namely the recent protein compressor AC [AC: A Compression Tool for Amino Acid Sequences (https://link.springer.com/article/10.1007/s12539-019-00322-1)]. Sometimes, these are lost in a keyword search. A chain on amino acids can make a protein, therefore, the authors will find protein compressors defined as amino acid sequence compressors. The AC disadvantages: only for protein sequences (not FASTA), slower and, currently, RAM increases according to the redundancy and size of the sequence (but easily it can be adapted to a cache-hash).

Thank you very much for the suggestion. We have added AC to the benchmark.

> Suggestion:

> Given the current times, perhaps a very important dataset to add to the benchmark would be the whole viral database from the NCBI (FASTA format). It can be easily obtained from here: https://www.ncbi.nlm.nih.gov/labs/virus/vssi

Thank you for the suggestion. We have added two datasets from the NCBI Virus datasets that you mention. One is a 122 MB protein dataset "NCBI Virus RefSeq Protein", another is a 482 MB DNA dataset "NCBI Virus Complete Nucleotide Human".

> Minor:

> From 1993 to 2020 there are 27 years, therefore, the longevity of special-purpose compressors is 27-year-old. Biocompress was already available in 1992, before the publication on the DCC (in march of 1993, after review). Therefore, it could also be 28, although 27 is a safe date.

I'm not completely sure where this comment applies, as we don't specifically discuss longevity of special-purpose compressors. However, we mention longevity of gzip, which, coincidentally, was also first released in 1993. As gzip's Wikipedia article (https://en.wikipedia.org/wiki/Gzip) mentions: "Initial release 31 October 1992; 27 years ago". Thus, we updated the number to 27.

> Please, improve the format of the figures and tables.

We improved figures and tables (as far as we saw a space for improvement).

> Some of the bullets have a final ".", others don't. Please, pick one and use the same format.

Thank you, we changed the lists to a consistent format. Namely, we use final "." in those bulleted lists where each entry is a complete sentence. In other bulleted lists, where entries are just items of the list,

we don't use final ".".

> Reviewer #2:

> General:

> The article is well constructed and has a good explanation of the use cases for different measurement criteria, such as archival, database retrieval, one-time transfers and memory usage.

> There is good attention to detail with specifying the exact versions and commit hashes of each tool, the parameters used (and their processing scripts), and references for downloading each data set. This aids reproducibility and importantly aids the use of this benchmark framework for future software authors.

> Probably this article came out of the analysis for "naf", by the same authors, demonstrating that nibble-packing plus zstd is an unexpectedly strong contender. However a benchmarking framework is a valid and useful piece of work in its own right. To this end, the authors not only provide the results and a useful website, but also the tools used for producing it permitting future tools to be validated against the same data sets using the same methods. This greatly improves the value of this work.

> Specifics:

> 1. The abstract is good. The assertion that most sequence datasets use gzip is valid, if disappointing. I checked the EMBL sequence archive, UniProt/SwissProt and NCBI's RefSeq, all of which are gzipped.

> The findings / conclusion part are also good, stressing the benchmark framework and presentation rather than recommending specific tools which seems appropriate.

> Language throughout is good.

Thank you very much for the time you spent reviewing our work and for encouraging comments.

> 2. The scope needs to be clearly spelt out.

> Specifically it is targeting genomic sequence datasets (eg the aforementioned EMBL sequence databank) and not DNA sequencing reads, hence no quality values either. This is interesting as it's a little bit of a different focus from several other benchmarks.

> It's also excluding reference based compression tools (eg GRS, GReEn, RLZ, CRAM). The line has to be drawn somewhere so I fully understand this, but the scope of what the article covers as well as what it doesn't cover should be more explicit.

Thank you for the suggestion. We have clarified the scope in the "Scope, compressors and test data" section (previously named "Compressors and test data").

> 3. Mentioning "DNA alignments" is a bit ambiguous as most people now think of output from an aligner such as bwa - ie SAM format. The format being used here is the earlier style of dash-padded sequence sets. Please clarify this distinction. I'm not sure what the proper term is, but I think "multiple sequence alignment" covers it.

Thank you for the suggestion. We have changed all mentions of alignment data to use "multiple sequence alignment" wording.

> 4. It is a little unclear precisely which data is being compressed.

> Obviously quality values are not as mention is made to adapting fastq compressors to the task. How about reference names? Other ancillary data after the reference name (oh how I loathe FASTA for that ill-defined mess). Is it purely sequence being evaluated, or the entire FASTA file? Do tools have to be case sensitive? Do they need to cope with ambiguity codes?

> The wrapper scripts cope with some of these things, but it is unclear if this is simply for purposes of testing the compression worked e.g. given the lack of support for lower case, or whether this

information is actually being included in the evaluation and added as a side-channel for tools that don't support it natively. If so, how is that done?

> Looking at the wrapper scripts it appears these other types of data get written to separate files and compressed with zstd. This needs documenting in the paper itself, along with an explanation of whether the size of those ancillary files is added to the compressed size, and also whether the time taken is included. (I am assuming yes, but please be explicit.)

Thank you for the suggestion. Indeed this was not clearly explained. We added long explanation in the "Methods" section, under "Streaming mode" and "FASTA format compatibility" headers.

Each compressor has to losslessly compress the entire full-featured FASTA file, including sequence names, case sensitivity and ambiguity codes. All compressors that lack native support for this, receive it via our wrappers. As you correctly assumed, the size of ancillary files, as well as time spent on pre-processing the FASTA stream and extracting these side channels (as well as adding them back during decompression) is counted as part of the total measurement.

Fortunately our wrappers are really fast and don't impact the results much for most compressors. However, all non-trivial wrappers (which means implementing anything more than streaming support) are benchmarked in "wrapper-only" mode and their results are included in benchmark database. Also fortunately those extra files are usually very small and compress well, so they don't impact overall compression rate much.

While admittedly not perfect, this seemed like the only viable strategy that would allow to compare the diverse array of compressors (each doing their own thing), and at the same time to have them doing a useful task (as opposed to compressing a raw stream of ACGT).

> 5. Tool selection.

> There are various fastq compression tools not benchmarked, including but not limited to FQSqueezer, Minicom, Orcom and FaStore. Are these planned? This is hinted at with "our study is not a one-off benchmark, but marks the start of a project where we will continue to add compressors and test data".

> However this is somewhat of a never ending task, as is alluded to with "Since it's impractical to benchmark every existing compressor, we will continue to only benchmark compressors selected based on their performance, quality and usefulness for sequence compression".

> If there are specific reasons why some tools were not evaluated then perhaps this should be mentioned on the website under rejected tools along with a reason (eg for speed, robustness, reordering of data).

Thank you for a good suggestion. Indeed some compressors are still missing in benchmark, each with their own reason. We've been keeping notes about all such potential additions, so it makes perfect sense to share those notes on the website. We now added the "Missing Compressors" page to the website, accessible from the "Compressors" page. Direct link: http://kirr.dyndns.org/sequence-compression-benchmark/?page=Missing-Compressors .

I believe the benchmark is currently reasonably thorough, but there will always be more compressors to test.

Regarding the mentioned tools:

FQSqueezer - has been added to the benchmark.

Minicom - has been added to the benchmark.

ORCOM - seems to always re-order the reads, making it incompatible with our conditions.

FaStore - seems to always re-order the reads, making it incompatible with our conditions.

I have to add that testing FASTQ compressors on FASTA data (via adding constant quality) is mainly of theoretical interest and probably has little practical value.

FASTQ compressors are usually designed under a FASTQ-specific set of assumptions, such as: "all reads are very short", "all reads are of same length", "order of reads does not matter", "all reads are sampled from underlying genome with substantial coverage". These assumptions don't hold in typical FASTA data and in our benchmark. So the results we obtain for FASTQ compressors may not transfer well to their performance on actual data they are designed for.

We added a mention of this to the "Scope" section on the "About" page on the website.

Still it's interesting to see how different approaches and compressors handle genomes and other FASTA datasets, so we will probably continue to benchmark FASTQ compressors.

> 6. Wrapper scripts/tools.

> How much time is in processing vs the actual tool? For example bsc.pl $cmd is little more than running bsc, while Quip's has 5 components piped together before piping into quip itself. Is the quip tool the bottleneck here and therefore the speed of the other bits irrelevant? I see most are in C, so it's possibly minimal impact, but it is hard to judge. If the impact is minimal, then it's probably best to acknowledge that it was measured and found to be insignificant.

Following this comment, we now also discuss this in the "FASTA format compatibility" part of the "Methods" section. In case when wrappers add anything other than streaming support, we benchmarked the "wrapper-only" runs, so that such runs can be compared with complete "wrapper+compressor" runs. This allows us to see how much of the time is consumed by the wrapper.

In most cases the impact is minimal. There are few cases where wrappers significantly impact compression or decompression speed. Such cases occur when 2 conditions overlap: 1) Compressor is very fast. 2) Compressor requires extensive data preprocessing. Notable examples are 2bit and DSRC.

This can be seen by including both the compressor and its wrapper in a scatterplot produced on benchmark website. We added links to such analyses to the "Examples" page on the website.

> Was CPU (user+system) time measured at all? If so then the ratio of wall clock to CPU time is a good indication of whether the pipeline is causing stalls or not.

Only total wall clock time was measured. I agree this could be interesting, but I don't expect much stalls. Could be interesting to try it some time.

One problem with such measurements is that I found that they influence speed of the fastest compressors. This is why, for example, memory use and speed are measured separately, using different runs of the same compressor.

> 7. Using fastq-from-sequence may mean that some tools has timings that aren't entirely comparable. While still a valid time for that tool, it's not indicative of the time for the sequence-only portion of that tool.

Yes, exactly. This is an inevitable consequence of testing FASTQ compressors on FASTA data, and it will remain until we add actual FASTQ data. Currently we are not sure whether we will be able to do it, but it's a possibility we consider for the future.

> I don't think there is much you can do to mitigate this bar rewriting other peoples code, so realistically it's just something that could be presented as a warning.

We added an explanation and a warning in the new "FASTQ Compressors" part of the "Methods" section. We also added corresponding warning in the "Scope" section on the "About" page on the website.

> 8. Be explicit as to the license on your software. Some had a license declaration (public domain) but not all.

Thanks, we specified a license (public domain) on the "Wrappers" page of the website.

> 9. A minor typographical: "[A] wide variety of charts can be produced..."

Thanks, fixed.

> Thank you for your work.

We sincerely appreciate your valuable comments on this manuscript.

Close
