# Supplementary material for: Sequence Compression Benchmark (SCB) database—A comprehensive evaluation of reference-free compressors for FASTA-formatted sequences
Source: Gigascience. 2020 Jul 6;9(7):giaa072. doi: 10.1093/gigascience/giaa072 (PMC7336184; doi:10.1093/gigascience/giaa072)
Supplement: giaa072_Response_to_Reviewer_Comments_Revision_1 [file giaa072_response_to_reviewer_comments_revision_1.pdf]

Author's Response To Reviewer Comments

Close

Dear Dr. Zauner,

Thank you very much for the quick response! I did all the requested changes. My replies are below.

Sincerely,
Kirill

> Before we proceed to acceptance, please consider the (very minor) follow-up comments of reviewer 1, below. Our publisher OUP will provide some copy-editing, but I think the reviewer is right that it would he helpful if you go over the manuscript again to correct typos, grammatical mistakes etc.

Thank you for suggestion. I did all changes suggested by reviewer 1. I also went over the manuscript again and corrected some more typos and improved wording and grammar in a few cases.

> Please also add a section "Availability of supporting source code and requirements" to share details about your benchmarking script and other supporting code. If the code is not hosted in a repository, the GigaScience GitHub repository is also available for this purpose.

> List the following:

> Project name: e.g. My bioinformatics project
> Project home page: e.g. https://github.com/ISA-tools
> Operating system(s): e.g. Platform independent
> Programming language: e.g. Java
> Other requirements: e.g. Java 1.3.1 or higher, Tomcat 4.0 or higher
> License: e.g. GNU GPL, FreeBSD etc.
> RRID: if applicable, e.g. RRID: SCR_014986

> The code needs to be under an Open Source Initiative approved license (see https://opensource.org/licenses).

I uploaded all code used for this study on GitHub: https://github.com/KirillKryukov/scb. I changed the manuscript text accordingly. I also removed the now redundant Supplementary Code from submission.

All code is in Public Domain.

> Also before acceptance, our data curators will contact you to prepare an archival copy of code (and supporting data) for our repository GigaDB. Please include a citation to your GigaDB dataset (including the DOI link) to your reference list, and cite this in the data availability section.

> Please follow this example format for the reference:

> [xx] Author1 N, Author2 N, AuthorX N. Supporting data for "Title of your manuscript". GigaScience Database. 2020. http://dx.doi.orgxxxxxxxxxxxx

> (You will get the doi link form our curators)

I added reference [68] according to this advice.

> In the "availability" section, please also include a sentence to refer to this GigaDB set. For example, write: "An archival copy of scripts [and supporting data] is also available via the GigaScience database GigaDB [xx]."

I changed the text according to this suggestion.

> Reviewer reports:

> Reviewer #1: The authors have addressed all of my concerns. Thank you for addressing all.
> The amount of time and dedication applied to this work is impressive. Thank you for your contribution.
> Regarding the 27-years, I made a mistake while I was reading (please, ignore that comment).
> Regarding the text, I would recommend a minor review in order to improve the description (for example, using an online editor program).
> Some of the possible changes are:
> * thorougly -> thoroughly;
> * general purpose -> general-purpose;
> * X axis -> x-axis;
> * offer large improvement -> offer a large improvement (similar characteristics exist in several places of the text);
> * also, some of the parts of the text (not very often) are somewhat dense and, hence, they could be simplified.

Thanks, fixed all these typos and went through the text trying to improve the text again.

Close
