# Supplementary material for: Sequence Compression Benchmark (SCB) database—A comprehensive evaluation of reference-free compressors for FASTA-formatted sequences
Source: Gigascience. 2020 Jul 6;9(7):giaa072. doi: 10.1093/gigascience/giaa072 (PMC7336184; doi:10.1093/gigascience/giaa072)
Supplement: giaa072_Response_to_Reviewer_Comments_Revision_2 [file giaa072_response_to_reviewer_comments_revision_2.pdf]

Author's Response To Reviewer Comments

Close

I am uploading the updated manuscript with the required changes.

>- Please add the DOI link of your GigaDB set to the bibliography (reference 68), exchanging it for the dummy link . The link to use is : http://dx.doi.org/10.5524/100762

Done.

>- For authors who have an ORCID ID, please add this to the title page

I have added ORCID IDs of all authors.

>- Please also ensure that your revised manuscript conforms to the journal style, which can be found in the Instructions for Authors on the journal homepage. (Note that "Methods" should follow "Findings", but before the declaration sections and bibliography etc).

I moved the Methods section as requested. I also checked the "Instructions to Authors" page again. I added the "Findings" title.

>- Please upload your manuscript file in editable format (word doc or tex, or whatever you used - it seems we only have a PDF so far)

I am uploading the manuscript in both PDF and MSWord formats.

Close
