# Supplementary material for: Sequence Compression Benchmark (SCB) database—A comprehensive evaluation of reference-free compressors for FASTA-formatted sequences
Source: Gigascience. 2020 Jul 6;9(7):giaa072. doi: 10.1093/gigascience/giaa072 (PMC7336184; doi:10.1093/gigascience/giaa072)
Supplement: giaa072_Reviewer_1_Report_Original_Submission — Diogo Pratas -- 3/3/2020 Reviewed [file giaa072_reviewer_1_report_original_submission.pdf]

Reviewer Report

Title: Sequence Compression Benchmark (SCB) database â€” a comprehensive evaluation of reference-free compressors for FASTA-formatted sequences

Version: Original Submission **Date:** 3/3/2020

Reviewer name: Diogo Pratas

Reviewer Comments to Author:

This article describes a benchmark for FASTA data that includes online material with a very high potential to be used by the genomic/proteomic data compression community. The benchmark is wide, balanced, and fair. The online tool for visualization of the benchmark is efficiently implemented and easy to follow. The benchmark includes a good set of tools. In general, the work reflects a high knowledge of the tools and bioinformatics background. However, some concerns first need to be addressed before entering in a much more detailed review mode.
Major concerns:
There are many compressors for many purposes. Choosing a compressor depends on the purpose. These purposes are not limited to fast decompression of good representations, namely to fast data transfer or integration with other tools. For example, long-term storage removes the importance of fast decompression and increases the importance over the compression ratio. The same can be seen for compressors that aim to approximate the Kolmogorov complexity, namely for genomic or proteomic analysis (phylogenomics, authentication, motif localization, rearrangements, among many others). Here, the importance in only at the efficiency of the compressor side using affordable (usually high) computational time and RAM.
Developing efficient genomic/proteomic compressors is also a methodology to improve unsupervised algorithms for data mining or machine learning. An example of this can be seen in the Hutter prize (http://prize.hutter1.net/), a half-million-dollar prize where compressors can spend up to 10GB of RAM and 100 hours to compress 1 GB of data. A version of the PAQ9 algorithm, which is comparatively a very "slow" program, is currently the state-of-the-art. Therefore, centering somewhat the results in NAF (which is perhaps the best industry-oriented FASTA compressor) and limiting the conclusions to the fastest decompression algorithms according to somewhat good compression capabilities does not entirely represent the field of genomic/proteomic data compression. This because FASTA data is already in post-processed state [semi-assembled (contig, scaffold), or assembled], unlike FASTQ. This exclusivity would make sense in FASTQ. Therefore, these notions and wider conclusions would make the manuscript stronger.
I also missed some protein sequence compressors, namely the recent protein compressor AC [AC: A Compression Tool for Amino Acid Sequences (https://link.springer.com/article/10.1007/s12539-019-00322-1)]. Sometimes, these are lost in a keyword search. A chain on amino acids can make a protein, therefore, the authors will find protein compressors defined as amino acid sequence compressors. The AC disadvantages: only for protein sequences (not FASTA), slower and, currently, RAM increases according to the redundancy and size of the sequence (but easily it can be adapted to a cache-hash).

Suggestion:
Given the current times, perhaps a very important dataset to add to the benchmark would be the whole viral database from the NCBI (FASTA format). It can be easily obtained from here:
https://www.ncbi.nlm.nih.gov/labs/virus/vssi
Minor:
From 1993 to 2020 there are 27 years, therefore, the longevity of special-purpose compressors is 27-year-old. Biocompress was already available in 1992, before the publication on the DCC (in march of 1993, after review). Therefore, it could also be 28, although 27 is a safe date.
Please, improve the format of the figures and tables.
Some of the bullets have a final ".", others don't. Please, pick one and use the same format.

Level of Interest

Please indicate how interesting you found the manuscript: Choose an item.

Quality of Written English

Please indicate the quality of language in the manuscript: Choose an item.

Declaration of Competing Interests

Please complete a declaration of competing interests, considering the following questions:

- Have you in the past five years received reimbursements, fees, funding, or salary from an organisation that may in any way gain or lose financially from the publication of this manuscript, either now or in the future?
- Do you hold any stocks or shares in an organisation that may in any way gain or lose financially from the publication of this manuscript, either now or in the future?
- Do you hold or are you currently applying for any patents relating to the content of the manuscript?
- Have you received reimbursements, fees, funding, or salary from an organization that holds or has applied for patents relating to the content of the manuscript?
- Do you have any other financial competing interests?
- Do you have any non-financial competing interests in relation to this paper?

If you can answer no to all of the above, write 'I declare that I have no competing interests' below. If your reply is yes to any, please give details below.

I declare that I have no competing interests

I agree to the open peer review policy of the journal. I understand that my name will be included on my report to the authors and, if the manuscript is accepted for publication, my named report including any

attachments I upload will be posted on the website along with the authors' responses. I agree for my report to be made available under an Open Access Creative Commons CC-BY license (http://creativecommons.org/licenses/by/4.0/). I understand that any comments which I do not wish to be included in my named report can be included as confidential comments to the editors, which will not be published.

Choose an item.

To further support our reviewers, we have joined with Publons, where you can gain additional credit to further highlight your hard work (see: https://publons.com/journal/530/gigascience). On publication of this paper, your review will be automatically added to Publons, you can then choose whether or not to claim your Publons credit. I understand this statement.

Yes Choose an item.
