# Supplementary material for: Sequence Compression Benchmark (SCB) database—A comprehensive evaluation of reference-free compressors for FASTA-formatted sequences
Source: Gigascience. 2020 Jul 6;9(7):giaa072. doi: 10.1093/gigascience/giaa072 (PMC7336184; doi:10.1093/gigascience/giaa072)
Supplement: giaa072_Reviewer_2_Report_Original_Submission — James Bonfield -- 3/3/2020 Reviewed [file giaa072_reviewer_2_report_original_submission.pdf]

Reviewer Report

Title: Sequence Compression Benchmark (SCB) database â€” a comprehensive evaluation of reference-free compressors for FASTA-formatted sequences

Version: Original Submission **Date:** 3/3/2020

Reviewer name: James Bonfield

Reviewer Comments to Author:

General:
The article is well constructed and has a good explanation of the use cases for different measurement criteria, such as archival, database retrieval, one-time transfers and memory usage.
There is good attention to detail with specifying the exact versions and commit hashes of each tool, the parameters used (and their processing scripts), and references for downloading each data set. This aids reproducibility and importantly aids the use of this benchmark framework for future software authors.
Probably this article came out of the analysis for "naf", by the same authors, demonstrating that nibble-packing plus zstd is an unexpectedly strong contender. However a benchmarking framework is a valid and useful piece of work in its own right. To this end, the authors not only provide the results and a useful website, but also the tools used for producing it permitting future tools to be validated against the same data sets using the same methods. This greatly improves the value of this work.
Specifics:
1. The abstract is good. The assertion that most sequence datasets use gzip is valid, if disappointing. I checked the EMBL sequence archive, UniProt/SwissProt and NCBI's RefSeq, all of which are gzipped.
The findings / conclusion part are also good, stressing the benchmark framework and presentation rather than recommending specific tools which seems appropriate.
Language throughout is good.
2. The scope needs to be clearly spelt out.
Specifically it is targeting genomic sequence datasets (eg the aforementioned EMBL sequence databank) and not DNA sequencing reads, hence no quality values either. This is interesting as it's a little bit of a different focus from several other benchmarks.
It's also excluding reference based compression tools (eg GRS, GReEn, RLZ, CRAM). The line has to be drawn somewhere so I fully understand this, but the scope of what the article covers as well as what it doesn't cover should be more explicit.
3. Mentioning "DNA alignments" is a bit ambiguous as most people now think of output from an aligner such as bwa - ie SAM format. The format being used here is the earlier style of dash-padded sequence sets. Please clarify this distinction. I'm not sure what the proper term is, but I think "multiple sequence alignment" covers it.
4. It is a little unclear precisely which data is being compressed.
Obviously quality values are not as mention is made to adapting fastq compressors to the task. How about reference names? Other ancillary data after the reference name (oh how I loathe FASTA for that ill-defined mess). Is it purely sequence being evaluated, or the entire FASTA file? Do tools have to be

case sensitive? Do they need to cope with ambiguity codes?
The wrapper scripts cope with some of these things, but it is unclear if this is simply for purposes of testing the compression worked e.g. given the lack of support for lower case, or whether this information is actually being included in the evaluation and added as a side-channel for tools that don't support it natively. If so, how is that done?
Looking at the wrapper scripts it appears these other types of data get written to separate files and compressed with zstd. This needs documenting in the paper itself, along with an explanation of whether the size of those ancillary files is added to the compressed size, and also whether the time taken is included. (I am assuming yes, but please be explicit.)
5. Tool selection.
There are various fastq compression tools not benchmarked, including but not limited to FQSqueezer, Minicom, Orcom and FaStore. Are these planned? This is hinted at with "our study is not a one-off benchmark, but marks the start of a project where we will continue to add compressors and test data". However this is somewhat of a never ending task, as is alluded to with "Since it's impractical to benchmark every existing compressor, we will continue to only benchmark compressors selected based on their performance, quality and usefulness for sequence compression".
If there are specific reasons why some tools were not evaluated then perhaps this should be mentioned on the website under rejected tools along with a reason (eg for speed, robustness, reordering of data).
6. Wrapper scripts/tools.
How much time is in processing vs the actual tool? For example bsc.pl $cmd is little more than running bsc, while Quip's has 5 components piped together before piping into quip itself. Is the quip tool the bottleneck here and therefore the speed of the other bits irrelevant? I see most are in C, so it's possibly minimal impact, but it is hard to judge. If the impact is minimal, then it's probably best to acknowledge that it was measured and found to be insignificant.
Was CPU (user+system) time measured at all? If so then the ratio of wall clock to CPU time is a good indication of whether the pipeline is causing stalls or not.
7. Using fastq-from-sequence may mean that some tools has timings that aren't entirely comparable. While still a valid time for that tool, it's not indicative of the time for the sequence-only portion of that tool.
I don't think there is much you can do to mitigate this bar rewriting other peoples code, so realistically it's just something that could be presented as a warning.
8. Be explicit as to the license on your software. Some had a license declaration (public domain) but not all.
9. A minor typographical: "[A] wide variety of charts can be produced..."
Thank you for your work.

Level of Interest

Please indicate how interesting you found the manuscript: Choose an item.

Quality of Written English

Please indicate the quality of language in the manuscript: Choose an item.

Declaration of Competing Interests

Please complete a declaration of competing interests, considering the following questions:

- Have you in the past five years received reimbursements, fees, funding, or salary from an organisation that may in any way gain or lose financially from the publication of this manuscript, either now or in the future?
- Do you hold any stocks or shares in an organisation that may in any way gain or lose financially from the publication of this manuscript, either now or in the future?
- Do you hold or are you currently applying for any patents relating to the content of the manuscript?
- Have you received reimbursements, fees, funding, or salary from an organization that holds or has applied for patents relating to the content of the manuscript?
- Do you have any other financial competing interests?
- Do you have any non-financial competing interests in relation to this paper?

If you can answer no to all of the above, write 'I declare that I have no competing interests' below. If your reply is yes to any, please give details below.

I declare that I have no competing interests

I agree to the open peer review policy of the journal. I understand that my name will be included on my report to the authors and, if the manuscript is accepted for publication, my named report including any attachments I upload will be posted on the website along with the authors' responses. I agree for my report to be made available under an Open Access Creative Commons CC-BY license (http://creativecommons.org/licenses/by/4.0/). I understand that any comments which I do not wish to be included in my named report can be included as confidential comments to the editors, which will not be published.

Choose an item.

To further support our reviewers, we have joined with Publons, where you can gain additional credit to further highlight your hard work (see: https://publons.com/journal/530/gigascience). On publication of this paper, your review will be automatically added to Publons, you can then choose whether or not to claim your Publons credit. I understand this statement.

Yes Choose an item.
